# Functionalization of a Triazine Dendrimer Presenting Four Maleimides on the Periphery and a DOTA Group at the Core

**DOI:** 10.3390/molecules21030335

**Published:** 2016-03-10

**Authors:** Changsuk Lee, Kun Ji, Eric E. Simanek

**Affiliations:** Department of Chemistry and Biochemistry, Texas Christian University, Fort Worth, TX 76129, USA; leecs73@hotmail.com (C.L.); kun.ji@tcu.edu (K.J.)

**Keywords:** dendrimer, triazine, DOTA, maleimide, peptide, bioconjugate, theranostic

## Abstract

A readily and rapidly accessible triazine dendrimer was manipulated in four steps with 23% overall yield to give a construct displaying four maleimide groups and DOTA. The maleimide groups of the dendrimer are sensitive to hydrolysis under basic conditions. The addition of up to four molecules of water can be observed via mass spectrometry and HPLC. The evolution in the alkene region of the ^1^H-NMR—the transformation of the maleimide singlet to the appearance of two doublets—is consistent with imide hydrolysis and not the Michael addition. The hydrolysis events that proceeded over hours are sufficiently slower than the desired thiol addition reactions that occur in minutes. The addition of thiols to maleimides can be accomplished in a variety of solvents. The thiols examined derived from cysteine and include the protected amino acid, a protected dipeptide, and native oligopeptides containing either 9 or 18 amino acids. The addition reactions were monitored with HPLC and mass spectrometry in most cases. Complete substitution was observed for small molecule reactants. The model peptides containing nine or eighteen amino acids provided a mixture of products averaging between 3 and 4 substitutions/dendrimer. The functionalization of the chelate group with gadolinium was also accomplished easily.

## 1. Introduction

**Chart 1:**
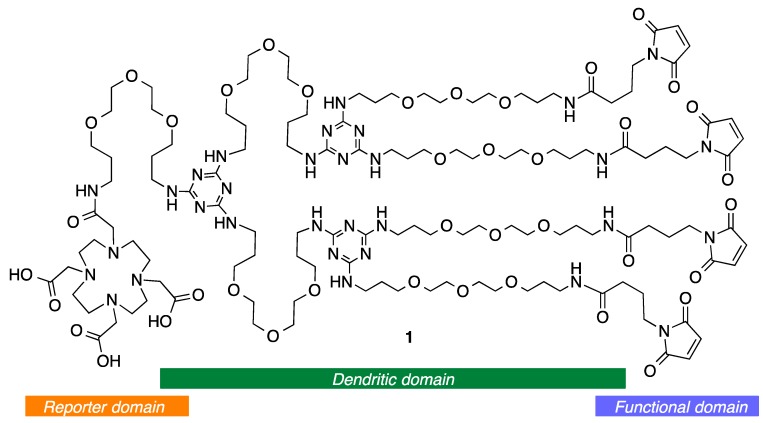
Target 1 with domains identified as reporter domain (**orange**), dendritic domain (**green**) and functional domain (**blue**).

The term “theranostics” describes molecules that offer both therapeutic and diagnostic capabilities [1,2,3,4,5,6]. Theranostic small molecules include a reporting group—commonly a chelate for MRI or PET imaging—ligated to a pharmacophore. Theranostic nanoparticles elaborate this design with materials that can offer multivalent displays of the therapeutic and/or diagnostic agent(s), as well as leverage additional properties conveyed with size, including changes in biodistribution and targeting. Theranostic nanoparticles come in many varieties including magnetic [7], silica [8,9,10], gold/quantum dots [11,12,13], graphene [14], liposomal [15,16], and polymeric [17,18]. Dendritic materials offer a compelling platform in this area [19,20] given the opportunity to exquisitely control chemistry to manipulate any number of properties displayed across multiple nanoparticle classes, so-called nanoperiodicity [21,22,23], including size, ligand density and solubility. Versatility is a key criterion for the design of such targets. Here, we report on a tetravalent platform, **1** (Chart 1). Target **1** comprises three different domains: a reporter domain, a functional domain, and a dendritic domain. The reporter domain presents a DOTA group that can host metals for diagnostic applications such as PET or MRI. The functional domain presents four maleimides that can be readily reacted with thiols. The functional domain is linked to the reporter domain through the dendritic domain. Here, the dendritic domain is a small, generation-1 triazine dendron.

## 2. Results and Discussion

### 2.1. Synthesis

**Scheme 1:**
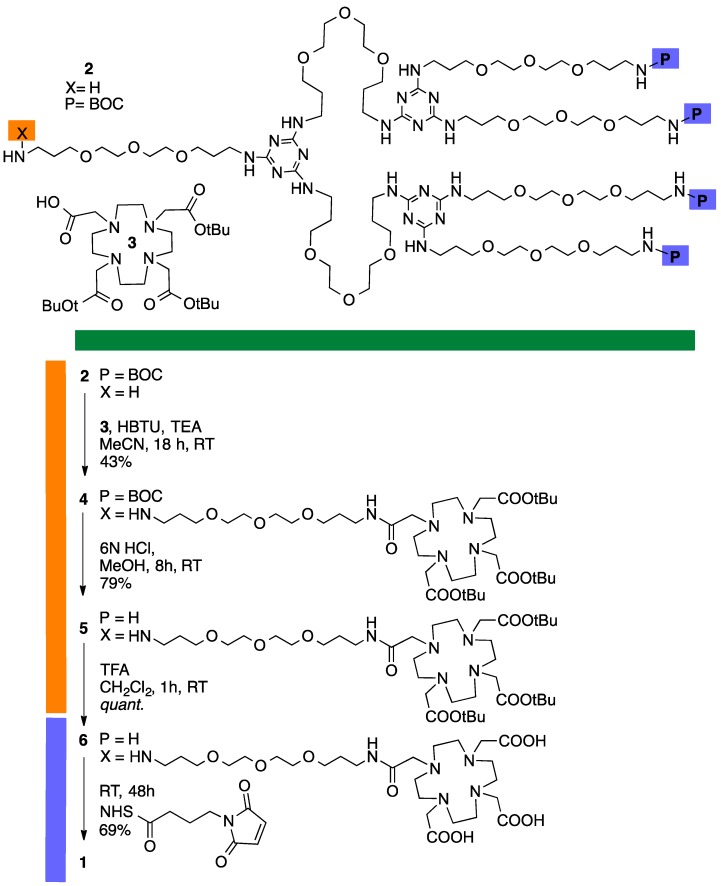
Synthesis of **1** commencing with reactions at the reported domain (**orange**) and then functional domain (**blue**).

The synthesis of **1** is shown in Scheme 1. It commences with the triazine dendron, **2**, which has been previously reported [24]. Intermediate **2** was obtained in less than 24 h using microwave-assisted reactions. The installation of the DOTA-reagent, **3**, was accomplished with HBTU to provide **4** in 43% yield. While mass spectrometry confirmed the structure of the product, the multiple conformations of the DOTA group led to broad signals in the ^1^H-NMR spectra. The lines corresponding to the seven expected *tert*-butyl groups were well resolved, and integration matched expectation when compared with signature regions of the dendrimer.

To probe generality, and assess opportunities for potential future efforts that elaborate the dendritic portion in the presence of the reporter domain, we explored the feasibility of a two-step deprotection strategy. First, the Boc groups of **4** were selectively removed with a 1:1 mixture of 6 N HCl:MeOH to afford **5** in 79% isolated yield. Second, intermediate **5** was elaborated into a larger G3 dendrimer with 16 terminal groups (see Supplementary Materials). Then, intermediate **6** was obtained directly from **4** with a global deprotection step using a 1:1 mixture of TFA:CH_2_Cl_2_. Finally, the installation of the maleimides to produce **1** proceeded with 69% yield. This four-step synthetic sequence (starting from **4**) is executed with 23% overall yield. Gadolinium can be incorporated into **1** at this point (see Supplementary Materials).

### 2.2. Hydrolytic Stability

The hydrolytic stability of **1** in an aqueous solution is of significant concern: Commercially available maleimide crosslinking reagents are moisture sensitive, especially at pH > 7.5. To assess, stability, **1** was added to slightly basic water (pH 7.5) and monitored by mass spectrometry and HPLC. After 4 h, the addition of up to four water molecules was clearly visible by both techniques. HPLC suggests that approximately 10% of **1** remains after 4 h under these slightly basic conditions. Under neutral conditions, however, the hydrolysis is much slower: Analysis of the ^1^H-NMR suggests only 25% hydrolysis, observed after 2 days.

^1^H-NMR of the hydrolysis product is consistent with imide hydrolysis to a ring-opened, maleic acid amide. The alkene singlet of **1** appearing at 6.7 ppm is replaced with two doublets at 6.2 and 5.8 ppm (*J* = 12.4 Hz). Accordingly, only the disappearance of *all* alkene signals during a substitution reaction can be considered evidence for complete substitution of **1** with ligands. The Michael addition reactions of the maleic acid amide derivatives with thiols were unsuccessful under the conditions employed for the conjugates described in the following paragraphs. This hydrolysis reaction—with similar kinetics—has been previously reported in functionalized PAMAM dendrimers [25].

### 2.3. Conjugation of Small Molecules

**Chart 2:**
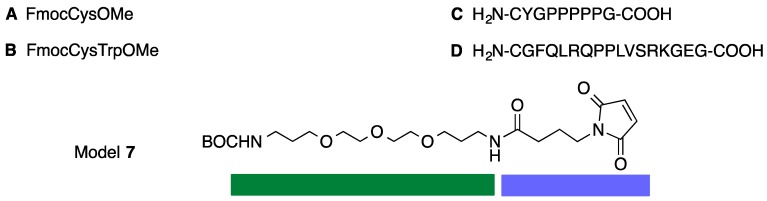
Thiols **A**–**D** in this study and model system **7** which models the dendritic domain (**green**) and functional domain (**blue**).

Fortunately, and in contrast to hydrolysis, the reaction between the maleimide and a thiol was rapid. The thiols and the model compound **7** that were examined in this study are shown in Chart 2. Hydrophobic **A** and **B** were chosen to examine solubility limitations that might be encountered during conjugation reactions. Oligomers **C** and **D** were chosen to assess the impacts of size on conjugation efficiency as determined by reaction times and product distributions. Since we predicted that **1** could serve as a building block for libraries, peptides **C** and **D** were used without extensive purification. Specifically, HPLC and MS analysis suggest that each peptide is present in greater than 60% in the crude cocktail obtained after precipitation of the cleavage product derived from a peptide synthesizer. To identify conjugates, the letter indicating the thiol and valency (when appropriate) is appended to the parent maleimide. That is, reaction of **7** and **A** produces **7**-**A_1_** (or just **7**-**A**), whereas the product of three additions of **D** to **1** is identified as **1**-**D_3_**.

Compound **7** was prepared as a model to identify indicators of a successful reaction (Chart 2). The reactions of **7** with **A** (FmocCysOMe) and **B** (FmocCysTrpOMe) to yield conjugates **7**-**A** and **7**-**B**, respectively, were followed using NMR spectroscopy, mass spectrometry, and HPLC. Here, methylene chloride was used as a reaction solvent due to the limited water solubility of the protected amino acids. NMR spectroscopy provides convenient handles for assessing conjugation. The ^1^H-NMR spectra showed a shift of the broad multiplet of the β-CH_2_ of cysteine from ~3.0 ppm to 3.2 and 3.5 ppm with a pronounced diastereotopic splitting. Unfortunately, this region of the spectra was congested. The signals generated upon addition to the maleimide were more diagnostic. The methylene of the thioether appeared at 3.8 ppm. The adjacent methylene split into a diastereotopic pair at ~2.5 ppm (in an uncrowded region of the spectrum) and 3.1 ppm (in a more crowded region). The ^13^C-NMR was used to corroborate the addition. The appearance of diastereomers corresponding to the maleimide thioether (~39ppm), imide methylene (~36 ppm) and β-CH_2_ of the cysteine residue (~34 ppm) were diagnostic for successful conjugation of the chiral thiol. The data from mass spectrometry and HPLC were consistent with a clean reaction between **7** and **A** or **B**. Mass spectrometry of the conjugates of **7**-**A** and **7**-**B** showed the parent ion with minor ions corresponding to the loss of both the Boc and Fmoc groups. Both **7**-**A** and **7**-**B** were isolated using column chromatography and obtained with approximately 60% yields. These low yields are attributed to the loss of Fmoc group during isolation.

Oxidation of the thiol offered the potential for a competing side-reaction. The cysteine disulfide of **A** appeared at 3.25 ppm in a crowded region of the ^1^H-NMR spectrum. However, HPLC analysis provided a clear indication of this compound: The disulfides, thiols and conjugation products **7**-**A** and **7**-**B** showed unique retention times (See Supplementary Materials). Oxidation to the disulfide did not appear to be responsible for low yields of isolated materials. Accordingly, no special handling procedures were adopted for latter conjugation reactions.

Advantageously, we were able to perform conjugations with **1** in a range of solvents, because **1** was readily soluble in chloroform, dichloromethane, dioxane, methanol and water. For example, reactions of **1** with hydrophobic models **A** and **B** were performed in a dioxane:dichloromethane mixture. A dioxane:water mixture was used for nonapeptide **C**. For the hydrophilic peptide **D**, the conjugation was performed in water.

**Figure 1:**
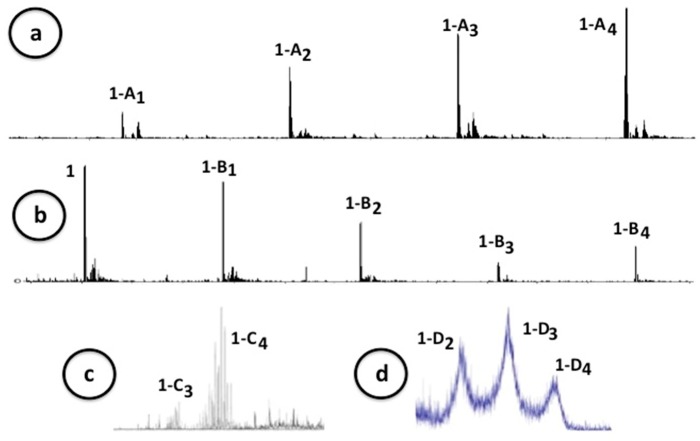
Conjugates **1** with **A** (**a**); **B** (**b**); **C** (**c**) and **D** (**d**). Traces (**a**,**b**) are taken upon the addition of thiol by ESI-TOF MS. Mass scales for these traces are *m*/*z* 2900-4400 (**a**) and *m*/*z* 2600-5200 (**b**); Traces (**c**,**d**) are taken of the purified product mixture. Mass scales for these traces are *m*/*z* 4500–7800 (**c**) and *m*/*z* 5000–13000 (**d**). Trace (**d**) was obtained by MALDI-TOF-MS.

Conjugates **1**-**A_4_** and **1**-**B_4_** were isolated using chromatography with 65% and 54% yields. Mass spectrometry, and HPLC and NMR spectroscopy all provided corroborating data. HPLC showed that the retention times of thiol **A** and the conjugation product **1**-**A_4_** were different by approximately 1 min. However, thiol **B** and the product of conjugation, **1**-**B_4_**, showed similar retention times. The conjugation reaction using **A** and **B** were readily monitored by electrospray ionization-time of flight mass spectrometry, ESI-TOF MS (Figure 1a,b). Immediately upon mixing reagents, mass spectrometry showed clear evidence of conjugate formation with amino acid **A** reacting faster than dipeptide **B**.

### 2.4. Conjugation of Pepties

The products of conjugation of **1** with nonapeptide **C** were isolated in a multistep sequence. First, the reaction mixture was concentrated to an oil. The oil was then resuspended in deionized water to facilitate membrane filtration with a cellulose centrifugation filter (Ultracell YM3) with a 3 kDa molecular weight cutoff. After multiple rinses, the recovered volume was lyophilized to dryness. Using ^1^H-NMR, a comparison of the integration of the aromatic signals of the tyrosine with signals derived from the methylene, which was derived from the maleimide at 2.5 ppm (dendrimer), suggests that the product distribution approached the desired **1**-**C_4_** target, approximately ~**1**-**C_3.75_**. Mass spectrometry confirmed a successful reaction (Figure 1c). The presence of multiple lines at lower molecular weights than the target, **1**-**C_4_**, is consistent with the use of an impure peptide.

**Figure 2:**
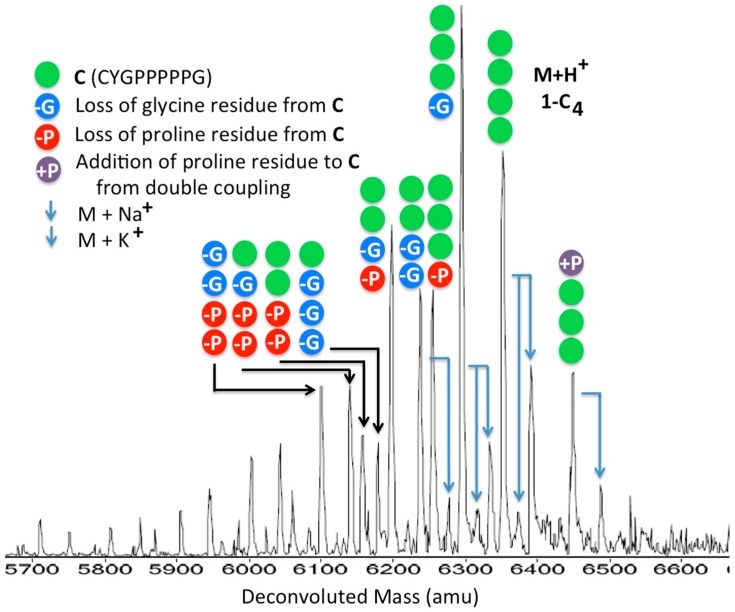
Tentative assignment of the products of conjugation of peptide **C** with **1** to yield **1-C_4_**.

Not surprisingly, these peaks were assignable (Figure 2). The desired product containing four copies of **C** is indicated with *four* green dots. Adducts of **1**-**C_4_** with Na^+^ and K^+^ are shown with blue arrows. For lines corresponding to products with lower molecular weights, one or more of the peptides are missing amino acids. The most abundant ion displays a peptide missing a glycine residue (blue dot). Peptides missing proline (red dot) are also present. For simplicity, we indicate the minimum number of deletions for each peptide. That is, whether the final compound has one deletion in each peptide—or comprises three perfect peptides **C** and a peptide missing four amino acids—is unknown. The necessity of using double couplings during the solid phase synthesis of this proline-rich sequence is also reflected by the presence of a peak corresponding to a product with three copies of **C** and one copy of **C** containing an extra proline residue (purple circle). Given the distribution of products, it is unsurprising that the HPLC trace is broad.

The conjugation of 16-amino acid peptide, **D**, with **1** was followed using HPLC by monitoring the consumption of **1** and the appearance of new species. The reaction products were purified by membrane dialysis. MALDI-TOF mass spectrometry of the isolated product showed peaks corresponding to **1**-**D_2_**, **1**-**D_3_**, and **1**-**D_4_**. The broadness of the mass spectrum is consistent with both the use of impure peptides and the complications arising from salts of this highly charged peptide. The ^1^H-NMR spectrum—albeit broad—showed neither maleimide nor maleic acid signals present in the reaction mixture as might be expected from the perceived low conversion rate. Although thiol addition should be faster than that the addition of any other functional group or solvent, steric congestion could retard this desired reaction and afford opportunities for intermolecular reaction of maleimides with amino acid side chains like that of lysine. The extent to which these reaction can occur as a function of lysine residue position will be assessed in a future study. Using ^1^H-NMR, a comparison of peak areas derived from the aromatic phenylalanine peaks and methylene of maleimide suggests that the product distribution is centered at **1**-**D_3.5_**, a more favorable ratio than what might be expected from casual inspection of the mass spectrum.

## 3. Experimental Section

### 3.1. General Synthetic Procedures

All chemicals were purchased from Aldrich (St. Louis, MO, USA) and Acros (Fair Lawn, NJ, USA) and used without further purification. All solvents were ACS grade and used without further purification. HPLC was carried out using an Agilent Technologies (Santa Clara, CA, USA) 1260 Infinity system and an Agilent Technologies 1260 Infinity DAD detector. NMR spectra were recorded on a Bruker Ascend 400 MHz spectrometer (Billerica, MA, USA) in CDCl_3_, CD_3_OD, or D_2_O. All ESI mass spectral analyses were carried out by an Agilent Technologies 6224 TOF LC/MS system.

The chromatographic system used to measure sample purity consisted of a degasser (Agilent G1379B, Palo Alto, CA, USA), capillary pump (Agilent G1312B), micro well-plate auto sampler (Agilent G1367D), eclipse XDB-C18 column (4.6 mm i.d. × 150 mm, 5 μm, Agilent), and a diode array detector (Agilent G1316B). The mobile phase consisted of water/acetonitrile (A/B, HPLC grade, 0.1% (*w*/*v*) trifluoroacetic acid) at a flow rate of 0.8 mL/min. The elution gradient was 10% MeCN for 5 min, ramp to 90% MeCN in 30 min, and ramp down to 10% MeCN in 15 min. The sample volume injected 5 μL at a concentration 0.1 mg/mL with HPLC-grade MeCN, and detected at 214 nm.

### 3.2. Synthesis of Dendrimer ***1***

**Figure 3:**
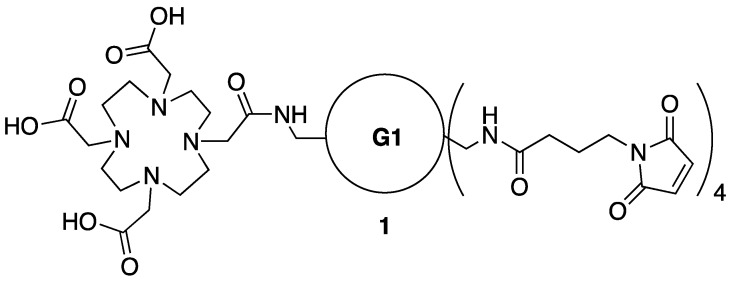
Intermediate **1**.

To a solution of **6** (31 mg, 0.014 mmol) and DIPEA (20 μL, 0.115 mmol) in dioxane (1 mL), a solution of maleimide–NHS ester (32 mg, 0.115 mmol) in dichloromethane (1 mL) was added at RT then stirred for 48 h. The reaction was concentrated and purified by column chromatography (DCM:MeOH = 97:5 → DCM:MeOH = 8:2) to give **1** (Figure 3) as a foam (28 mg, 69%). ^1^H-NMR (400 MHz, CDCl_3_): δ 6.71 (s, 8H), 3.64–3.32 (m, 120H, CH_2_OCH_2_CH_2_OCH_2_CH_2_OCH_2_, C_3_N_3_-NHCH_2_CH_2_CH_2_O, DOTA-CONHCH_2_CH_2_CH_2_O, CONHCH_2_CH_2_CH_2_N, Maleimide-CONHCH_2_CH_2_CH_2_O), 2.15 (t, *J* = 7.2, 8H), 1.92 (dt, *J* = 14, 7.2, 8H), 1.89–1.77 (br m, 28H, OCH_2_CH_2_CH_2_); ^13^C-NMR (100 MHz, CDCl_3_) δ 171.7, 170.9, 134.1, 70.5 (OCH_2_CH_2_O), 70.2 (OCH_2_CH_2_O), 70.1 (OCH_2_CH_2_O), 69.8, 69.2 (CH_2_CH_2_CH_2_O), 38.1 (NH_2_CH_2_CH_2_CH_2_O), 37.7 (CH_2_CH_2_CH_2_O), 37.3 (maleimide), 33.6 (maleimide), 29.5 (NH_2_CH_2_CH_2_CH_2_O), 29.0 (NH_2_CH_2_CH_2_CH_2_O), 24.8 (maleimide); MS (ESI-TOF) calcd. for C_127_H_214_N_31_O_40_ 2813.5664, found 2813.5751 [M + H]^+^. Spectra appear in the Supplementary Materials: Figures S1–S5.

### 3.3. Synthesis of Intermediate S1—Maleic Acid Amide of ***1***

**Figure 4:**
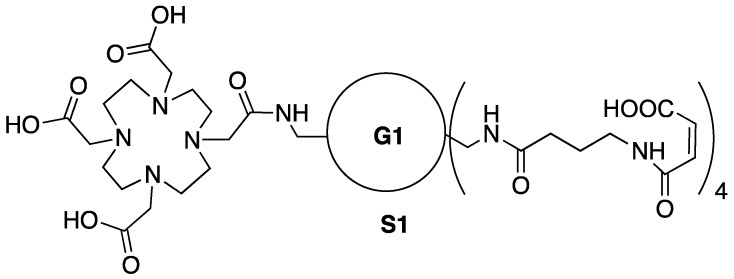
Intermediate **S1**.

A solution of **1** (8 mg) in water (1 mL) was adjusted to pH 7–8 with 1 N NaOH at RT then stirred for 18 h. The reaction mixture was lyophilized to give Intermediate **S1** (Figure 4). ^1^H-NMR (400 MHz, D_2_O): δ 6.23 (d, *J* = 12.4 , 4H), 5.83 (d, *J* = 12.4, 4H), 3.55–3.12 (m, 120H, CH_2_OCH_2_CH_2_OCH_2_CH_2_OCH_2_, C_3_N_3_-NHCH_2_CH_2_CH_2_O, DOTA-CONHCH_2_CH_2_CH_2_O, CONHCH_2_CH_2_CH_2_N, Maleimide-CONHCH_2_CH_2_CH_2_O), 2.18 (t, *J* = 7.4, 8H), 1.89–1.77 (br m, 36H, OCH_2_CH_2_CH_2_, Maleimide); ^13^C-NMR (100 MHz, D_2_O) δ 180.0 (DOTA-acid), 179.9 (DOTA-acid), 175.7 (Maleimide-CONHCH_2_CH_2_CH_2_O), 174.3 (DOTA-amide), 173.2 (DOTA-acid), 168.1 (conjugate amide), 165.3 (triazine, conjugate acid), 135.7, 124.9, 69.7, 69.6, 69.5, 69.4, 68.6, 68.4, 38.5 , 37.4, 37.3,36.4, 33.1, 28.9, 28.3, 24.9 (maleimide); MS (ESI-TOF) calcd. for C_127_H_222_N_31_O_44_ 2885.6087, found 2885.6806 [M + H]^+^. Spectra appear in the Supplementary Materials: Figures S6–S9.

### 3.4. Synthesis of Intermediate ***4***

**Figure 5:**
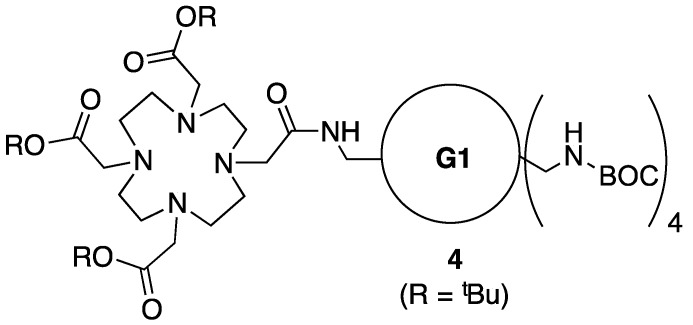
Intermediate **4**.

To a solution of **2** (194 mg, 0.09 mmol) and **3** (52 mg, 0.09 mmol) in acetonitrile (8 mL), HBTU (34 mg, 0.09 mmol) was added followed by triethylamine (25 μL, 0.18 mmol) at RT. The reaction was stirred for 18 h. The reaction was diluted with 30 mL dichloromethane and washed 0.1 N HCl (30 mL), 10% NaHCO_3_ (30 mL), brine (30 mL), then dried over MgSO_4_, and concentrated. The crude product was purified by column chromatography (DCM:MeOH = 97:5 → DCM:MeOH = 85:15) to give **4** (Figure 5) as a foam (105 mg, 43%). ^1^H-NMR (400 MHz, CDCl_3_): δ 3.68–3.47 (m, 104H, CH_2_OCH_2_CH_2_OCH_2_CH_2_OCH_2_, C_3_N_3_-NHCH_2_CH_2_CH_2_O , Dota-CONHCH_2_CH_2_CH_2_O), 3.22–3.18 (br m, 8H, BocNHCH_2_), 1.84–1.72 (m, 28H, OCH_2_C**H_2_**CH_2_), 1.46 (s, 9H), 1.45 (s, 9H), 1.42 (s, 45H); ^13^C-NMR (100 MHz, CDCl3) δ 176.6 (DOTA-O**C**O^t^Bu), 174.4 (DOTA-O**C**O^t^Bu), 172.3 (DOTA-O**C**O^t^Bu), not found (**C**_3_N_3_), 156.0 (NH**C**O^t^Bu), 81.7 (DOTA-O**C**(CH_3_)_3_), 78.7 (**C**(CH_3_)_3_), 70.47 (O**C**H_2_**C**H_2_O), 70.21 (O**C**H_2_**C**H_2_O), 70.17 (O**C**H_2_**C**H_2_O), 70.10 (O**C**H_2_**C**H_2_O), 69.42 (CH_2_CH_2_**C**H_2_O), 69.02 (CH_2_CH_2_**C**H_2_O), 57.5 (DOTA), 56.2 (DOTA), 55.6 (DOTA), 53.5 (DOTA), 41.9 (NH_2_**C**H_2_CH_2_CH_2_O), 38.5 (**C**H_2_CH_2_CH_2_O), 38.4 (**C**H_2_CH_2_CH_2_O), 29.6 (NH_2_CH_2_**C**H_2_CH_2_O), 29.3 (NH_2_CH_2_**C**H_2_CH_2_O), 28.4 (C(**C**H_3_)_3_), 27.94 (DOTA-OC(**C**H_3_)_3_), 27.87 (DOTA-OC(**C**H_3_)_3_); MS (ESI-TOF) calcd. for C_127_H_242_N_27_O_36_ 2721.7936, found 2721.8117 [M + H]^+^. Spectra appear in the Supplementary Materials: Figures S11–S13.

### 3.5. Synthesis of Intemediate ***5***

**Figure 6:**
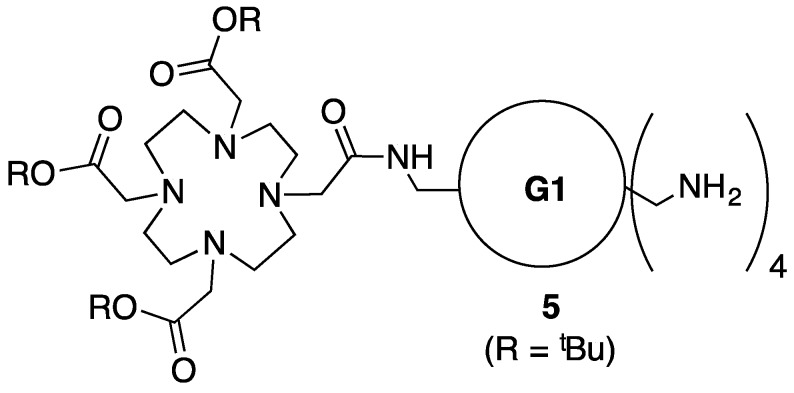
Intermediate **5**.

To a solution of **4** (59 mg, 0.022 mmol) in methanol (2 mL), 6 N HCl (aq) (2 mL) was added at RT then stirred for 8 h. The reaction was diluted with ethyl acetate (10mL) and water (2 mL). Water layer was basifying with 5 N NaOH (aq) then the desired compound was extracted with dichloromethane (10 mL × 8). Combined organic layers was dried over MgSO_4_, and concentrated to give **5** (40.5 mg of as a clear oil; Figure 6). ^1^H-NMR (400 MHz, CDCl_3_): δ 3.63–3.42 (m, 104H, CH_2_OCH_2_CH_2_OCH_2_CH_2_OCH_2_, C_3_N_3_-NHCH_2_CH_2_CH_2_O, DOTA-CONHCH_2_CH_2_CH_2_O), 3.10 (br s, 8H, NH_2_CH_2_), 1.96–1.82 (br m, 28H, OCH_2_CH_2_CH_2_), 1.45 (s, 27H); ^13^C-NMR (100 MHz, CDCl3) δ 172.5 (DOTA-O**C**O^t^Bu), 171.5 (DOTA-O**C**O^t^Bu), 165.8 (**C**_3_N_3_), 81.8 (DOTA-O**C**(CH_3_)_3_), 81.7 (Dota-O**C**(CH_3_)_3_), 70.54 (O**C**H_2_**C**H_2_O), 70.42 (O**C**H_2_**C**H_2_O), 70.19 (O**C**H_2_**C**H_2_O), 70.11 (O**C**H_2_**C**H_2_O), 69.95 (O**C**H_2_**C**H_2_O), 69.40 (CH_2_CH_2_**C**H_2_O), 69.27 (CH_2_CH_2_**C**H_2_O), 56.1 (Dota), 55.7 (Dota), 55.6 (DOTA), 39.0 (NH_2_**C**H_2_CH_2_CH_2_O), 38.1 (**C**H_2_CH_2_CH_2_O), 29.7 (NH_2_CH_2_**C**H_2_CH_2_O), 29.5 (NH_2_CH_2_**C**H_2_CH_2_O), 29.1 (NH_2_CH_2_**C**H_2_CH_2_O), 28.10 (DOTA-OC(**C**H_3_)_3_), 27.98 (DOTA-OC(**C**H_3_)_3_); MS (ESI-TOF) calcd for C_107_H_210_N_27_O_28_ 2321.5839, found 2321.6677 [M + H]^+^. Spectra appear in the Supplementary Materials: Figures S14–S16.

### 3.6. Synthesis of Intermediate—Generation 3 Dendrimer with DOTA

**Figure 7:**
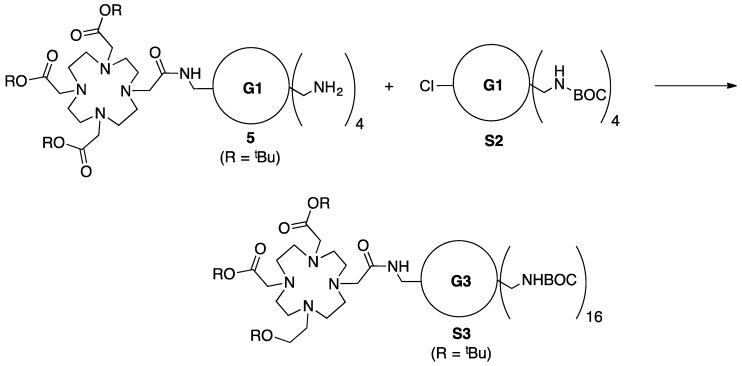
Synthesis of Intermediate **S3**.

A solution of **5** (79 mg, 0.034 mmol) and **M** (Macromonomer [24]) (542 mg, 0.27), and DIPEA (71 mL, 0.41 mmol) in THF (3 mL), dioxane (1 mL), *i*-propanol (70%) (0.5 mL) was stirred at 75 °C in a capped vessel for 4 d. The solution was evaporated under vacuum. The residue was dissolved in dichloromethane, washed with brine, dried over MgSO_4_, and concentrated. The crude product was purified by column chromatography. The crude product was purified by column chromatography (DCM:MeOH = 97:5 → DCM:MeOH = 85:15) to give **S3** (Figure 7) as a foam (92 mg, 27%). ^1^H-NMR (400 MHz, CDCl_3_): δ 3.65–3.42 (m, 464H, CH_2_OCH_2_CH_2_OCH_2_CH_2_OCH_2_, C_3_N_3_-NHCH_2_CH_2_CH_2_O, Dota-CONHCH_2_CH_2_CH_2_O), 3.22–3.19 (br m, 32H, BocNHCH_2_), 1.81–1.72 (br m, 124H, OCH_2_CH_2_CH_2_), 1.47 (s, 9H), 1.45 (s, 9H), 1.44 (s, 9H), 1.42 (s, 144H); ^13^C-NMR (100 MHz, CDCl3) δ not found (DOTA-O**C**O^t^Bu), not found (DOTA-O**C**O^t^Bu), not found (DOTA-O**C**O^t^Bu), not found (**C**_3_N_3_), 156.1 (NH**C**O^t^Bu), 81.85 (DOTA-O**C**(CH_3_)_3_), 81.79 (DOTA-O**C**(CH_3_)_3_), 78.8 (**C**(CH_3_)_3_), 70.56 (O**C**H_2_**C**H_2_O), 70.28 (O**C**H_2_**C**H_2_O), 70.23 (O**C**H_2_**C**H_2_O), 70.19 (O**C**H_2_**C**H_2_O), 69.52 (CH_2_CH_2_**C**H_2_O), 69.22 (CH_2_CH_2_**C**H_2_O), 69.17 (CH_2_CH_2_**C**H_2_O), not found (DOTA), not found (DOTA), not found (DOTA), 53.4 (DOTA), 41.8 (NH_2_**C**H_2_CH_2_CH_2_O), 38.5 (**C**H_2_CH_2_CH_2_O), 38.2 (**C**H_2_CH_2_CH_2_O), 29.67 (NH_2_CH_2_**C**H_2_CH_2_O), 29.60 (NH_2_CH_2_**C**H_2_CH_2_O), 29.54 (NH_2_CH_2_**C**H_2_CH_2_O), 29.33 (NH_2_CH_2_**C**H_2_CH_2_O), 28.5 (C(**C**H_3_)_3_), 28.01 (DOTA-OC(**C**H_3_)_3_), 27.94 (Dota-OC(**C**H_3_)_3_); MS (ESI-TOF) calcd for C_463_H_878_N_111_O_132_ 10106.5403, found 10114.2078 [M + H]^+^. Spectra appear in the Supplementary Materials: Figures S17–S19.

### 3.7. Synthesis of Intermediate ***6***

**Figure 8:**
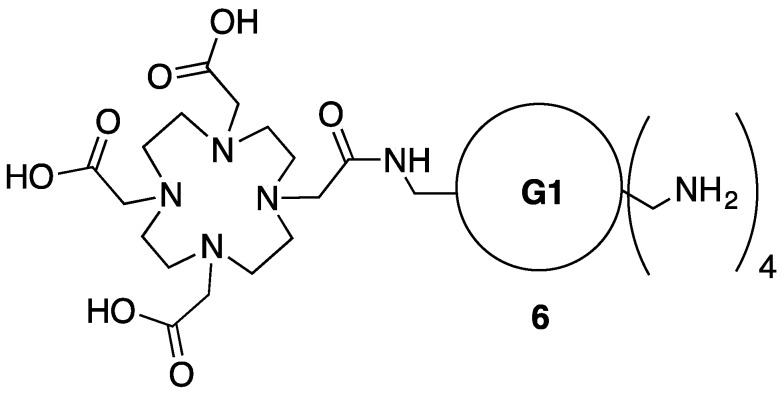
Intermediate **6**.

To a solution of **5** (68 mg, 0.029 mmol) in dichloromethane (2 mL), trifluoroacetic acid (2 mL) was added at RT then stirred for 20 h. Susbequently the reaction mixture was evaporated under vacuum. The residue was decanted with diethyl ether (5 mL × 3), and dried under vacuum to give **6** (Figure 8) as a white powder (quantitative). ^1^H-NMR (400 MHz, CD_3_OD): δ 3.55–3.26 (m, 104H, CH_2_OCH_2_CH_2_OCH_2_CH_2_OCH_2_, C_3_N_3_-NHCH_2_CH_2_CH_2_O , DOTA-CONHCH_2_CH_2_CH_2_O), 2.99 (br t, *J* = 6.2, 8H, NH_2_CH_2_), 1.85–1.76 (br m, 28H, OCH_2_CH_2_CH_2_); ^13^C-NMR (100 MHz, CD_3_OD) δ 164.8, 157.4 156.4, 71.6, 71.53, 71.50, 71.33, 71.23, 71.21, 70.23, 69.91, 69.76, 69.73, 39.87 , 39.64, 39.44, 39.35, 39.27, 39.19, 38.09, 38.03, 30.86, 30.45, 30.35, 30.19, 30.12; MS (ESI-TOF) calcd. for C_95_H_186_N_27_O_28_ 2153.3961, found 2153.4295 [M + H]^+^. Spectra appear in the Supplementary Materials: Figures S20–S22.

### 3.8. Synthesis of Model ***7***

**Figure 9:**
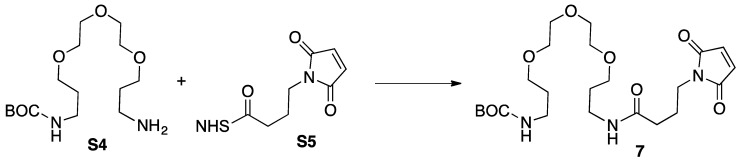
Synthesis of **7**.

To a solution of commercially-available maleimide succinimide ester **S5** (17 mg, 0.06 mmol) in dichloromethane (3.0 mL), a solution of monoBoc amine **S4** (16 mg, 0.05 mmol) in dichloromethane (1.0 mL) was added at RT. The solution was stirred for 3h at RT and evaporated under vacuum. The crude product was purified by column chromatography (DCM:MeOH = 99:1 → DCM:MeOH = 97:3) to give **7** (Figure 9) as a solid (21 mg, 86%). ^1^H-NMR (400 MHz, CDCl_3_): δ 6.71 (s, 2H, n), 3.67–3.53 (m, 14H, c, d, e, f, g, h, m), 3.36 (q, *J* = 6.4, 2H, j), 3.23 (q, *J* = 6.4, 2H, a), 2.16 (t, *J* = 6.4, 2H, k), 1.97–1.94 (m, 2H, l), 1.82-1.75 (m, 4H, b, i), 1.43 (s, 9H, Boc) ^13^C-NMR (100 MHz, CDCl_3_) δ 171.6, 170.8, 156.0, 134.1, 78.9, 70.5, 70.4, 70.2, 70.1, 69.8, 69.5, 38.4, 37.8, 37.2, 33.6, 29.6, 28.9, 28.4, 24.7; MS (ESI-TOF) calcd. for C_23_H_40_N_3_O_8_ 486.2815, found 486.4100 [M + H]^+^. Spectra appear in the Supplementary Materials: Figures S23–S25.

### 3.9. Synthesis of Conjugate ***7-A***

**Figure 10:**
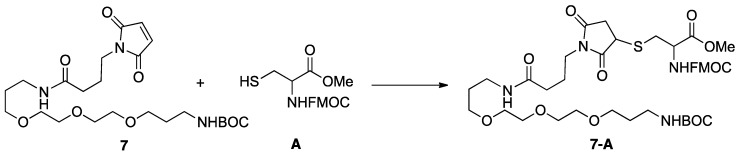
Synthesis of **7-A**.

To a solution of **1** (35 mg, 0.07 mmol) in dioxane (1.0 mL) a solution of cysteine **A** (28 mg, 0.079) in dichloromethane (1.0 mL) was added at RT. The solution was stirred for 48 h at room temperature and evaporated under vacuum. The crude product was purified by column chromatography (DCM = 100 → DCM:MeOH = 95:5) to give **7-A** (Figure 10) as a oil (37 mg, 61%). ^1^H-NMR (400 MHz, CDCl_3_): δ 7.76 (d, *J* = 8.0, 2H, FMOC), 7.61 (t, *J* = 6.0, 2H, FMOC), 7.42–7.28 (m, 4H, FMOC), 5.00 (br s, 1H, NH), 4.71–4.68 (m, 1H, p), 4.49–4.41 (m, 2H, FMOC), 4.24 (t, *J* = 6.8 , 1H, FMOC), 3.83–3.75 (m, 1H, n), 3.80 (s, 3H, q), 3.65–3.51 (m, 14H, c, d, e, f, g, h, m), 3.46 (dd, *J* = 14, 6.0, 1H, o), 3.37–3.31 (m, 2H, j), 3.23–3.19 (m, 2H, a), 3.15–3.03 (m, 2H, n’, o), 2.50–2.39 (m, 1H, n’), 2.15 (t, *J* = 7.2, 2H, k), 1.99–1.90 (m, 2H, l), 1.80–1.75 (m, 4H, b, i), 1.43 (s, 9H, BOC) ^13^C-NMR (100 MHz, CDCl_3_) δ 176.7, 174.5, 174.3, 171.5 (ester), 170.9 (amide), 156.0 (Boc), 155.9 (FMOC), 143.8, 143.7, 143.6, 141.3, 127.7, 127.1, 127.0, 125.1, 125.0, 119.98, 119.94, 70.4, 70.1, 70.0, 69.7, 69.4, 67.1, 52.9, 52.8, 47.1, 39.6, 38.6, 38.5, 38.4, 37.7, 36.0, 35.6, 33.5, 33.4, 29.6, 28.9, 28.4, 23.6, 23.55; MS (ESI-TOF) calcd. for C_42_H_59_N_4_O_12_S 843.3850, found 843.5872 [M + H]^+^. Spectra appear in the Supplementary Materials: Figures S26–S28.

### 3.10. Synthesis of Cystine *(**S6**)*

**Figure 11:**
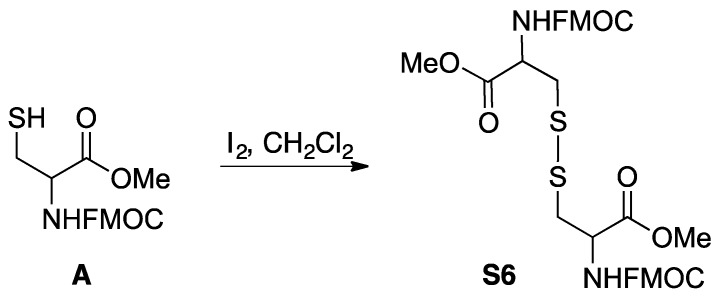
Synthesis of **S6**.

To a solution of **A** (59 mg, 0.165 mmol) in dichloromethane (4.0 mL), solid iodine (10 mg, 0.082 mmol) was added at RT. The solution was stirred for 2 h at room temperature and evaporated under vacuum. The crude product was purified by column chromatography (Ethyl Acetate: Hexanes = 1:9 → Ethyl Acetate: Hexanes = 3:7) to give **S6** (Figure 11) as white powder (46 mg, 39%). ^1^H-NMR (400 MHz, CDCl_3_): δ 7.74 (d, *J* = 7.2, 2H, FMOC), 7.59-7.57 (br s, 2H, FMOC), 7.39–7.27 (m, 4H, FMOC), 5.77 (br d, *J* = 7, 1H, NH), 4.70–4.65 (m, 1H), 4.4–4.39 (m, 2H, FMOC), 4.20 (t, *J* = 7.0, 1H, FMOC), 3.74 (s, 3H), 3.17 (d, *J* = 4.8, 2H); ^13^C-NMR (100 MHz, CDCl_3_) δ 170.9, 155.7, 143.8, 143.7, 141.3, 127.8, 127.1, 125.1, 120.0, 67.3, 53.3, 52.9, 47.1, 41.1; MS (ESI-TOF) calcd. for C_38_H_37_N_2_O_8_S_2_ 713.83898, found 713.3859 [M + H]^+^. Spectra appear in the Supplementary Materials: Figures S29–S34.

### 3.11. Synthesis of Conjugate ***7-B***

**Figure 12:**
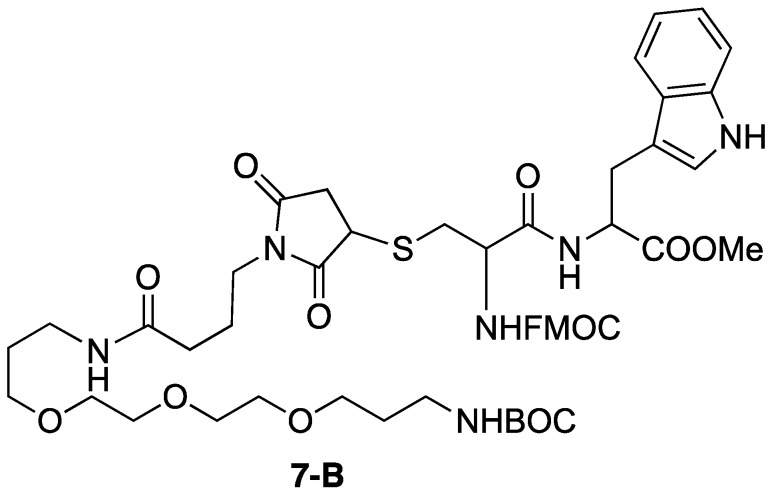
Intermediate **7-B**.

To a solution of **7** (12 mg, 0.025 mmol) in dioxane (1.0 mL), a solution of **B** (18 mg, 0.033) in dichloromethane (1.0 mL) was added at RT. The solution was stirred for 48h at room temperature and evaporated under vacuum. The crude product was purified by column chromatography (DCM = 100 → DCM:MeOH = 95:3) to give **7-B** (Figure 12) as an oil (16.5 mg, 64%). ^1^H-NMR (400 MHz, CDCl_3_): δ 7.75 (t, *J* = 7.6, 2H), 7.58 (d, *J* = 7.1, 2H), 7.52 (t, *J* = 8.5, 1H), 7.42–7.25 (m, 6H), 7.14–7.03 (m, 2H), 4.98–4.84 (m, 2H), 4.69–4.44 (m, 1H), 4.44–4.34 (m, 2H), 4.23–4.17 (m, 1H), 4.04 (br s, 1H), 3.59–3.45 (m, 18H), 3.40–2.80 (m, 9H), 2.47–2.41 (m, 1H), 2.23–2.17 (m, 2H), 2.00–1.86 (m, 2H), 1.79–1.75 (m, 4H), 1.43 (s, 9H) ^13^C-NMR (100 MHz, CDCl_3_) δ 178.1, 177.5, 174.6, 174.4, 172.1, 172.0, 171.9, 169.7, 169.6, 156.1, 143.8, 143.7, 141.3, 136.6, 136.4, 127.8, 127.4, 127.3, 127.1, 125.1, 124.1, 121.8, 121.7, 120.0, 119.96, 119.91, 119.2, 119.1, 118.4, 118.3, 111.5, 108.6, 108.2, 79.0, 70.5, 70.2, 70.1, 69.8, 69.4, 67.2, 67.0, 53.4, 52.9, 52.7, 52.5, 52.4, 47.1, 47.06, 40.8, 39.4, 38.8, 38.4, 38.2, 37.9, 36.7, 35.9, 33.3, 33.2, 28.5, 27.4, 23.4, 23.3; MS (ESI-TOF) calcd for C_53_H_69_N_6_O_13_S 1029.4643, found 1029.6996 [M + H]^+^. Spectra appear in the Supplementary Materials: Figures S35–S38.

### 3.12. Synthesis of Conjugate ***1**-**A_4_***

**Figure 13:**
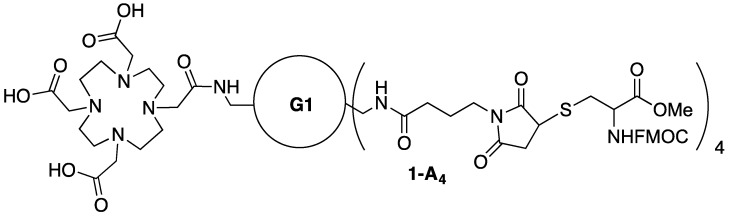
Dendrimers **1**-**A_4_**.

To a solution of **1** (26 mg, 0.009 mmol) in dioxane (1.0 mL) a solution of **A** (25 mg, 0.074) in dichloromethane (1.0 mL) was added at RT. The solution was stirred for 48 h at room temperature and evaporated under vacuum. The crude product was purified by column chromatography (DCM = 100 → DCM:MeOH = 9:1) to give **1**-**A_4_** (Figure 13) as an oil (25 mg, 65%). ^1^H-NMR (400 MHz, CDCl_3_): δ 7.75 (d, *J* = 7.5, 8H, FMOC), 7.61 (t, *J* = 7.2, 8H, FMOC), 7.41–7.29 (m, 16H, FMOC), 4.68 (br s, 4H, p), 4.44–4.40 (m, 8H, FMOC), 4.23 (t, *J* = 7.2 , 4H, FMOC), 3.81–3.70 (m, 4H, n), 3.78 (s, 3H, OCH_3_), 3.69–3.10 (m, 128H, a, c, d, e, f, g, h, j, m, n’, o), 2.48–2.38 (m, 4H, n’), 2.14 (t, *J* = 6.5, 8H, k), 1.94–1.65 (m, 36H, b, i, l); ^13^C-NMR (100 MHz, CDCl_3_) δ 177.0, 176.7, 174.6, 174.4, 171.7, 171.0, 156.0, 143.8, 143.7, 141.3, 127.8, 127.1, 125.2, 11.98, 70.55, 70.46, 70.2, 70.0, 69.7, 69.2, 67.1, 54.2, 53.3, 52.9, 52.8, 47.1, 39.7, 38.7, 38.6, 38.2, 37.7, 36.1, 35.7, 34.4, 33.1, 33.52, 33.46, 29.6, 29.3, 29.0, 23.7, 23.6; MS (ESI-TOF) calcd. for C_203_H_290_N_35_O_56_S_4_ 4241.9804, found 4243.7824 [M + H]^+^. Spectra appear in the Supplementary Materials: Figures S39–S44.

### 3.13. Synthesis of Conjugate ***1**-**B_4_***

**Figure 14:**
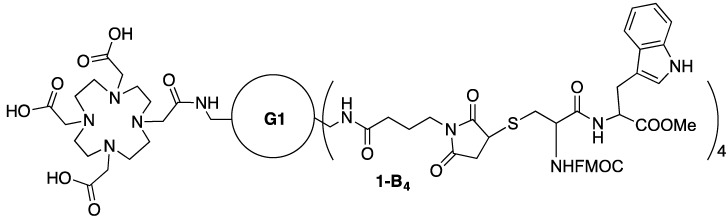
Dendrimer **1**-**B_4_**.

To a solution of **1** (11 mg, 0.0025 mmol) in dioxane (1.0 mL) a solution of **B** (17 mg, 0.031 mmol) in dichloromethane (1.0 mL) was added at RT. The solution was stirred for 48h at room temperature and evaporated under vacuum. The crude product was purified by column chromatography (DCM = 100 → DCM:MeOH = 9:1) to give **1**-**B_4_** (Figure 14) as an oil (10.5 mg, 54%). ^1^H-NMR (400 MHz, CDCl_3_): δ 7.75–7.00 (m, 52H), 4.89–4.01 (m, 20H, p, q, FMOC), 3.46–2.95 (m, 152H, a, c, d, e, f, g, h, j, m, n, o, r, OMe), 2.88-2.81 (m, 4H, n’), 2.37–2.31 (m, 4H, n’), 2.28–2.17 (m, 8H, k), 1.97–1.59 (m, 36H, b, i, l); ^13^C-NMR (100 MHz, CDCl_3_) δ 177.9, 177.5, 174.6, 174.5, 172.0, 172.0, 169.9, 156.2, 143.8, 143.7, 141.3, 136.6, 136.4, 127.8, 127.4, 127.1, 125.2, 124.1, 121.6, 120.0, 119.94, 119.1, 118.3, 118.2, 111.6, 108.6, 108.3, 70.4, 70.1, 70.0, 69.8, 69.6,69.1, 67.2, 67.1, 55.1, 53.5, 53.3, 53.0, 52.8, 52.43, 52.38, 47.1, 40.6, 39.4, 38.7, 38.1, 37.9, 37.7, 36.3, 35.9, 33.3, 33.2, 29.7, 29.3, 29.0, 27.4, 23.5, 22.7; MS (ESI-TOF) calcd. for C_247_H_330_N_42_O_60_S_4_ 4986.2976, found 4989.1225 [M + H]^+^. Spectra appear in the Supplementary Materials: Figures S45–S49.

### 3.14. Synthesis of Conjugate ***1**-**C_4_***

**Figure 15:**
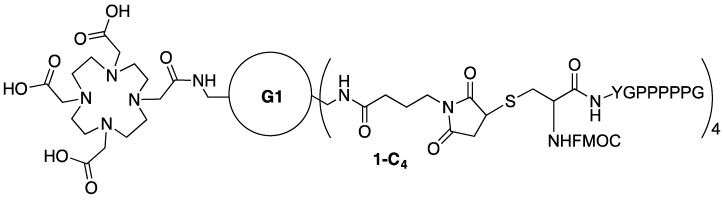
Dendrimer **1**-**C_4_**.

To a solution of maleimide **1** (9.8 mg, 0.0034 mmol) in dioxane (0.5 mL), a solution of CYGPPPPPG **C** (25 mg, 0.0279 mmol) in water (0.5 mL) was added, followed by addition of DIPEA (10 µL, 0.056 mmol) at RT. The solution was stirred for 18h at room temperature and then evaporated under vacuum. The crude product was diluted with deionized water (1.5 mL) and excess amount of peptide was removed from the dendron by centrifugal filtration (3000 MW cut off, 4000 rpm). The residue was washed with deionized water (2 mL) five times and lyophilized to give **1**-**C_4_** (Figure 15) as a solid (14 mg, 63%). ^1^H-NMR (400 MHz, D_2_O): δ 7.19–7.06 (m, 8H, x), 6.89–6.73 (m, 8H, y), 4.64–4.57 (m, 20H, t), 4.37–4.34 (m, 4H, q), 4.26–4.22 (m, 4H, p), 3.99–3.86 (m, 8H, s), 3.85–3.68 (m, 32H, n, s, w), 3.65–2.81 (m, 156H, a, c, d, e, f, g, h, m, n’, o, r, w), 2.59–2.51 (m, 4H, n’), 2.31-2.15 (m, 28H, k, u), 2.05–1.65 (m, 96H, b, i, l, u, v); ^13^C-NMR (100 MHz, D_2_O) δ 178.8, 178.7, 176.8, 174.8, 174.7, 174.1, 173.2, 173.1, 171.7, 171.4, 170.85, 170.7, 169.1, 167.1, 166.9, 162.2, 161.9, 154.0, 153.9, 129.8, 129.75, 127.0, 126.8, 117.0, 114.6, 114.1, 71.3, 68.9, 68.65, 67.8, 67.6, 67.5, 65.8, 61.7, 60.2, 59.7, 58.5, 57.8, 55.7, 55.0, 54.7, 54.6, 51.7, 50.9, 50.0, 47.5, 47.4, 46.9, 46.8, 46.2, 42.0, 41.9, 40.8, 37.8, 37.0, 35.7, 35.4, 34.8, 32.1, 28.5, 27.6, 27.3, 27.1, 23.9, 23.5, 22.2; MS (ESI-TOF) calcd. for C_291_H_442_N_67_O_84_S_4_ 6347.1257, found 6295.1574 [M + H]^+^. Spectra appear in the Supplementary Materials: Figures S50–S55.

### 3.15. Synthesis of Conjugate ***1**-**D_4_***

**Figure 16:**
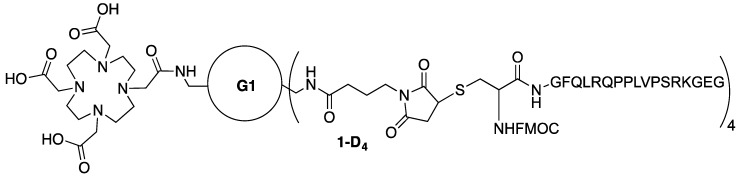
Dendrimer **1**-**D_4_**.

To a solution of **1** (9.1 mg, 0.0032 mmol) in water (0.5 mL), a solution of CGFQLRQPPLVPSRKGEG, **D** (51 mg, 0.026 mmol) in water (0.5 mL) was added followed by addition of 0.1 N NaOH (adjust pH ~ 7–8). The solution was stirred for 18 h at RT before diluting with deionized water (1.0 mL). Excess peptide was removed by centrifugal filtration (3000 MW cut off, 4000 rpm). The residue was washed with deionized water (2 mL) five times and lyophilized to give **1-D_4_** (Figure 16) as a solid (19.7 mg, 57%). ^1^H-NMR (400 MHz, D_2_O): δ 7.25–7.13 (m, 20H, 2), 4.61–4.49 (m, 8H, r, x), 4.39–4.22 (m, 52H, p, s(2), t(2), u(2), v(3), w, y, z), 3.95–.2.90 (m, 224H, a, c, d, e, f, g, h, j, m, n, n’, o, q(3), 1, 10(2), 11(3), 16, 20), 2.58–2.52 (m, 4H, n’), 2.33–2.13 (m, 44H, k, 4(2), 13(3), 22), 2.13–1.34 (m, 168H, b, i, l, 3(2), 5(2), 6(2), 8(2), 9(2), 12(3), 13(3), 14, 17, 18, 19, 21), 0.92–0.78 (m, 72H, 7(2), 15) ^13^C-NMR (100 MHz, D_2_O) δ 181.0, 179.4, 179.2, 177.7, 176.2, 174.8, 174.3, 174.1, 173.8, 173.3, 173.2, 173.0, 172.4, 171.8, 171.6, 171.2, 170.7, 170.4, 169.8, 156.7, 136.1, 129.2, 128.7, 127.2, 69.7, 69.4, 68.5, 68.4, 61.0, 60. 5, 60.0, 58.8, 56.8, 56.4, 55.8, 55.5, 55.0, 53.9, 53.5, 53.2, 53.0, 52.7, 52.5, 50.8, 48.3, 47.9, 47.7, 43.5, 42.5, 40.5, 39.7, 39.1, 38.5, 37.7, 37.1, 36.5, 33.4, 32.9, 31.0, 30.7, 30.2, 30.1, 29.5, 29.3, 28.6, 28.4, 28.2, 27.8, 27.0, 26.2, 24.7, 24.4, 23.0, 22.2, 22.0, 21.9, 21.1, 21.0, 18.3, 17.6; MS (MALDI-TOF) calcd. for C_471_H_777_N_139_O_136_S_4_ 10684., found 10662. Spectra appear in the Supplementary Materials: Figures S56–S60.

## 4. Conclusions

In conclusion, **1** represents a valuable platform for the pursuit of theranostic applications. Mass spectrometry provides evidence that, upon metallation with gadolinium, **1**-**Gd** can be reacted with **A** to yield **1**-**A_4_**-**Gd**. This construct and related conjugates will be described in due course. While poly(maleimides) are commercially available (Toronto Research Chemicals), the cost and lack of a chelating-group for imaging applications represent limitations overcome by **1**. Both the synthetic and conjugation chemistry described here are straightforward and can be affected with a minimum of effort. However, the extent to which these reactions proceed is dependent on the size and composition of the thiol. Tetravalent displays of large peptides have proven difficult to push to completion. Critically, conjugation can be accomplished in a range of solvents—from organic solvents to aqueous solutions—to incorporate a range of thiol-containing species. The value of this conjugation strategy—maleimide and thiol—over alternatives has been reported: Wangler *et al.* showed that thiol-maleimide couplings proceed more effectively than oxime formation or copper-catalyzed cycloadditions [26]. Finally, we note that, when the flexible dendritic domains are fully extended, **1** places ligands in the corners of a rectangular array measuring ~2.5 nm by ~4.2 nm.

## Supplementary Materials

The following are available online at: http://www.mdpi.com/1420-3049/21/3/335/s1; ESI-TOF MS, ^1^H-NMR, ^13^C-NMR and Stability test.
