# Supplementary material for: Functionalization of a Triazine Dendrimer Presenting Four Maleimides on the Periphery and a DOTA Group at the Core
Source: Molecules. 2016 Mar 10;21(3):335. doi: 10.3390/molecules21030335 (PMC6273729; doi:10.3390/molecules21030335)
Supplement: Supplementary file 1 [file molecules-21-00335-s001.pdf]

Supplementary Materials: Functionalization of a Triazine Dendrimer Presenting Four Maleimides on the Periphery and a DOTA Group at the Core

Changsuk Lee, Kun Ji and Eric E. Simanek

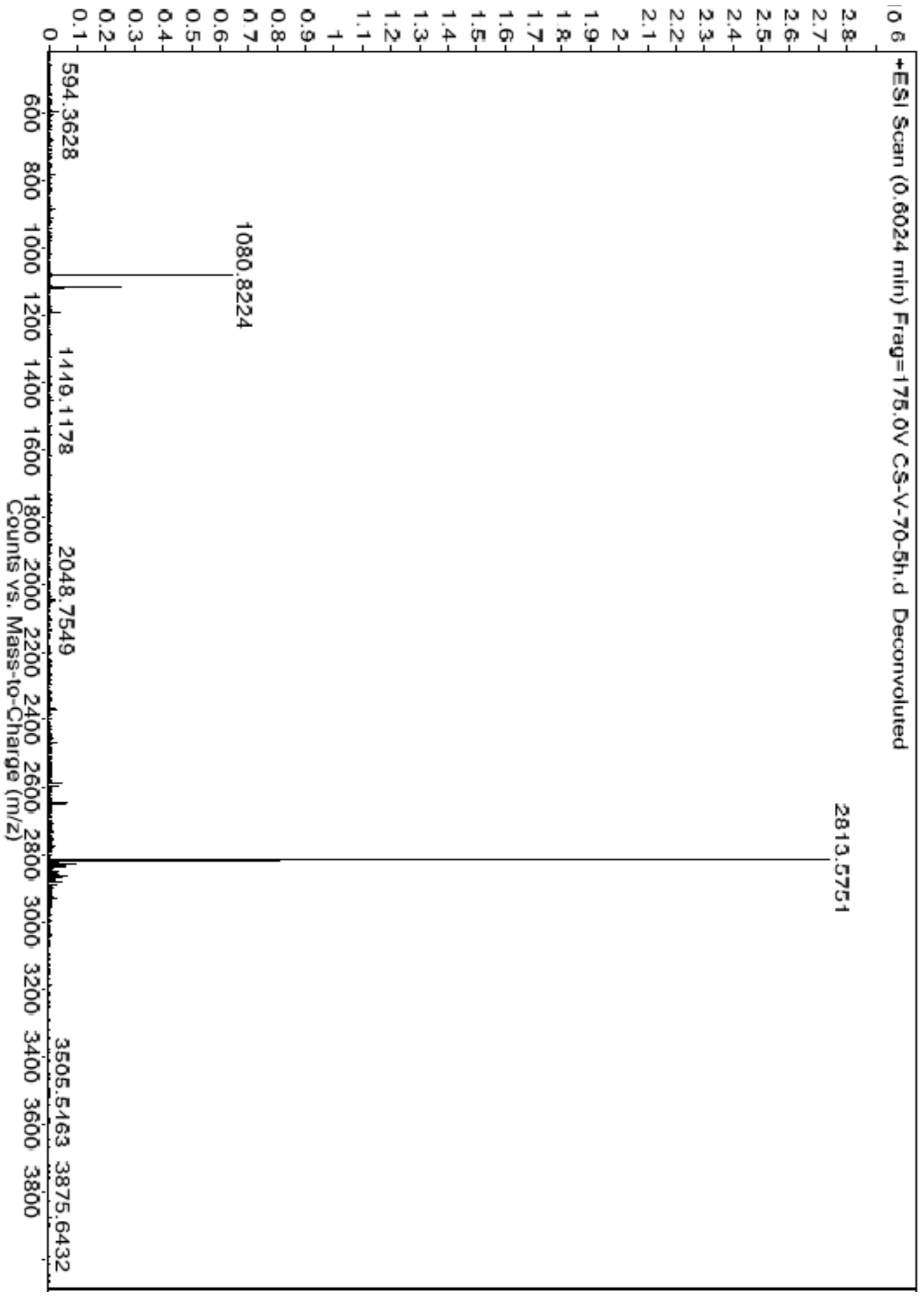

Figure S1. ESI-TOF MS of **1**. The line at *m*/*z* 1080 is not assigned.

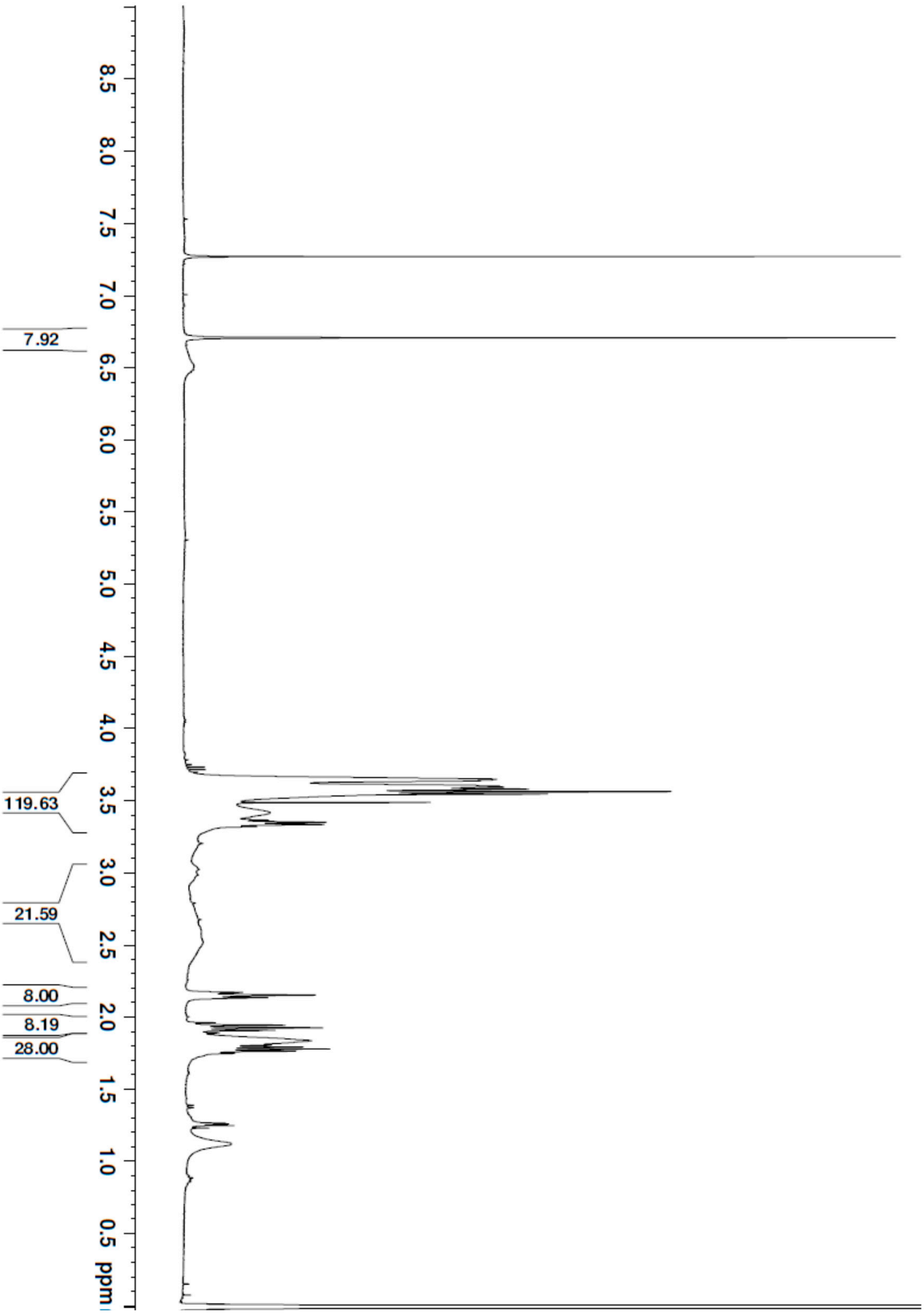

Figure S2. ^{1}H-NMR (400 MHz, $CDCl_3$) of **1**.

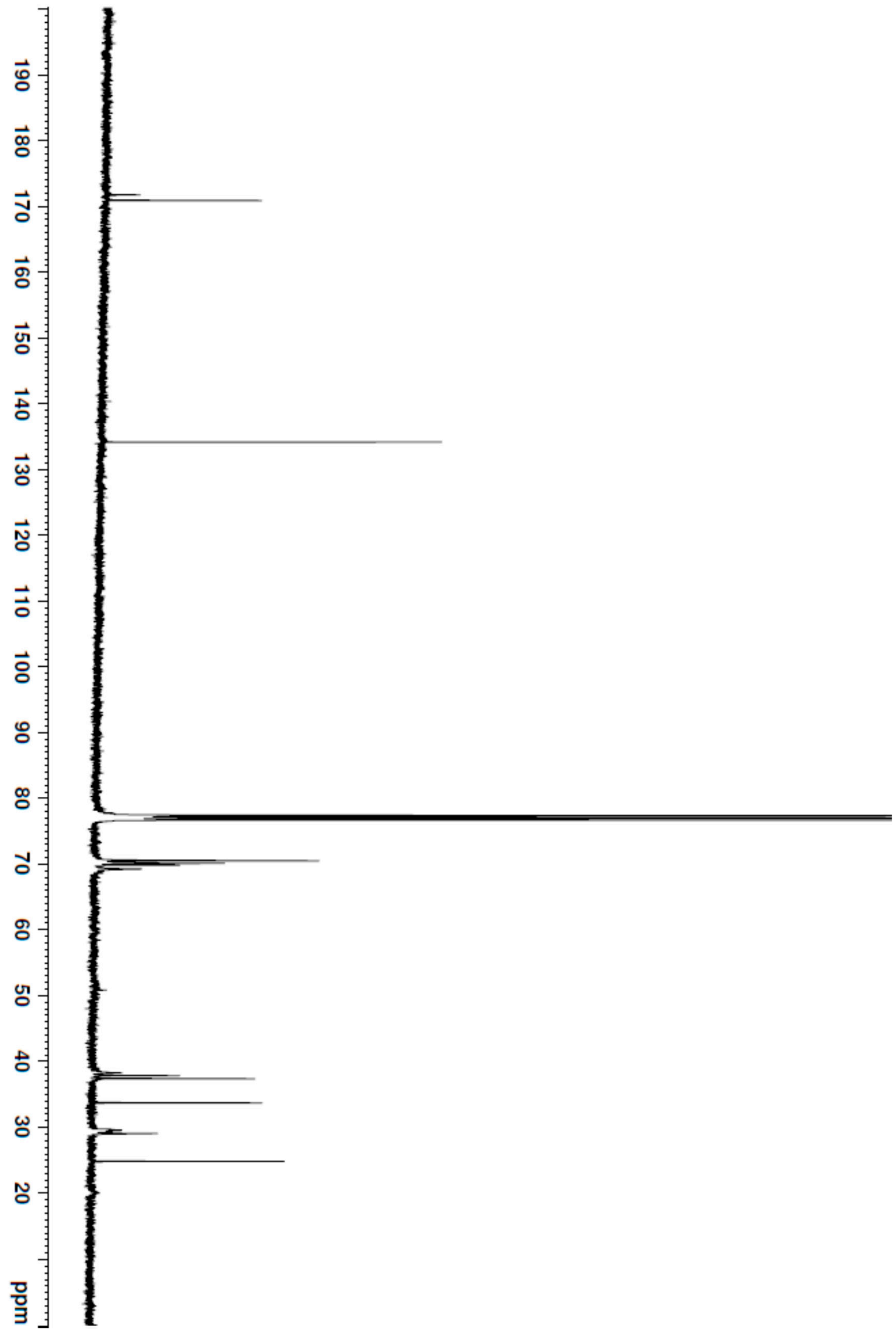

Figure S3. ^{13}C-NMR (400 MHz, $CDCl_3$) of **1**.

Panel 1: **1**

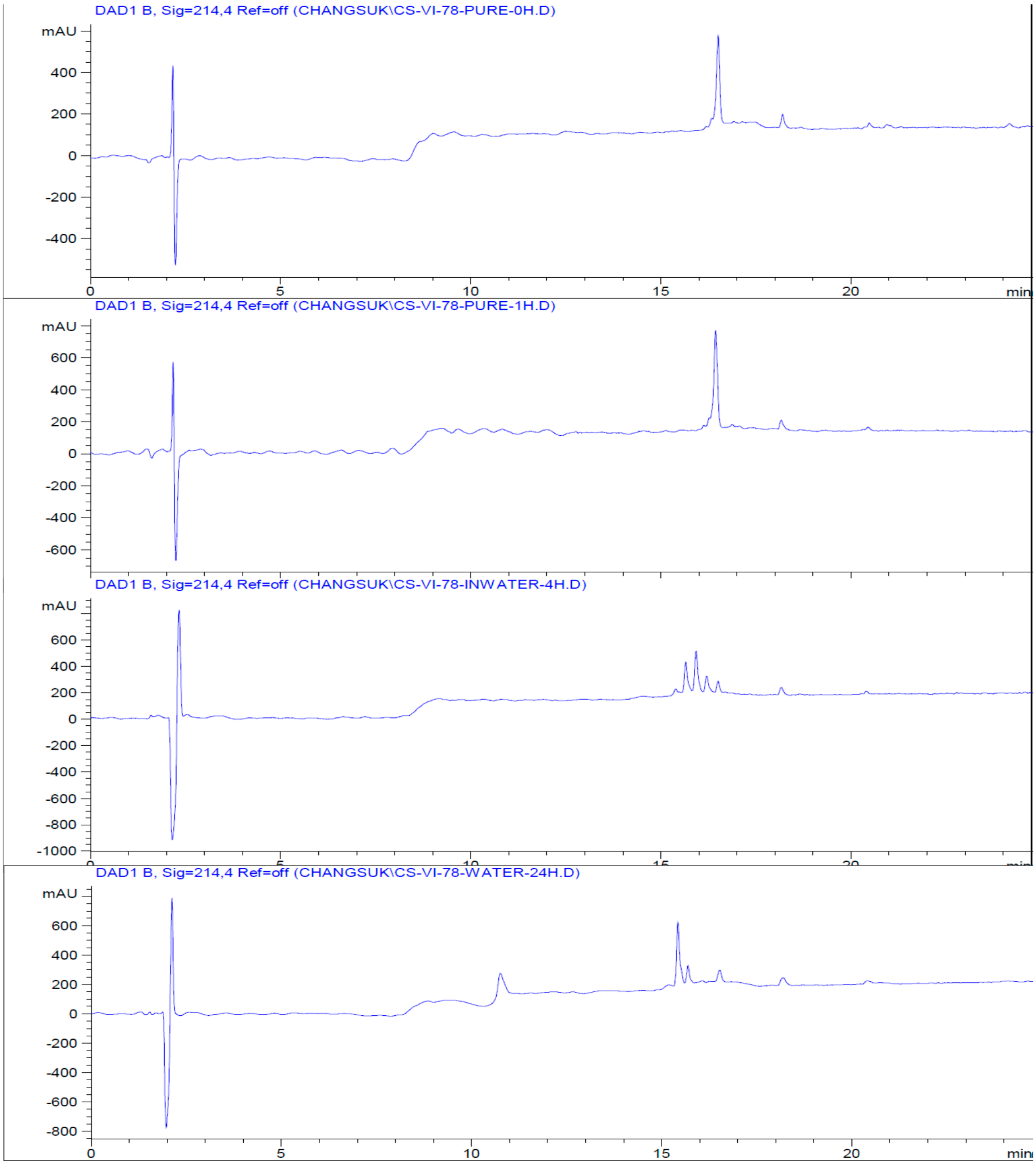

Figure S4. Stability test of **1** in water (pH 7.5) with monitoring by HPLC. Panels are **1**, **1** after 1h, **1** after 4h, **1** after 24h. The mobile phase consisted of water/acetonitrile (A/B, HPLC grade, 0.1% (*w*/*v*) trifluoroacetic acid) at a flow rate of 0.8 mL/min. The elution gradient was 10% MeCN for 5 min, ramp to 90% MeCN in 30 min, and ramp down to 10% MeCN in 15 min. The sample volume injected 5 µL at a concentration of 0.1 mg/mLwith HPLC-grade MeCN, and eluted sample was detected at 214 nm.

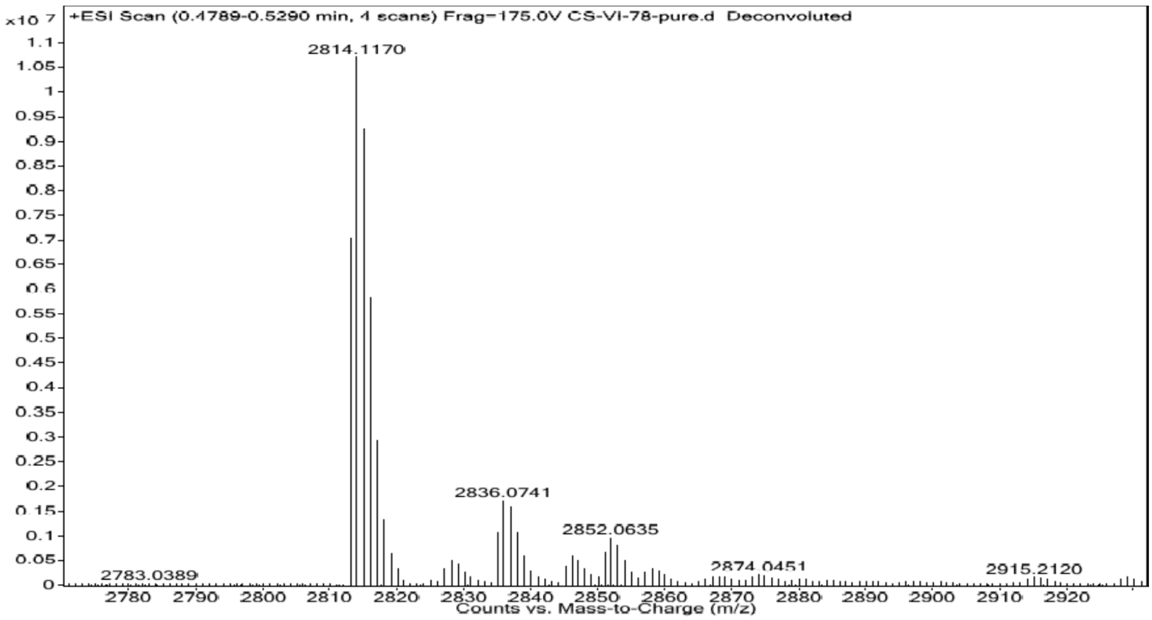

(**A**)

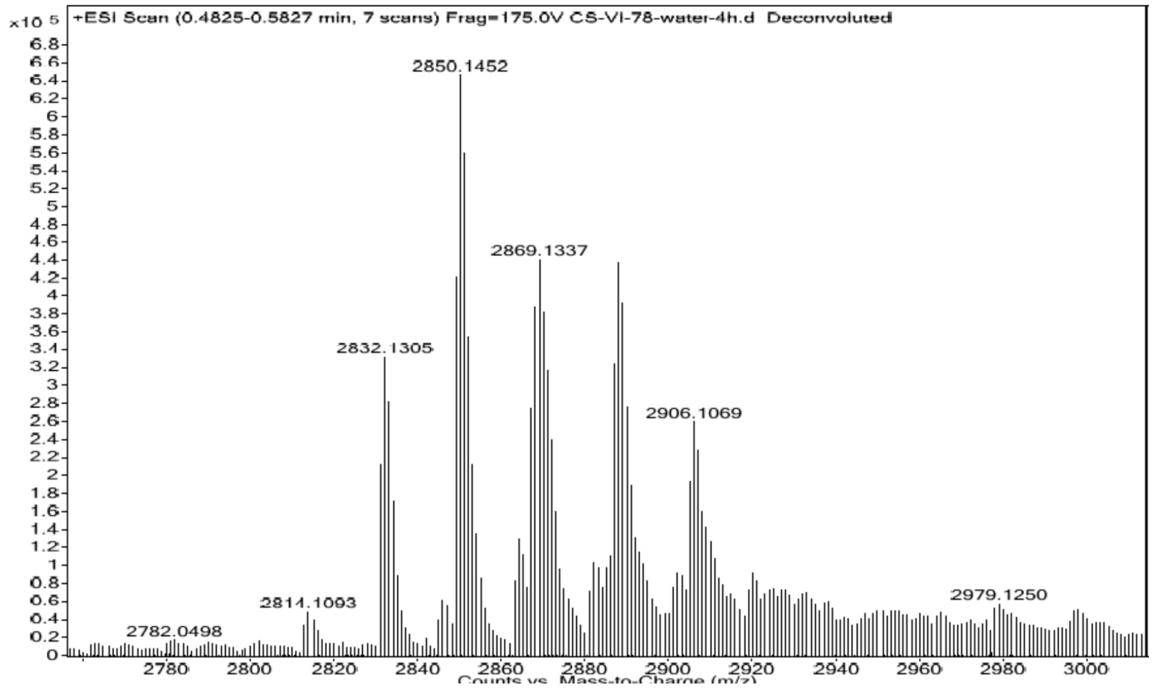

(**B**)

Figure S5. *Cont.*

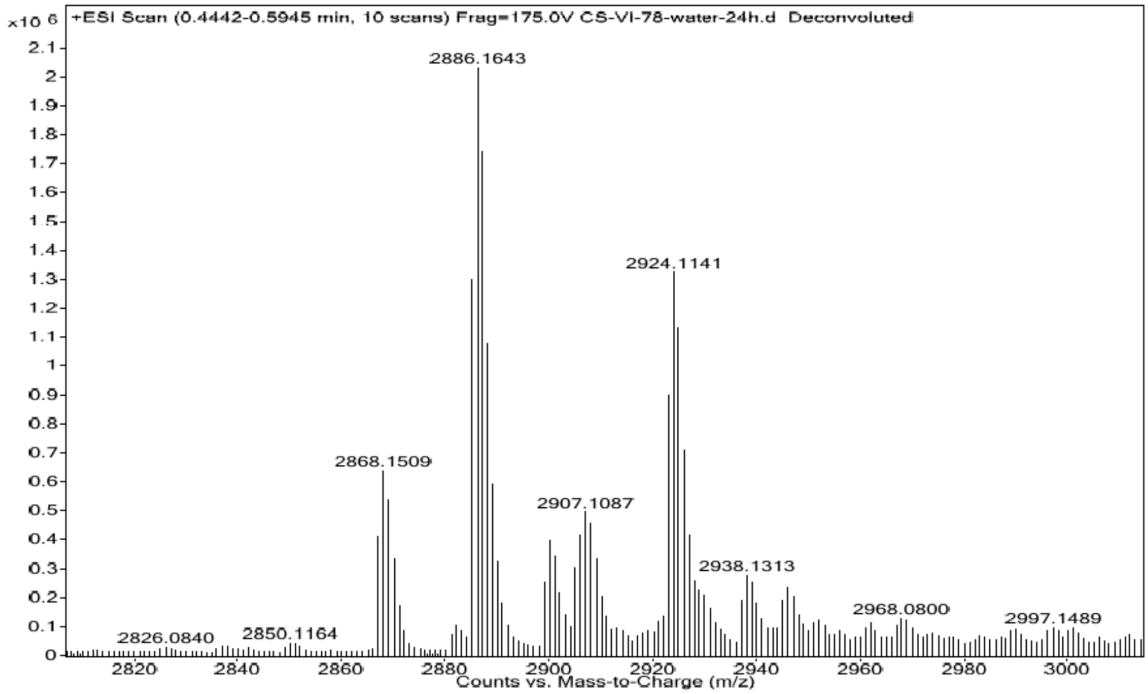

(**C**)

Figure S5. Stability test of **1** in water (pH 7.5) with monitoring by ESI-TOF MS. (**A**) Panel 1: After 1 h; (**B**) Panel 2: After 4 h; (**C**) Panel 3: After 24 h

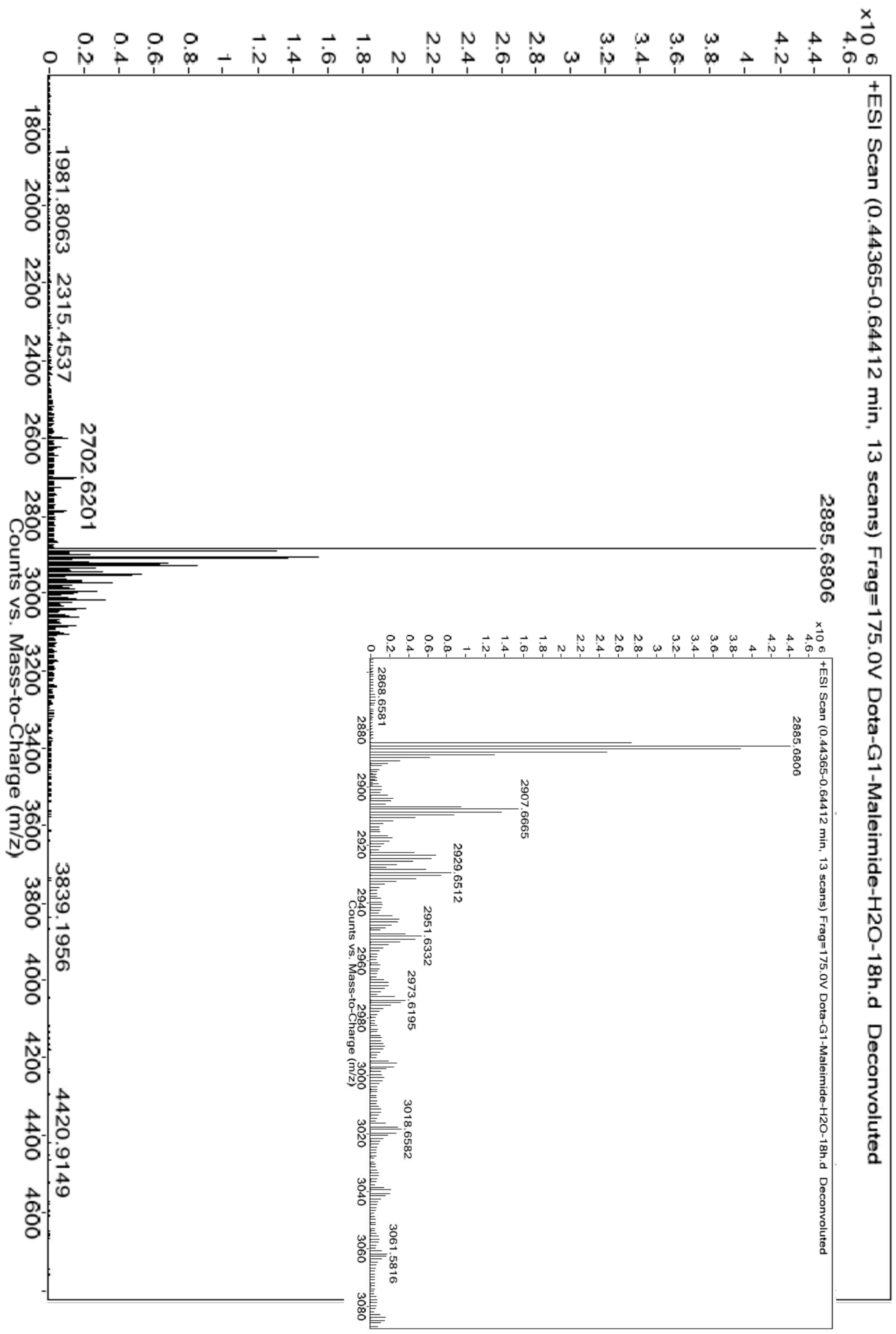

Figure S6. ESI-TOF MS of hydrolysis product of **1**. Inset shows the Na^{+} adducts.

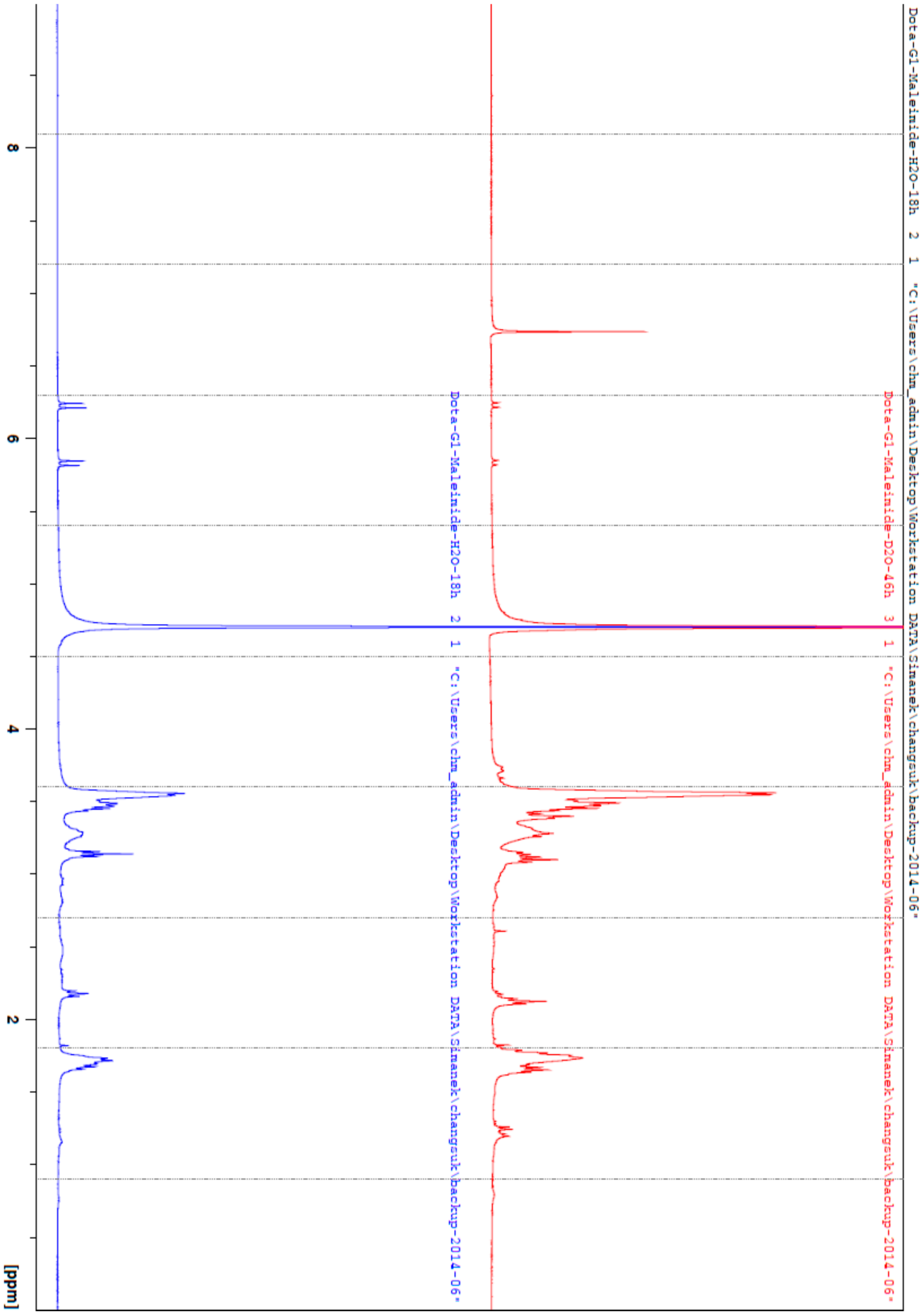

Figure S7. Comparative ^{1}H-NMR of the hydrolysis product. Panels are **1** after 48 h in D_2O, pH 7 (top red), and at pH 7–8, 18 h then lyophilized (bottom blue).

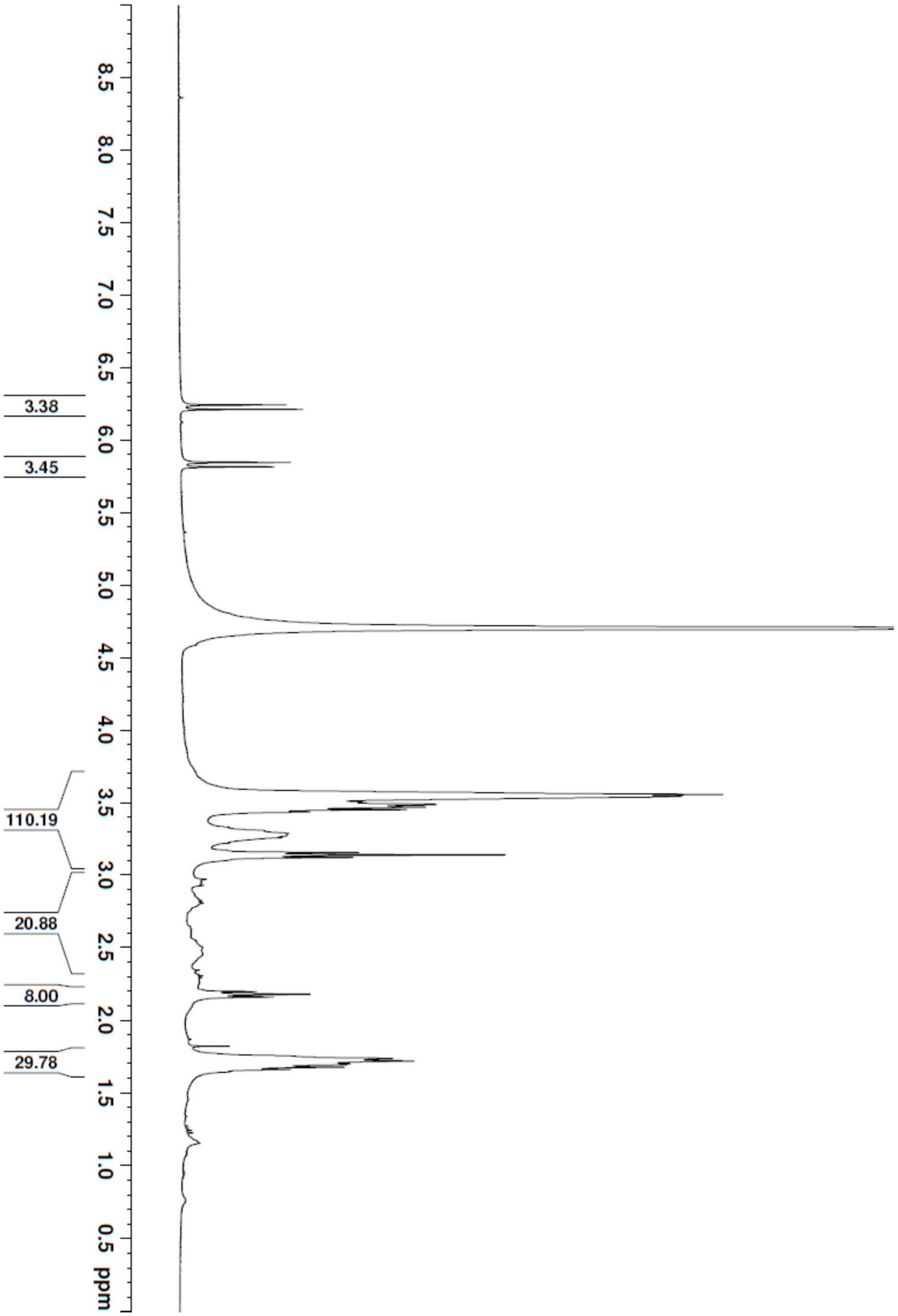

Figure S8. ^{1}H-NMR of the hydrolysis product (400 MHz, D_2O).

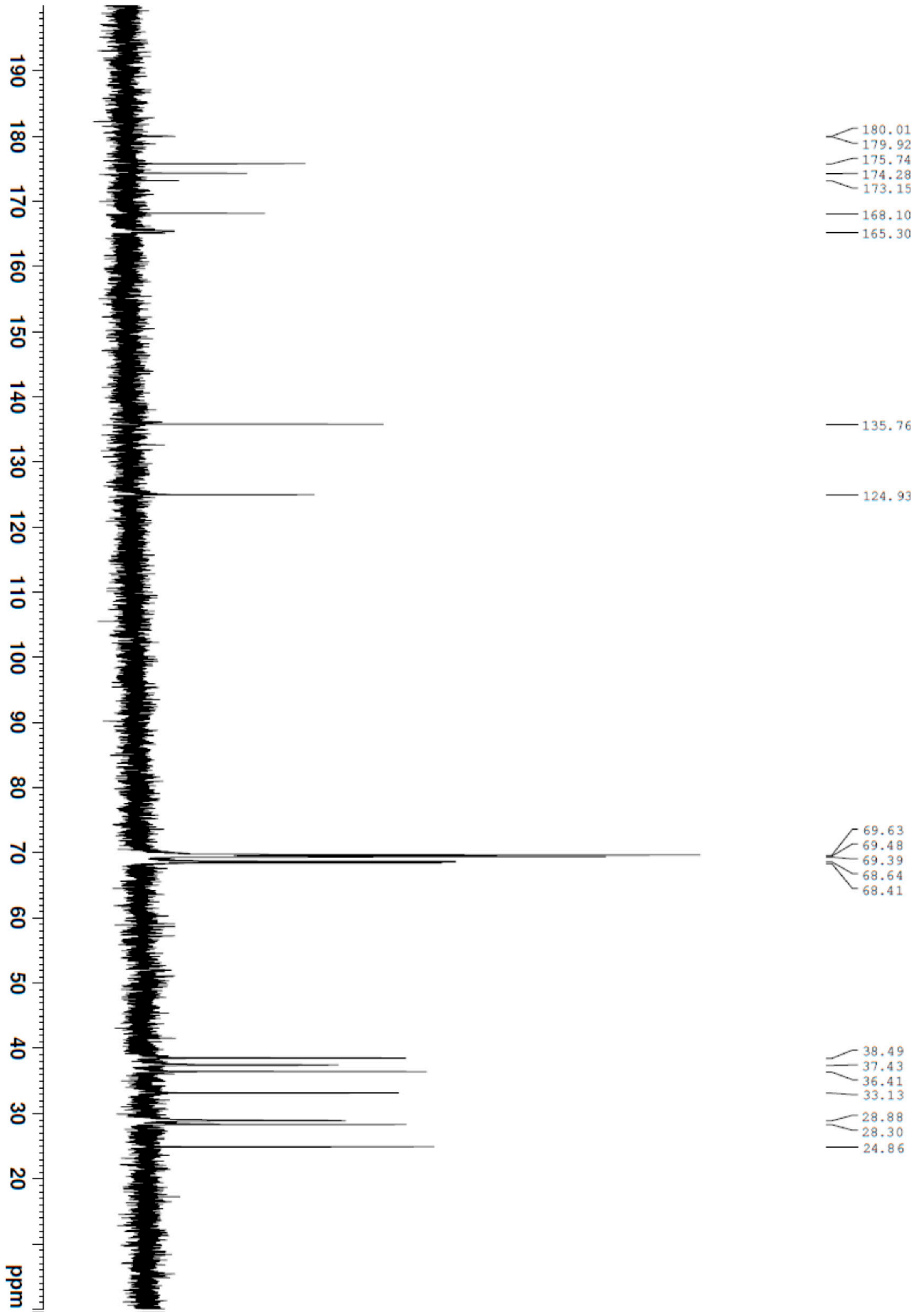

Figure S9. ^{13}C-NMR of the hydrolysis product (400 MHz, D_2O).

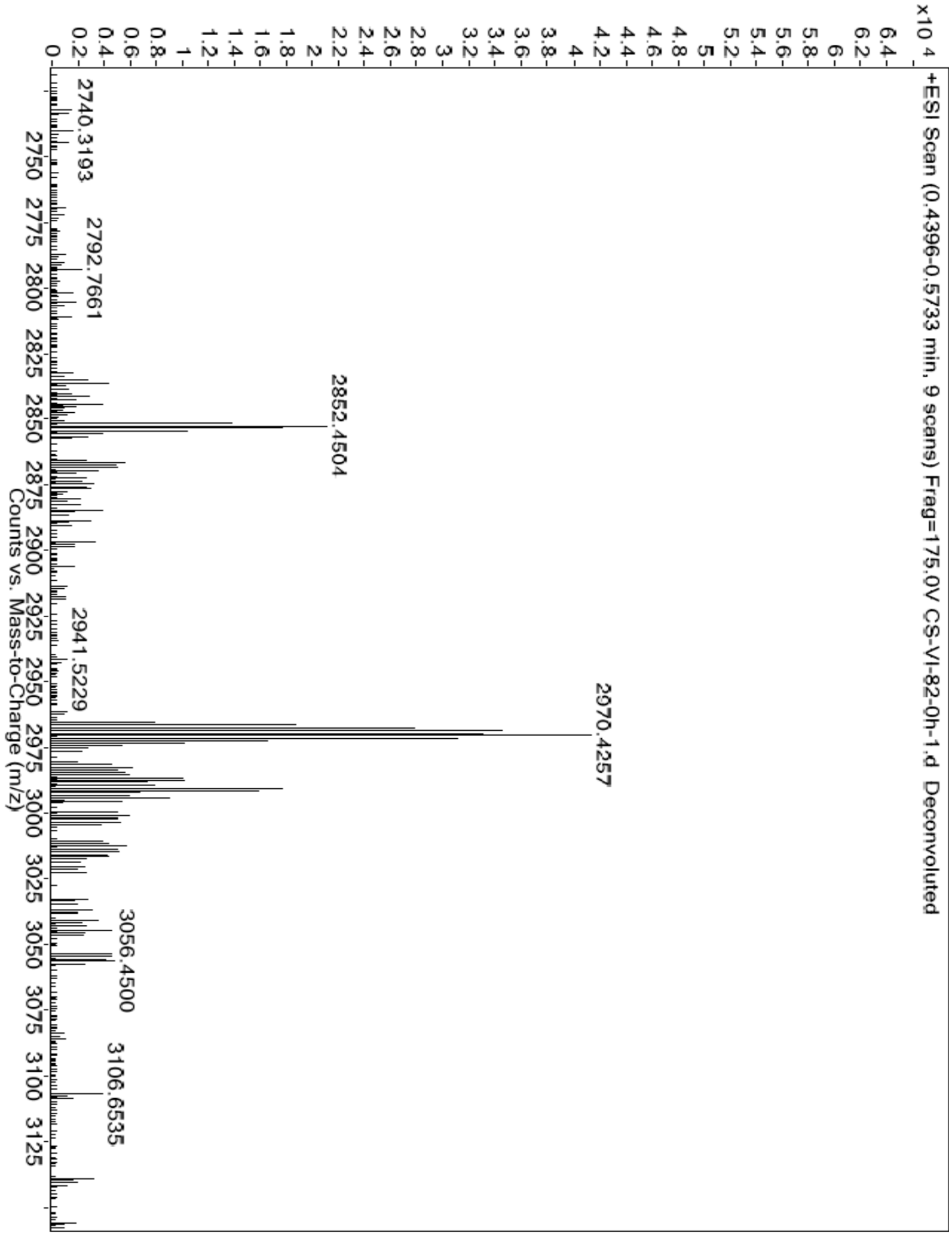

Figure S10. ESI-TOF MS analysis of formation of the Gd adduct of **1**. MS (ESI-TOF) calcd. for $C_{127}H_{213}GdN_{31}O_{40}$ 2970.4827, found 2970.4257 $[M + Gd]^{+}$.

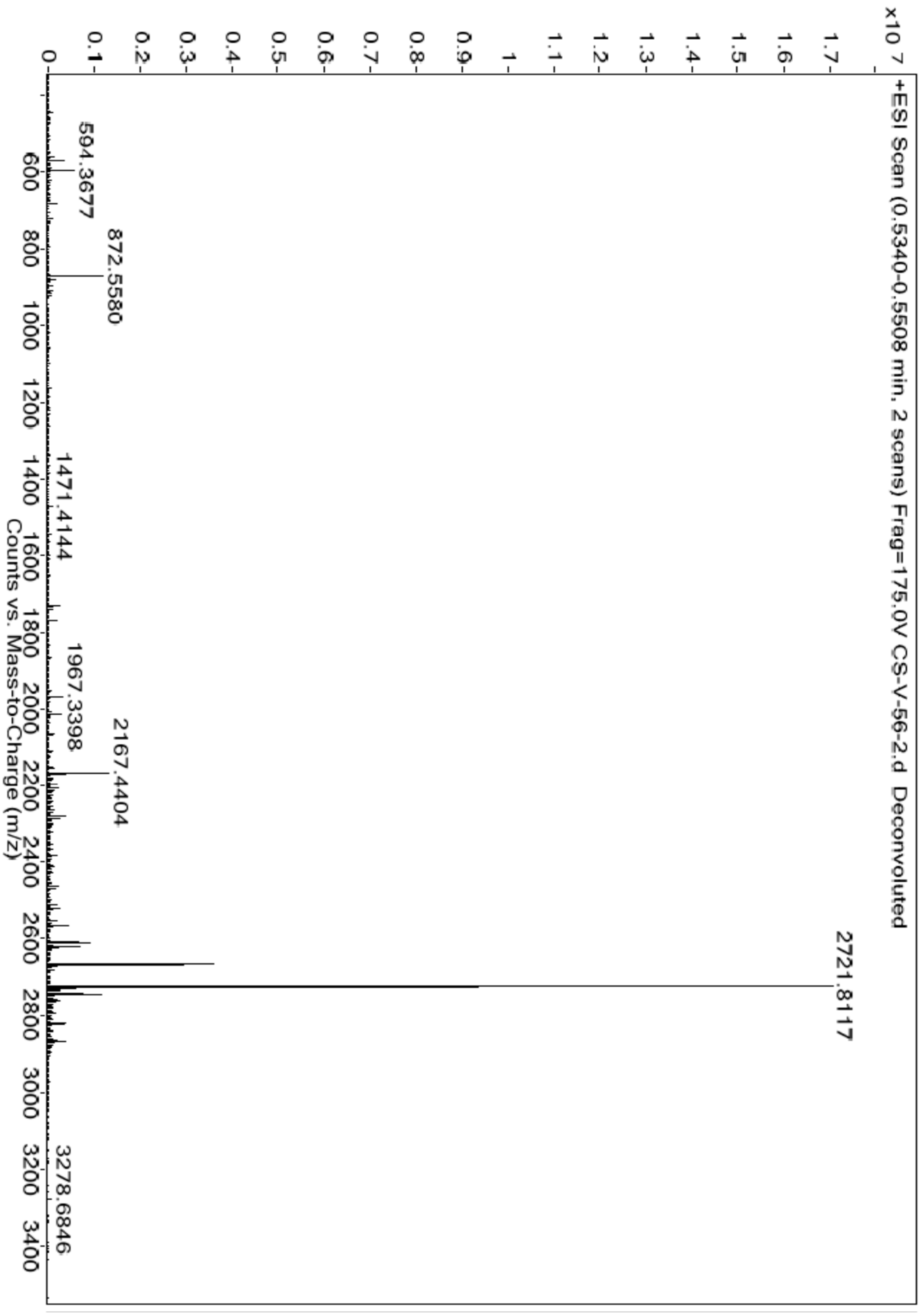

Figure S11. ESI-TOF MS of **4**. Loss of BOC groups are observed. The line at 2167 is not identified.

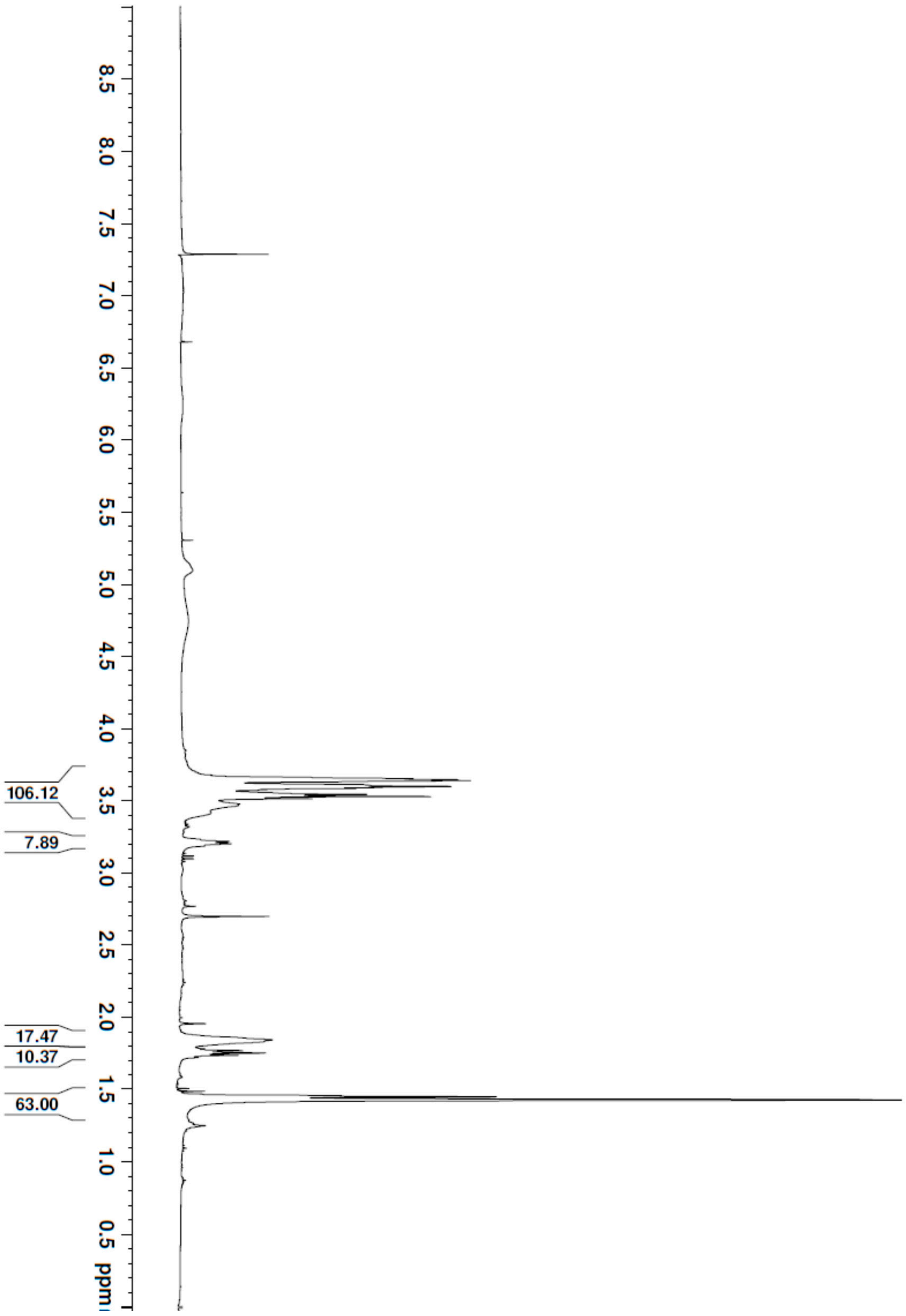

Figure S12. ^{1}H-NMR of **4** (400 MHz, $CDCl_3$).

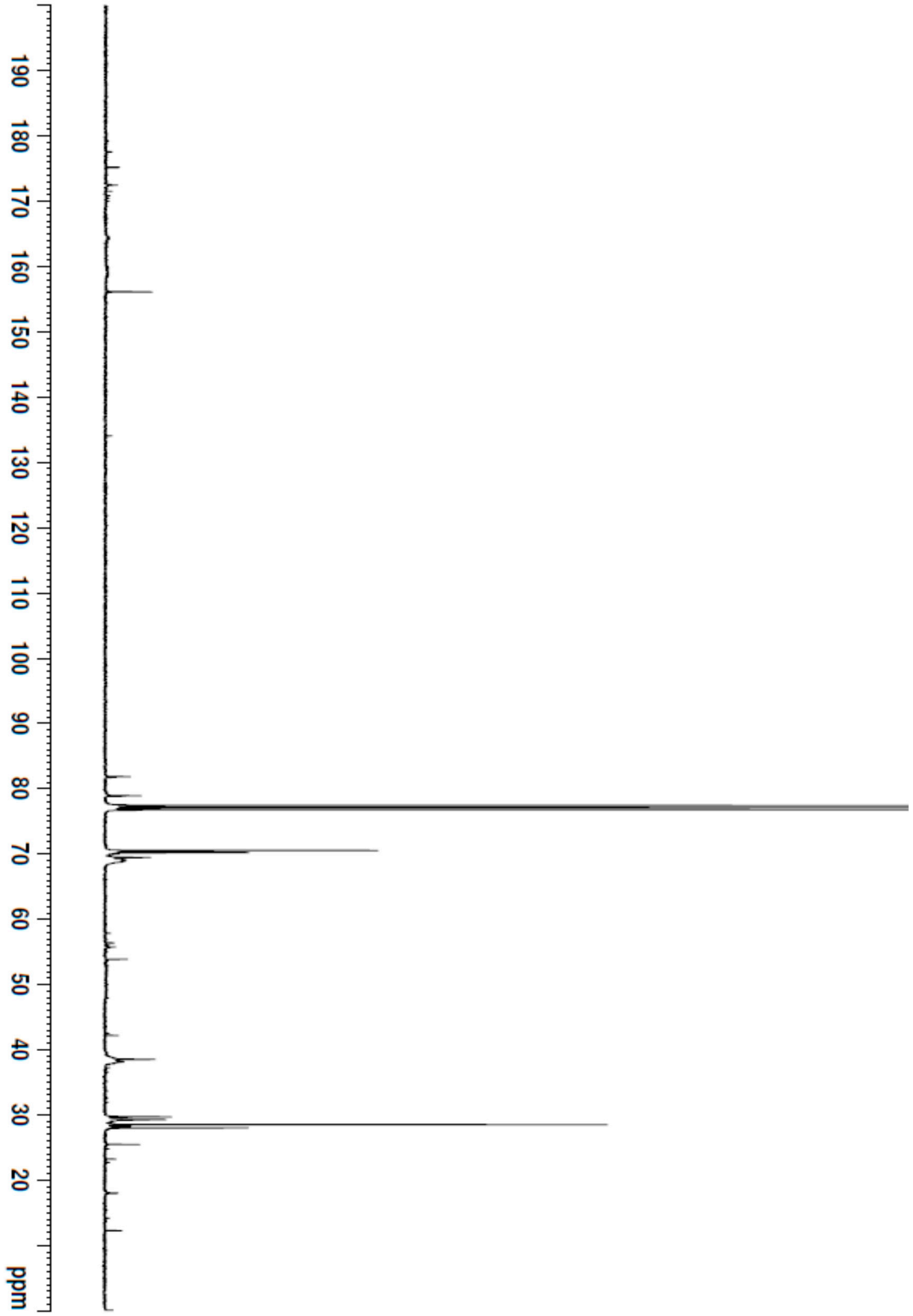

Figure S13. ^{13}C NMR of **4** (100 MHz, $CDCl_3$).

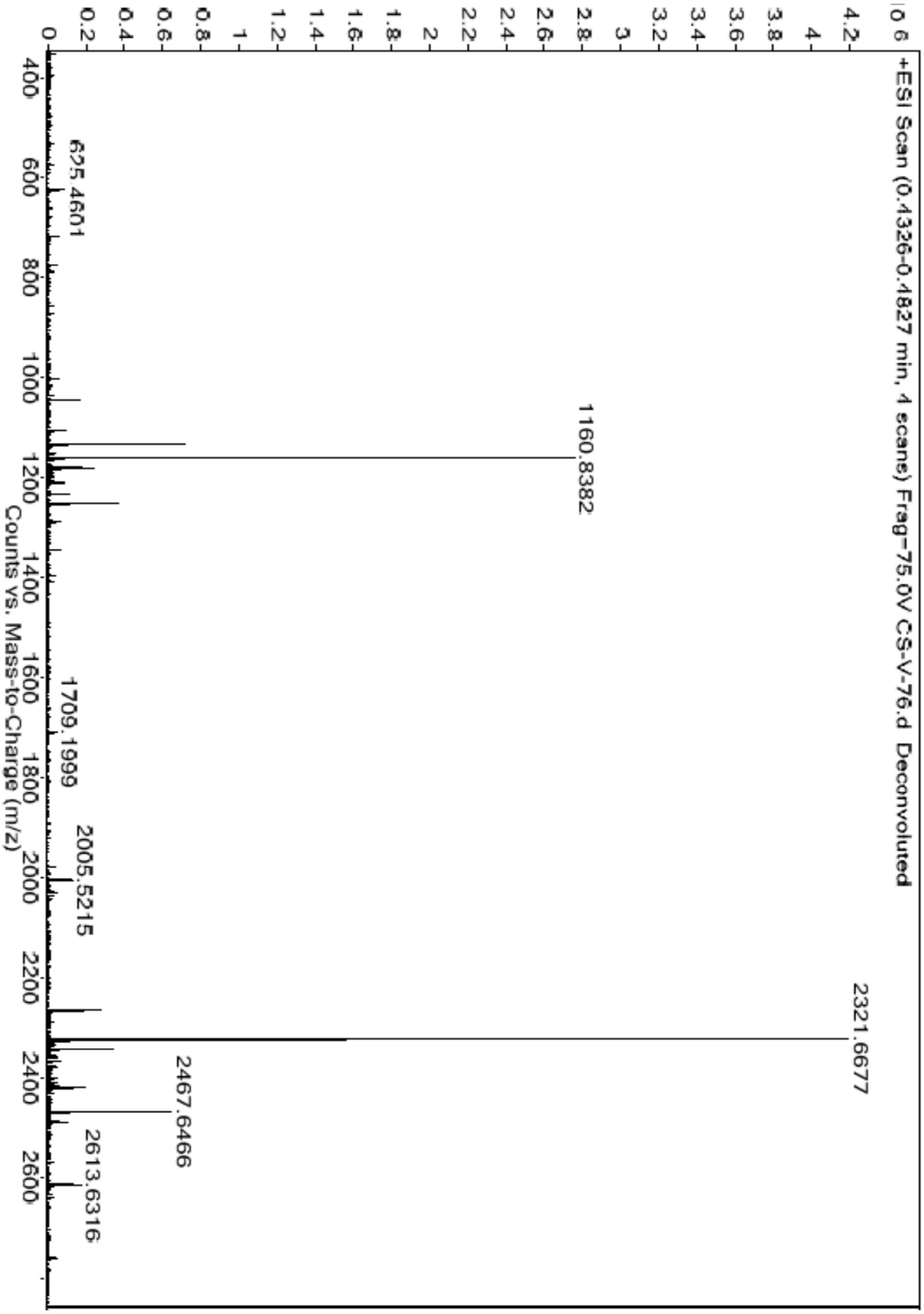

Figure S14. ESI-TOF MS of **5**. The line at *m*/*z* 1160 is the doubly charged species. The lines at *m*/*z* 2467 and 2613 are not identified.

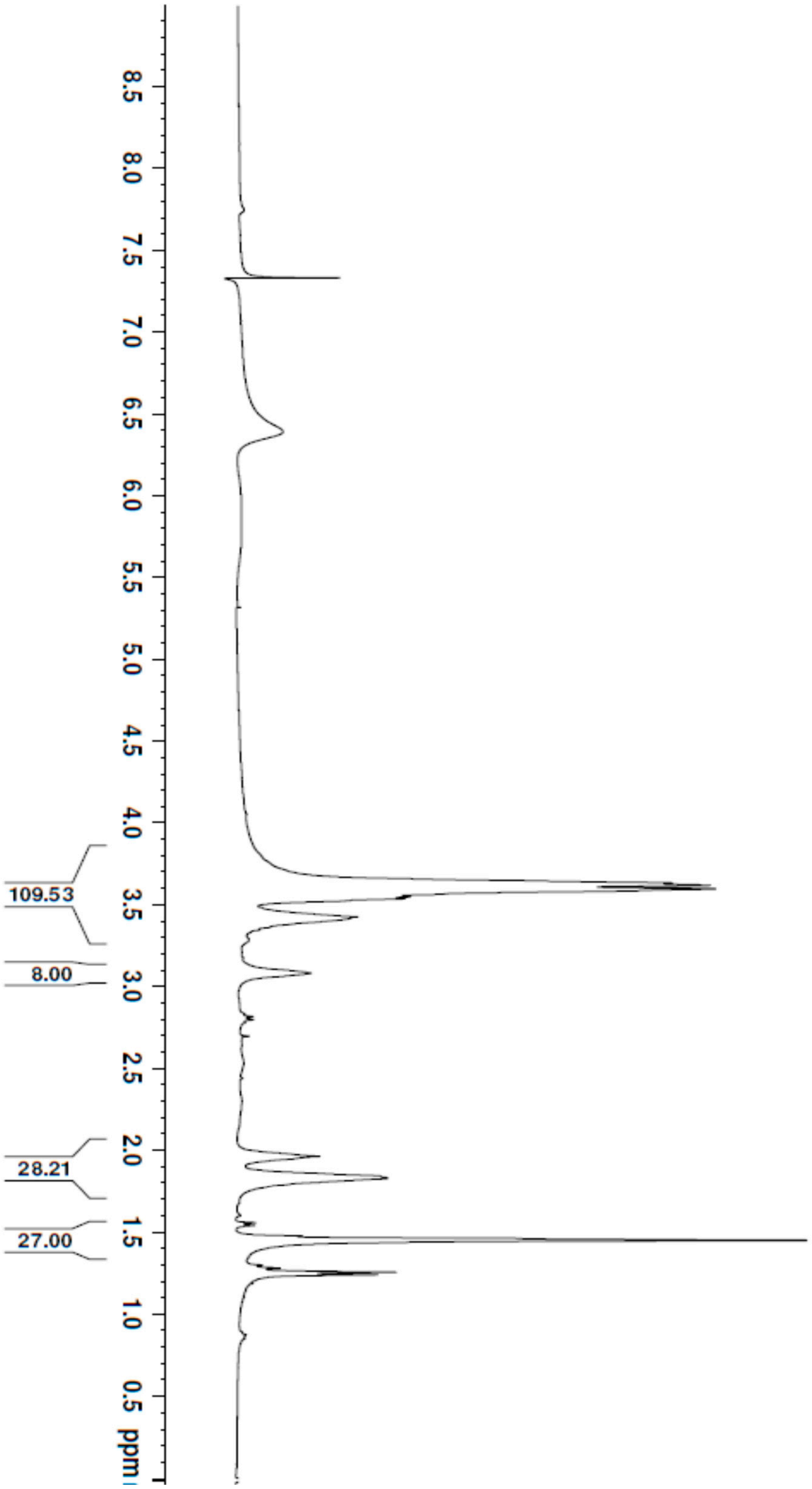

Figure S15. ^{1}H-NMR of **5** (400 MHz, $CDCl_3$).

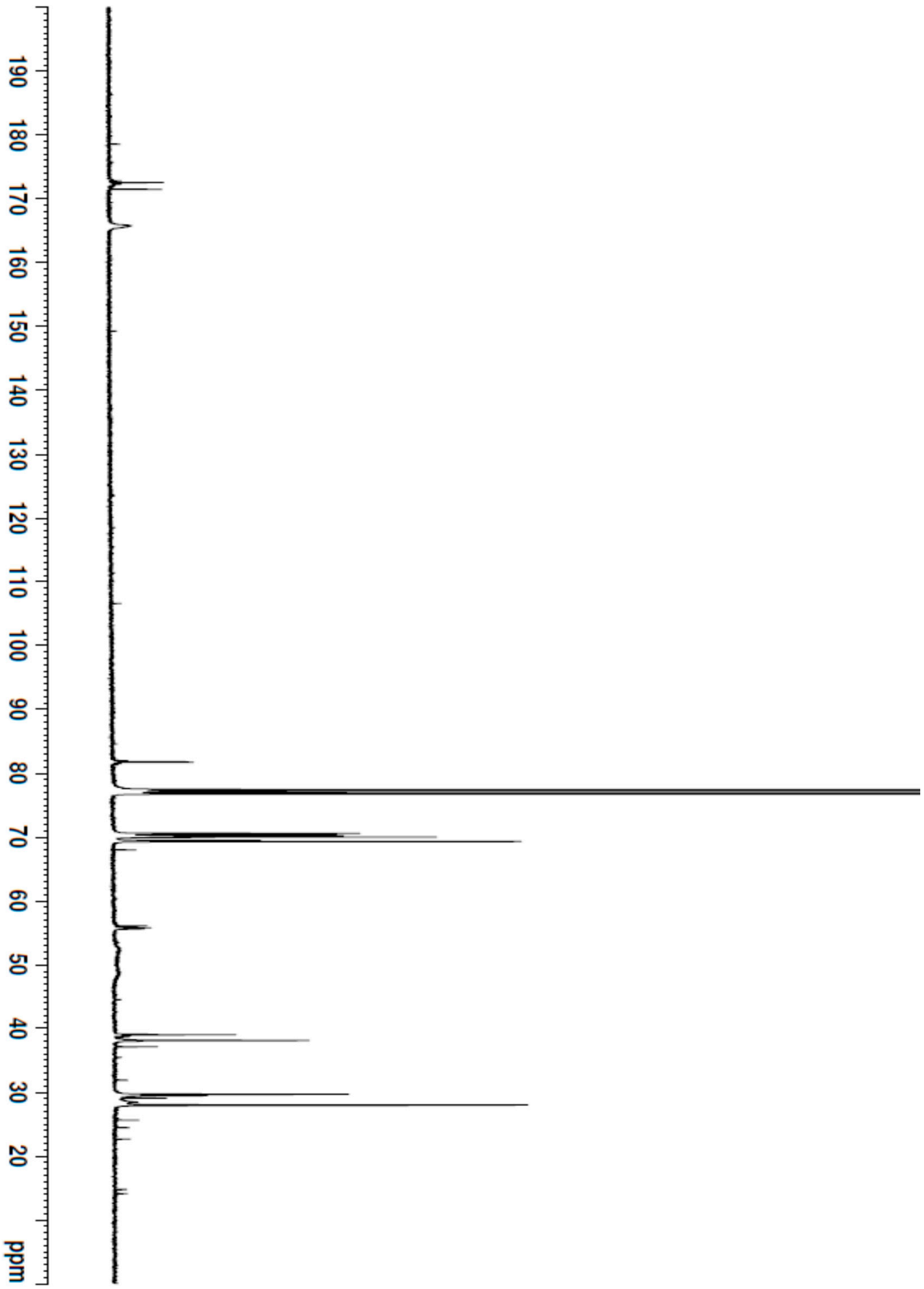

Figure S16. ^{13}C NMR of **5** (100 MHz, $CDCl_3$)

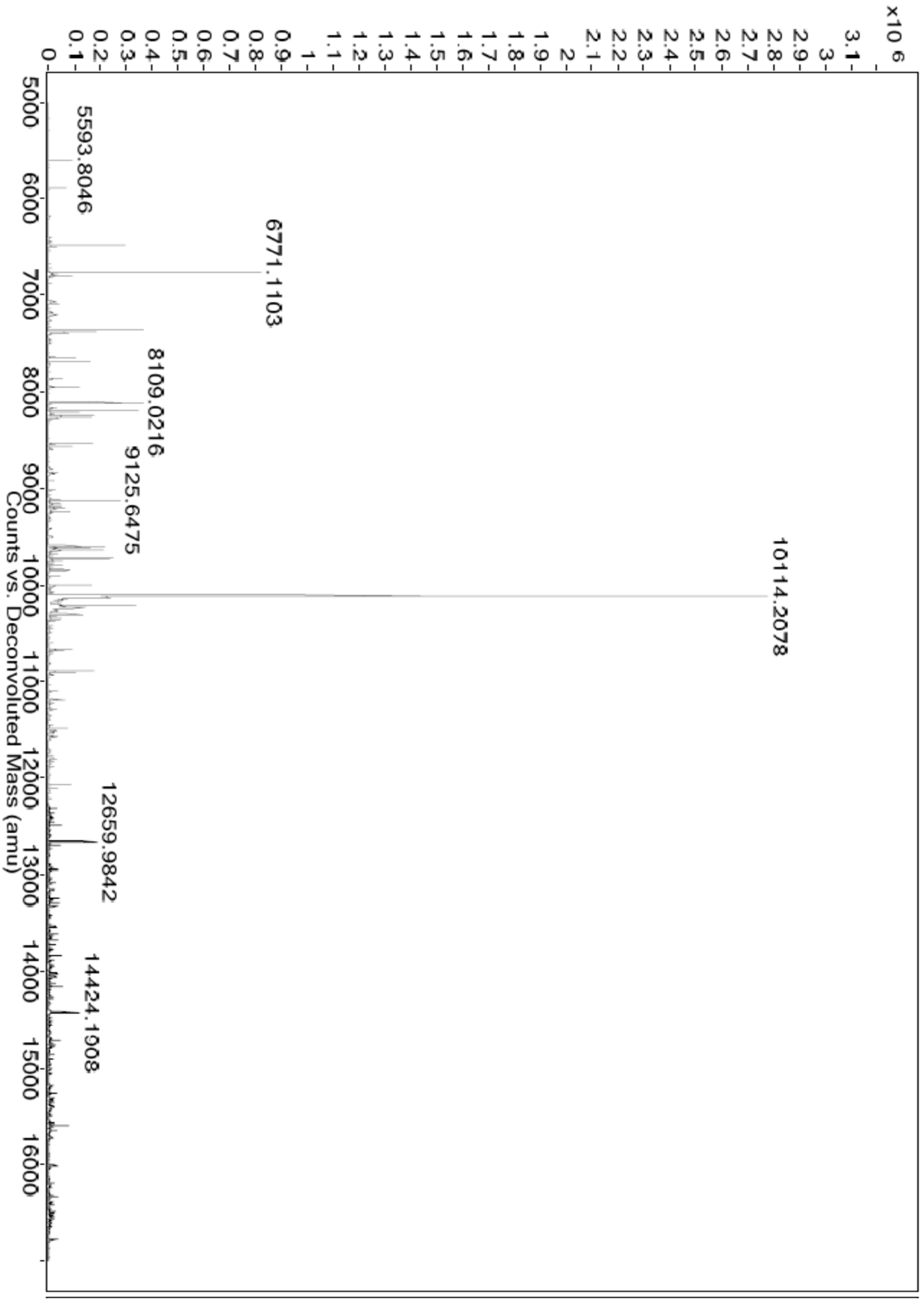

Figure S17. ESI-TOF MS of **G3.**

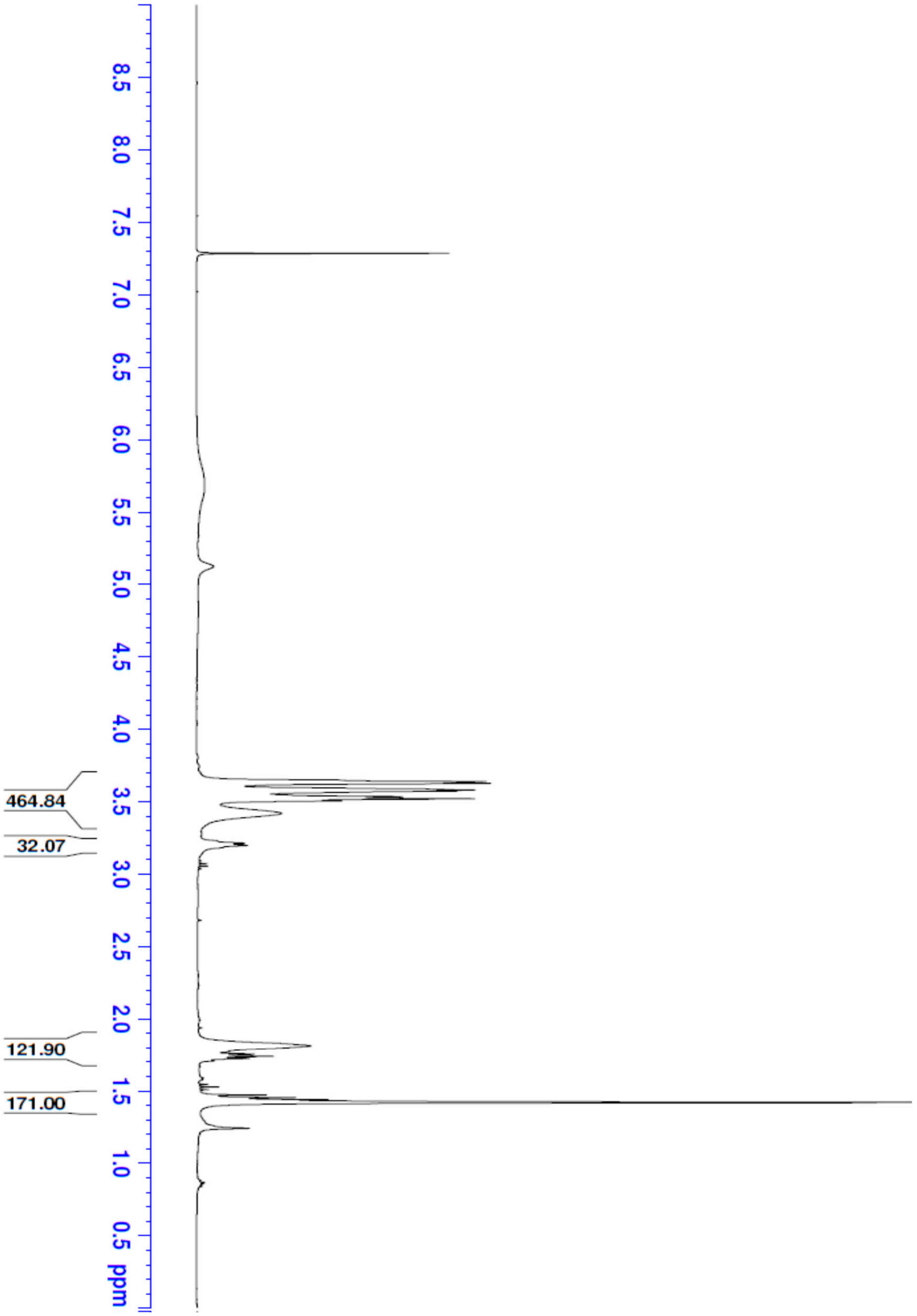

Figure S18. ^{1}H-NMR of **G3** (400 MHz, $CDCl_3$).

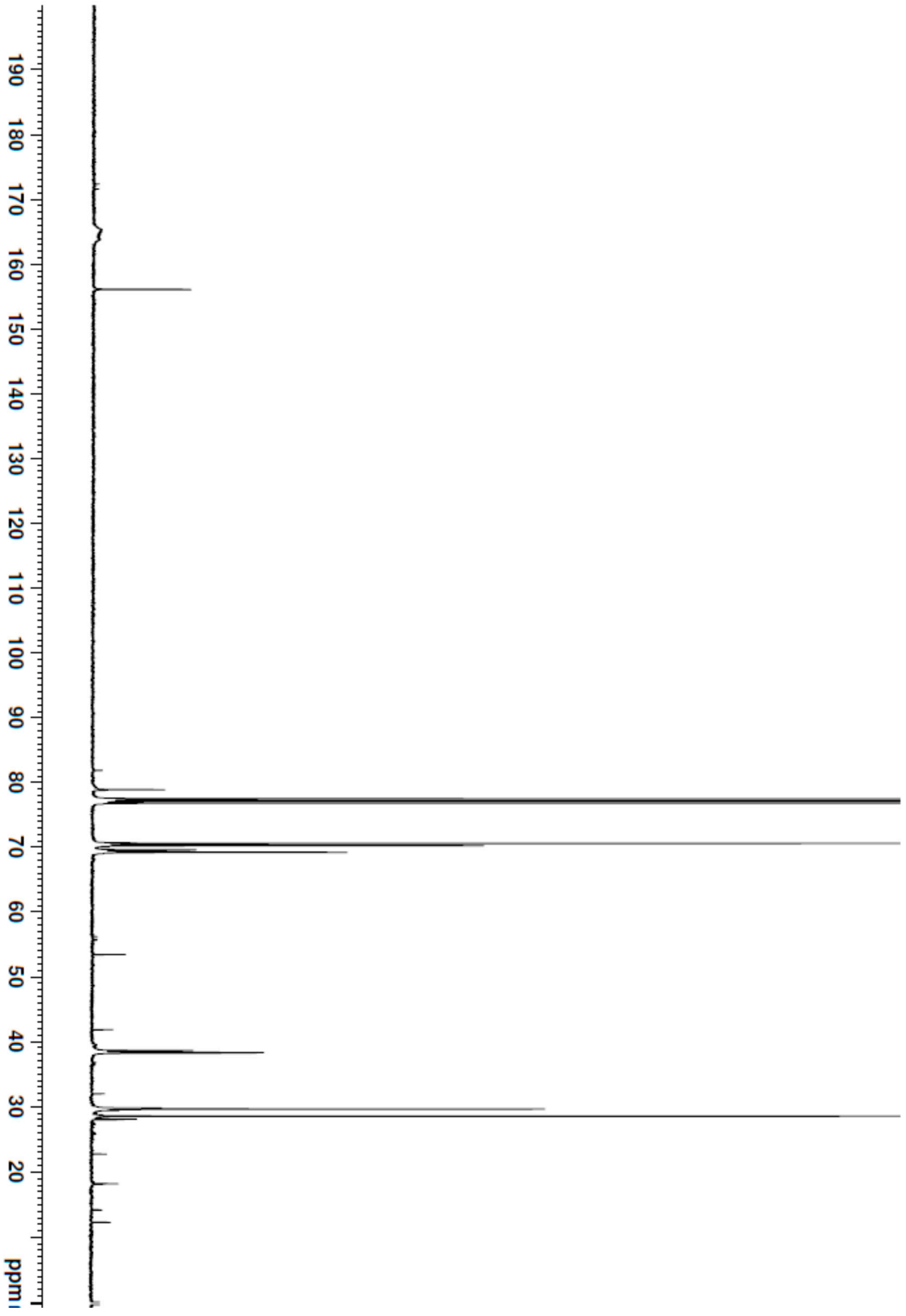

Figure S19. ^{13}C NMR of **G3** (100 MHz, $CDCl_3$).

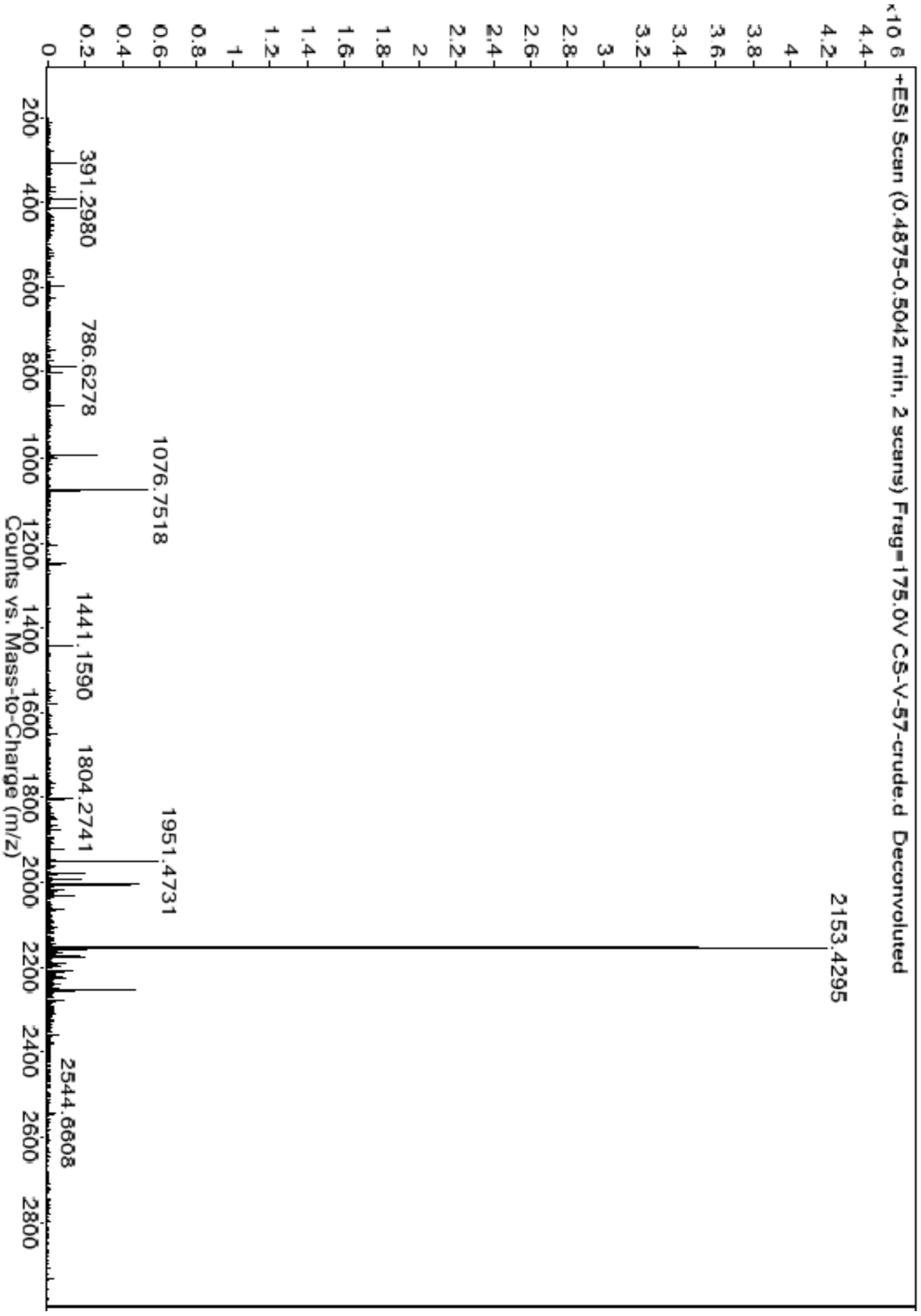

Figure S20. ESI-TOF MS of **6**. The line at *m*/*z* 1076 is the doubly charged species. The line at *m*/*z* 1951 is not identified.

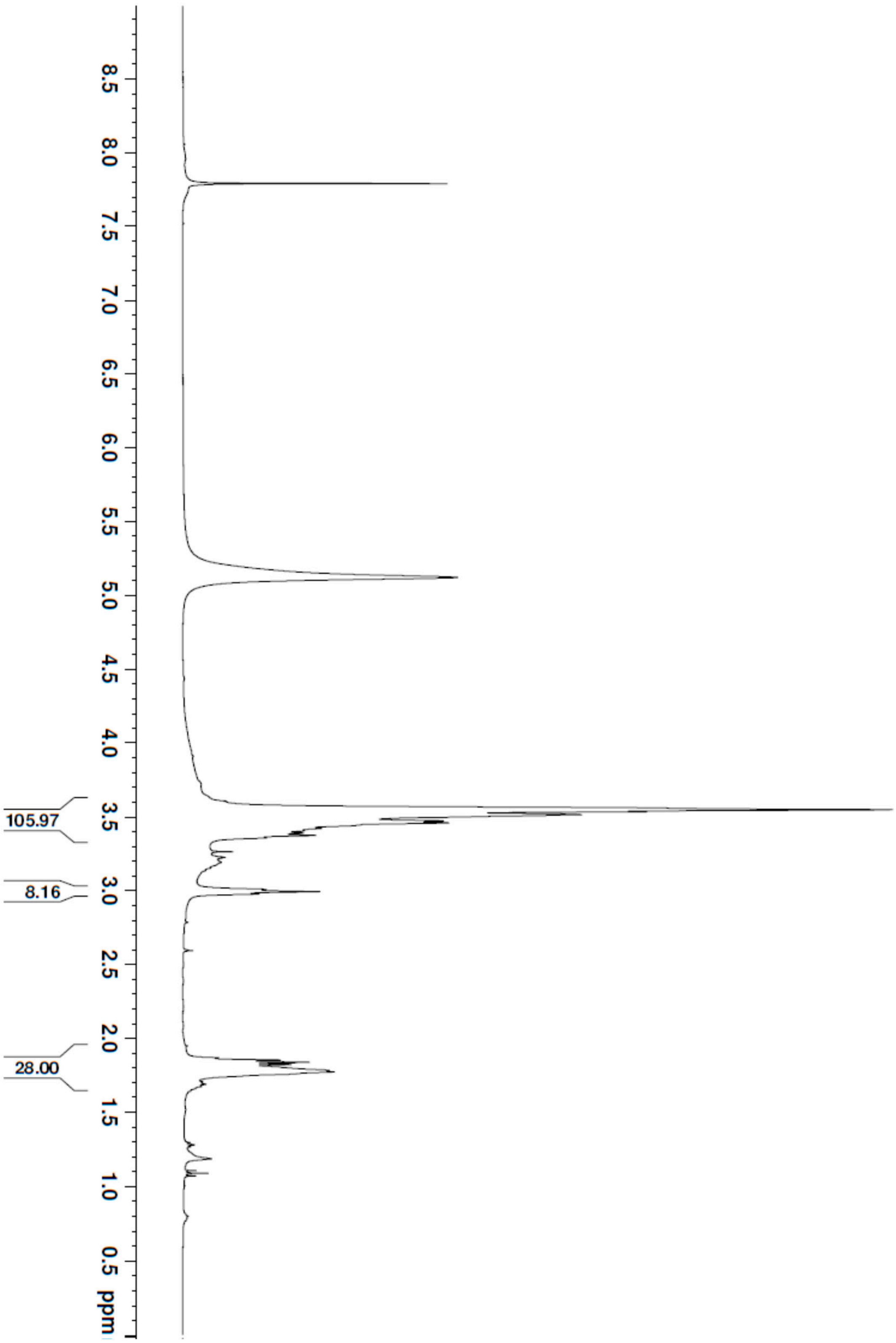

Figure S21. ^{1}H-NMR of **6** (400 MHz, CD_3OD).

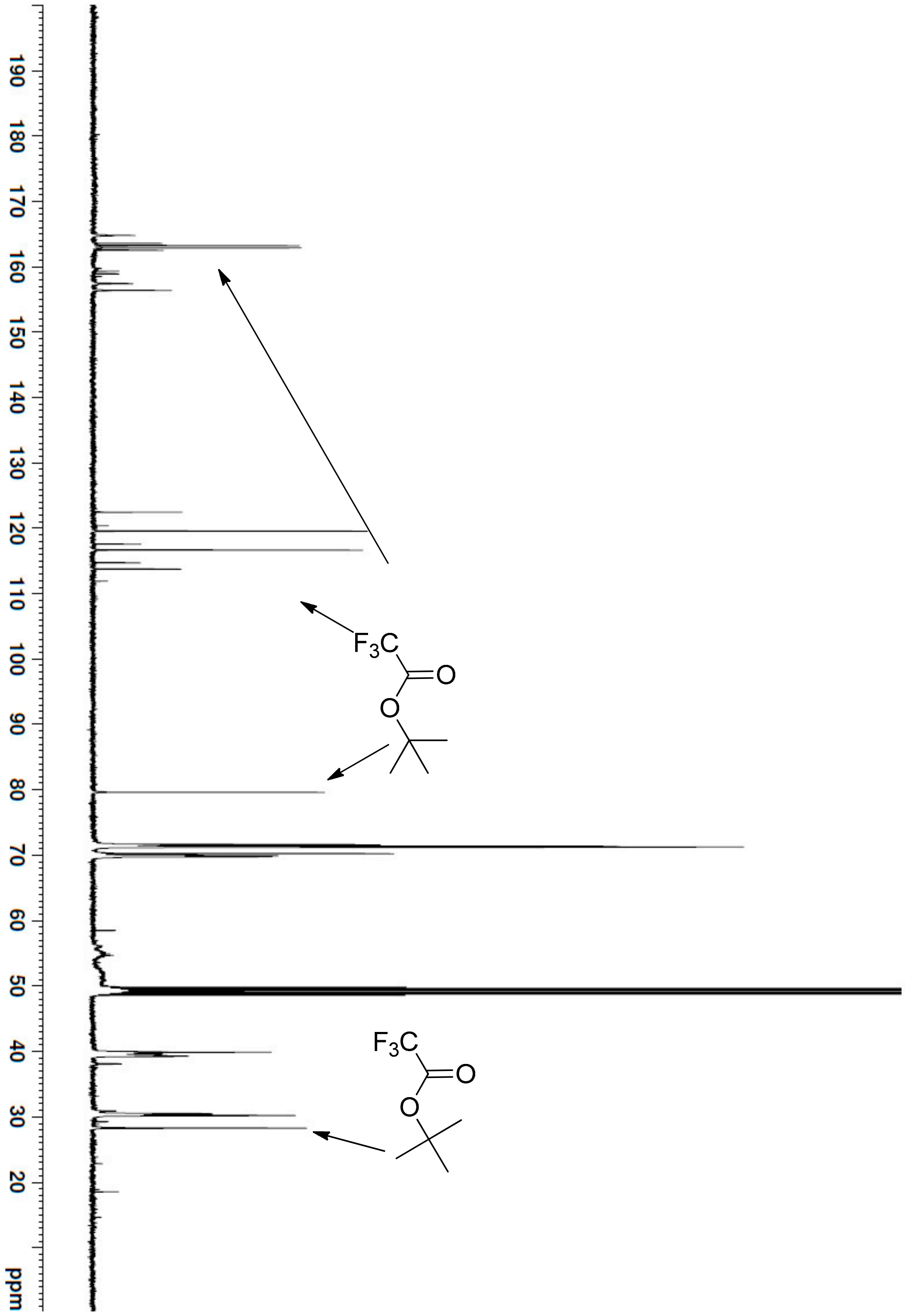

Figure S22. ^{13}C-NMR of **6** (100 MHz, CD_3OD). Lines assigned to t-butyl trifluoroacetate are identified.

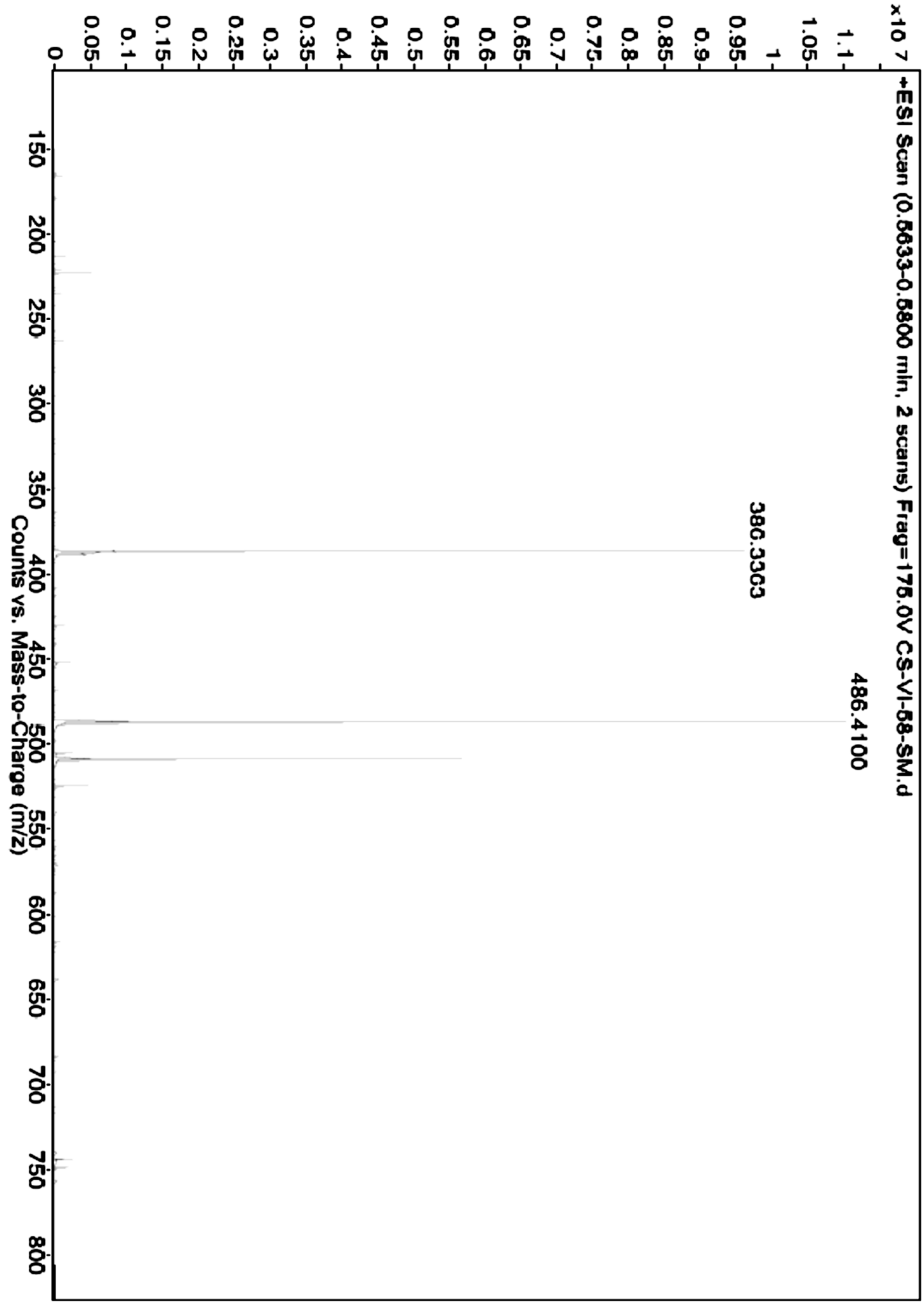

Figure S23. ESI-TOF MS of **7**. The line at *m*/*z* 386 is loss of BOC. Other lines correspond to sodium and potassium adducts.

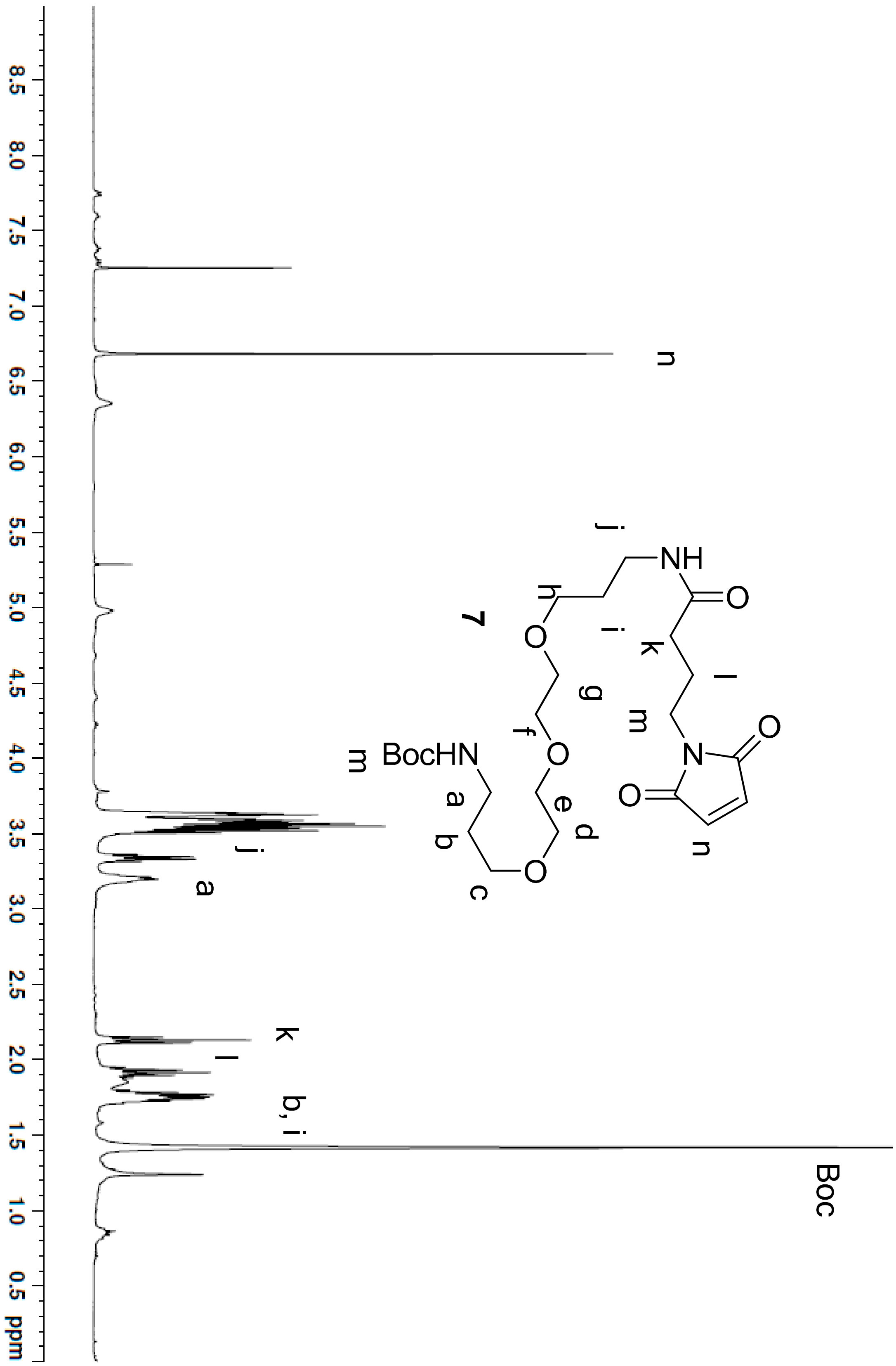

Figure S24. ^{1}H-NMR of **7** (400 MHz, $CDCl_3$).

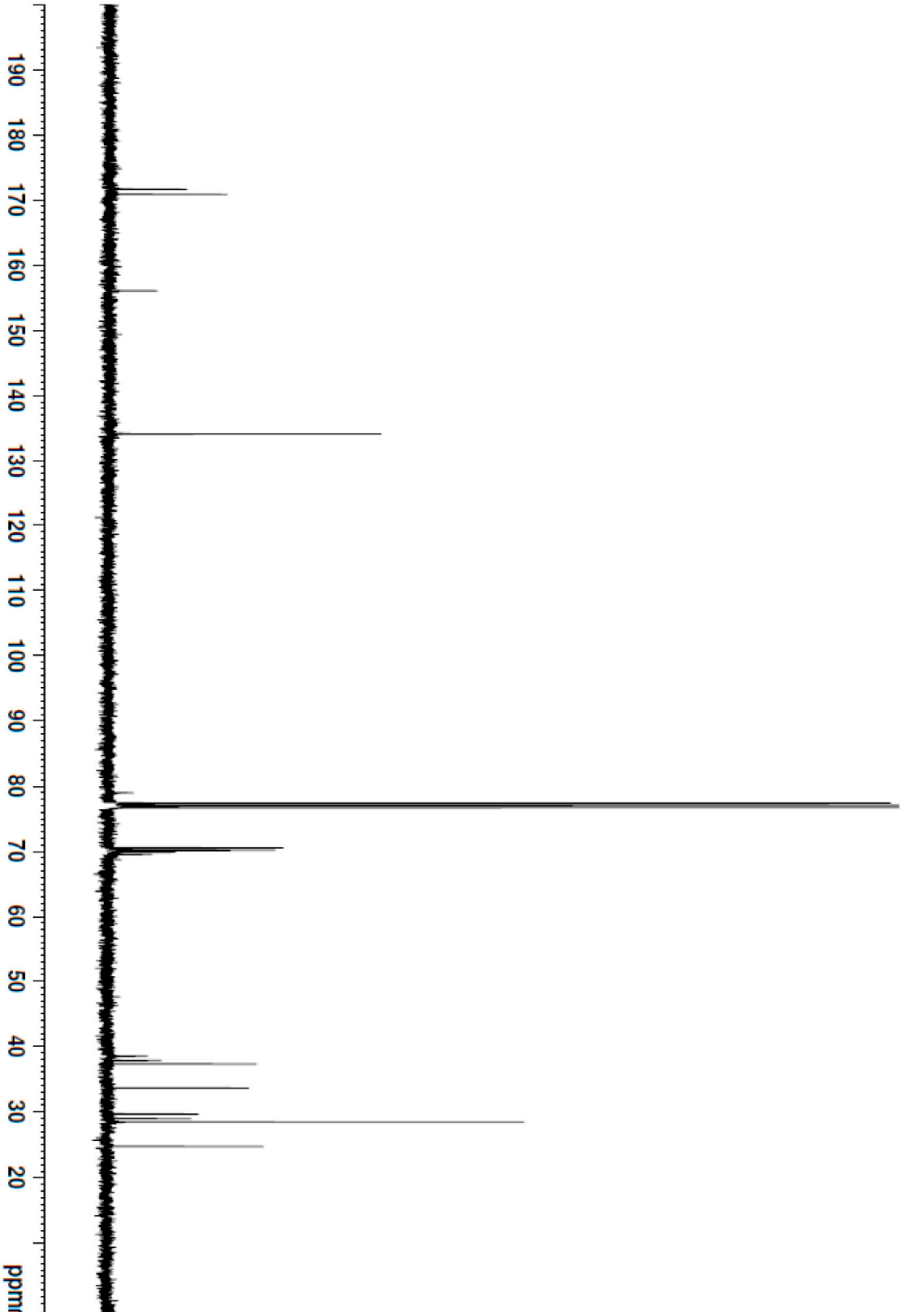

Figure S25 ^{13}C-NMR of **7** (100 MHz, $CDCl_3$).

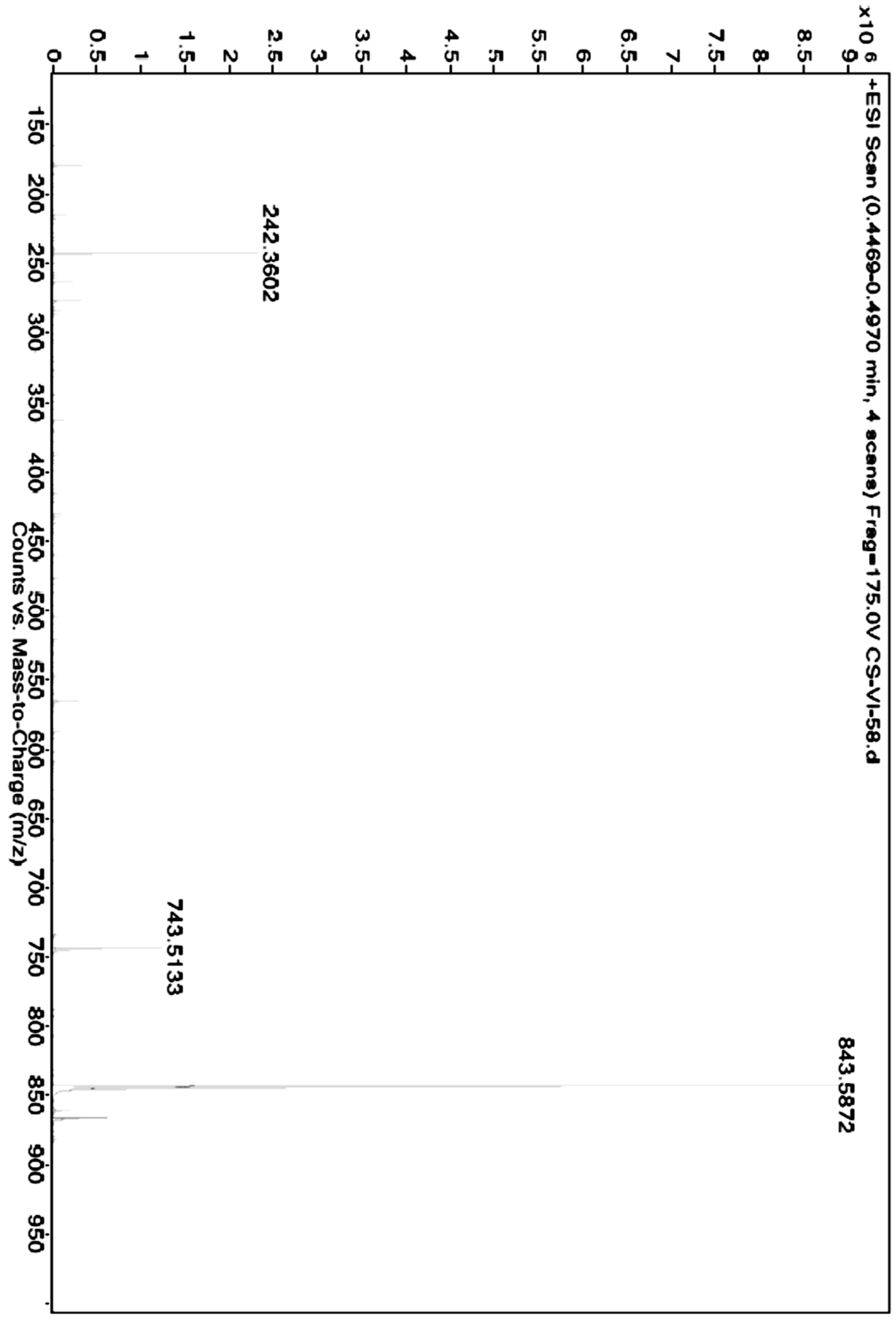

Figure S26. ESI-TOF MS of **7-A**. The spectrum also shows loss of BOC. The line at *m*/*z* 242 is not identified.

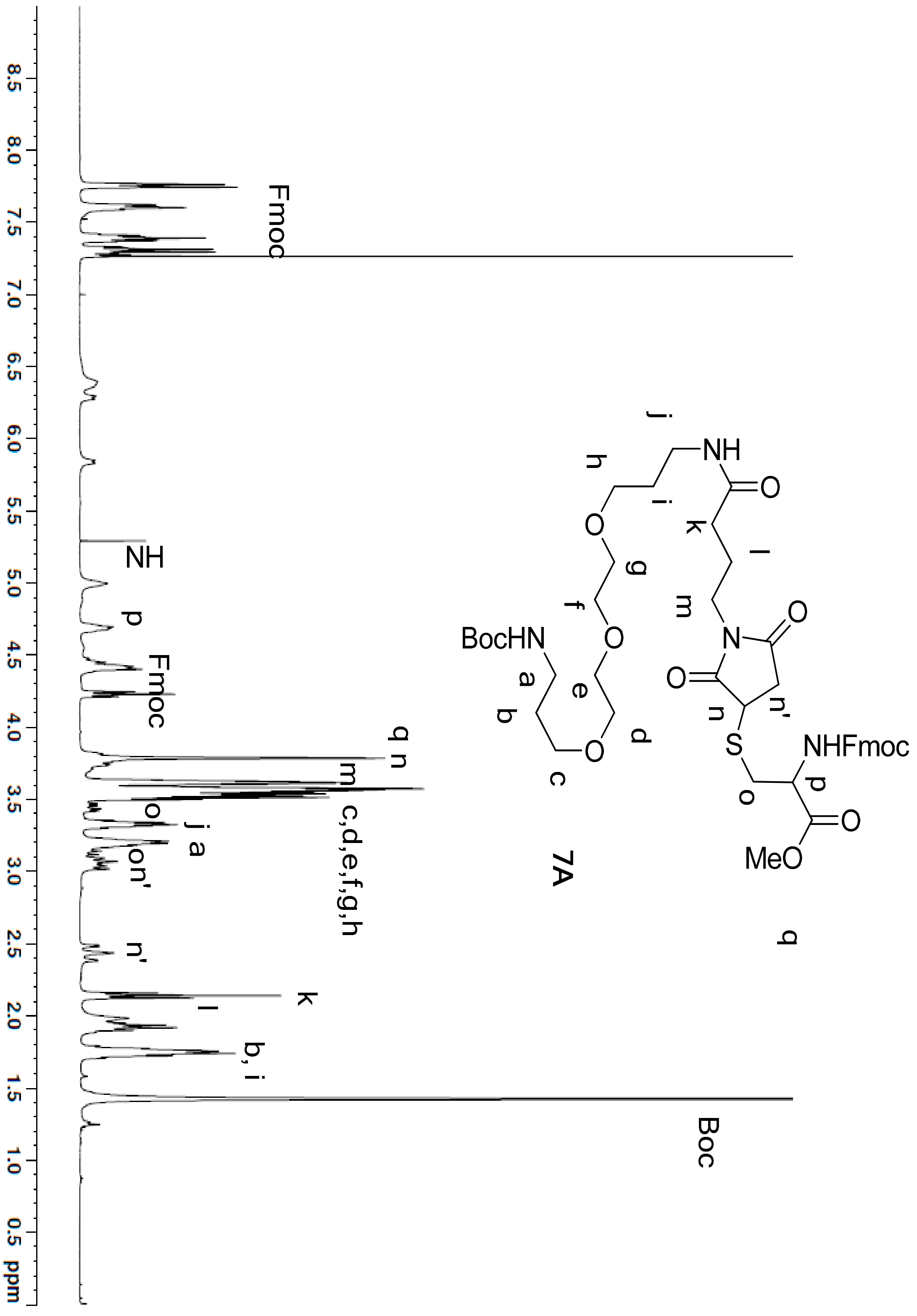

Figure S27. ^{1}H-NMR of **7-A** (400 MHz, $CDCl_3$).

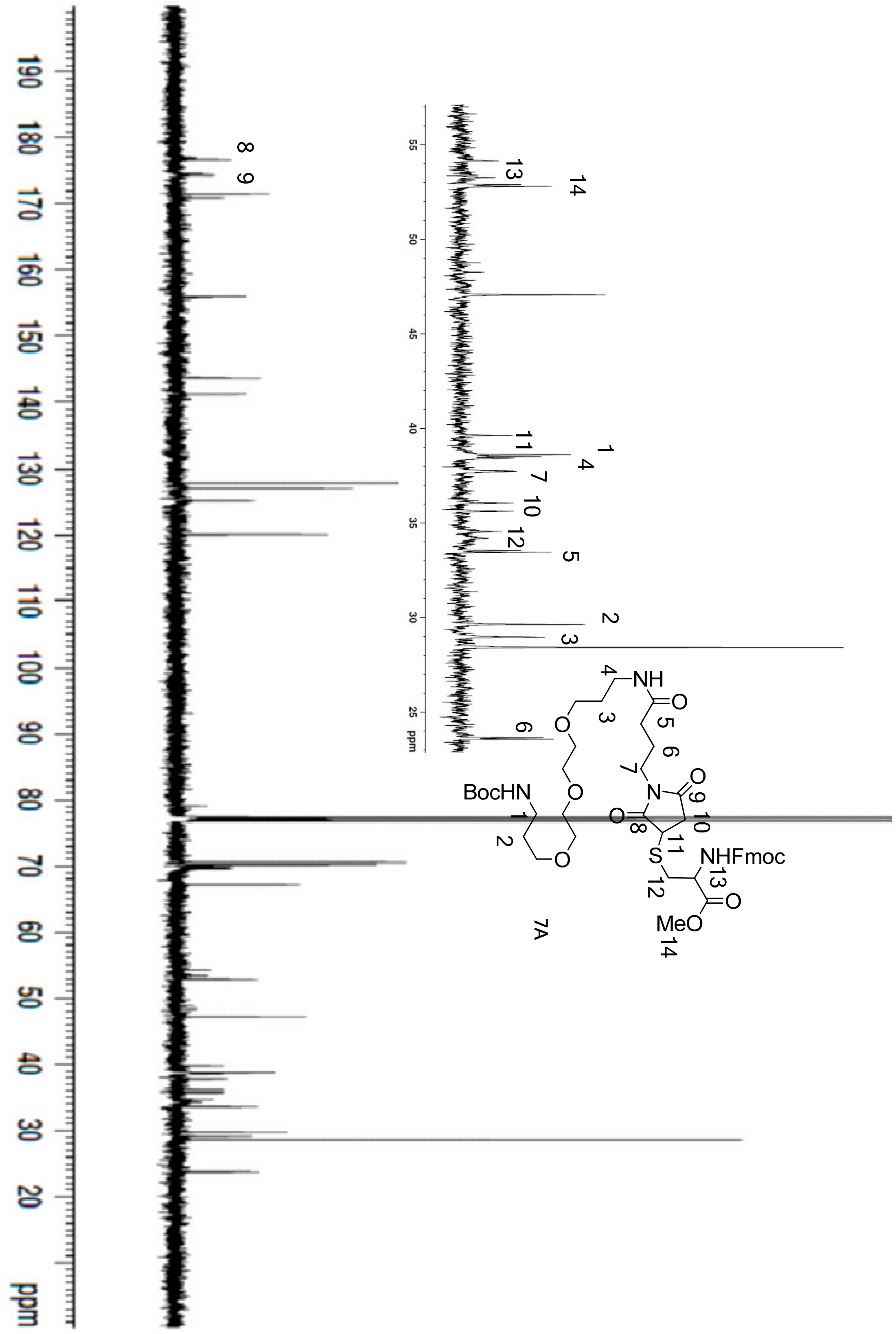

Figure S28. ^{13}C NMR of **7-A** (100 MHz, $CDCl_3$) with inset.

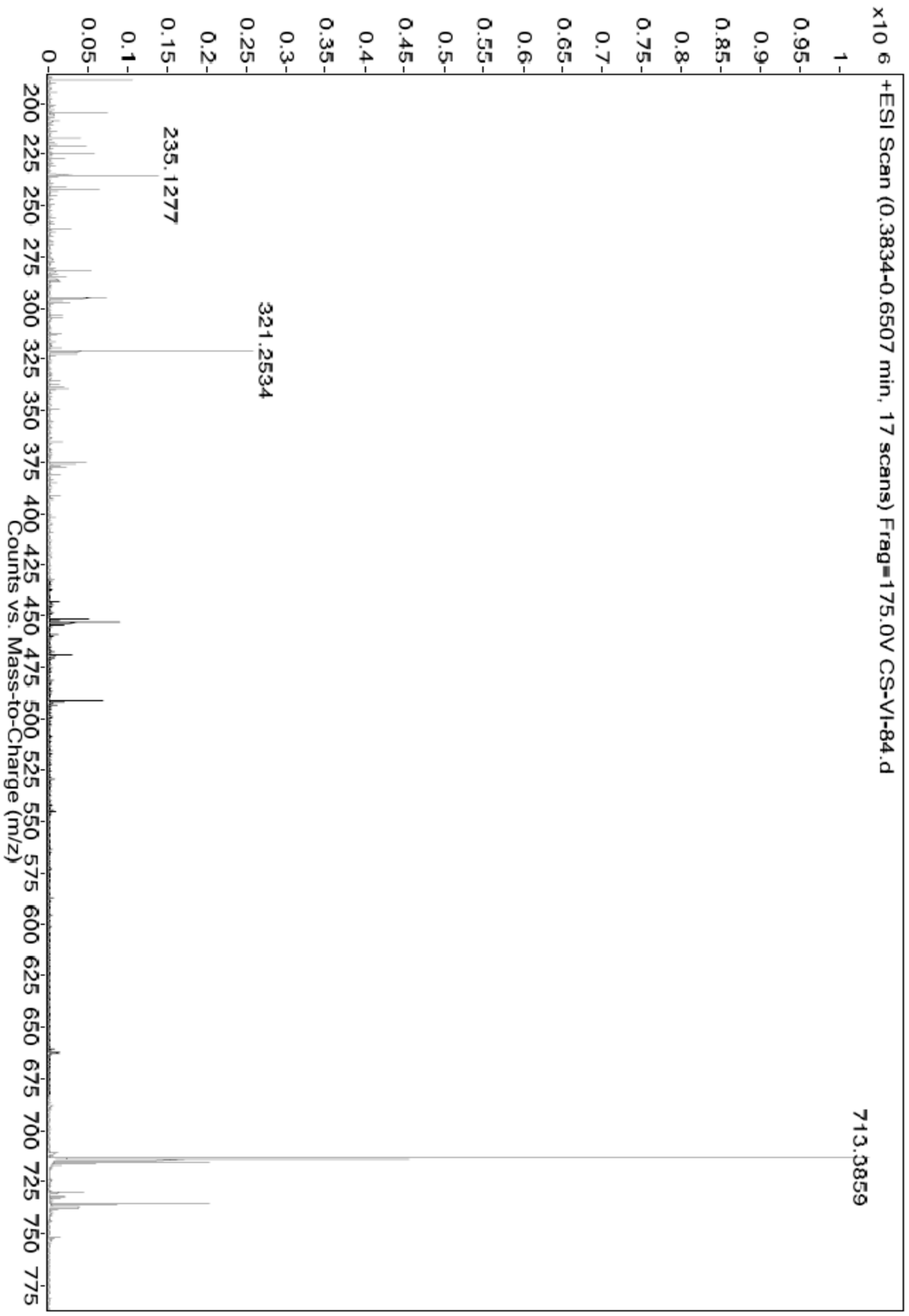

Figure S29. ESI-TOF MS of cysteine.

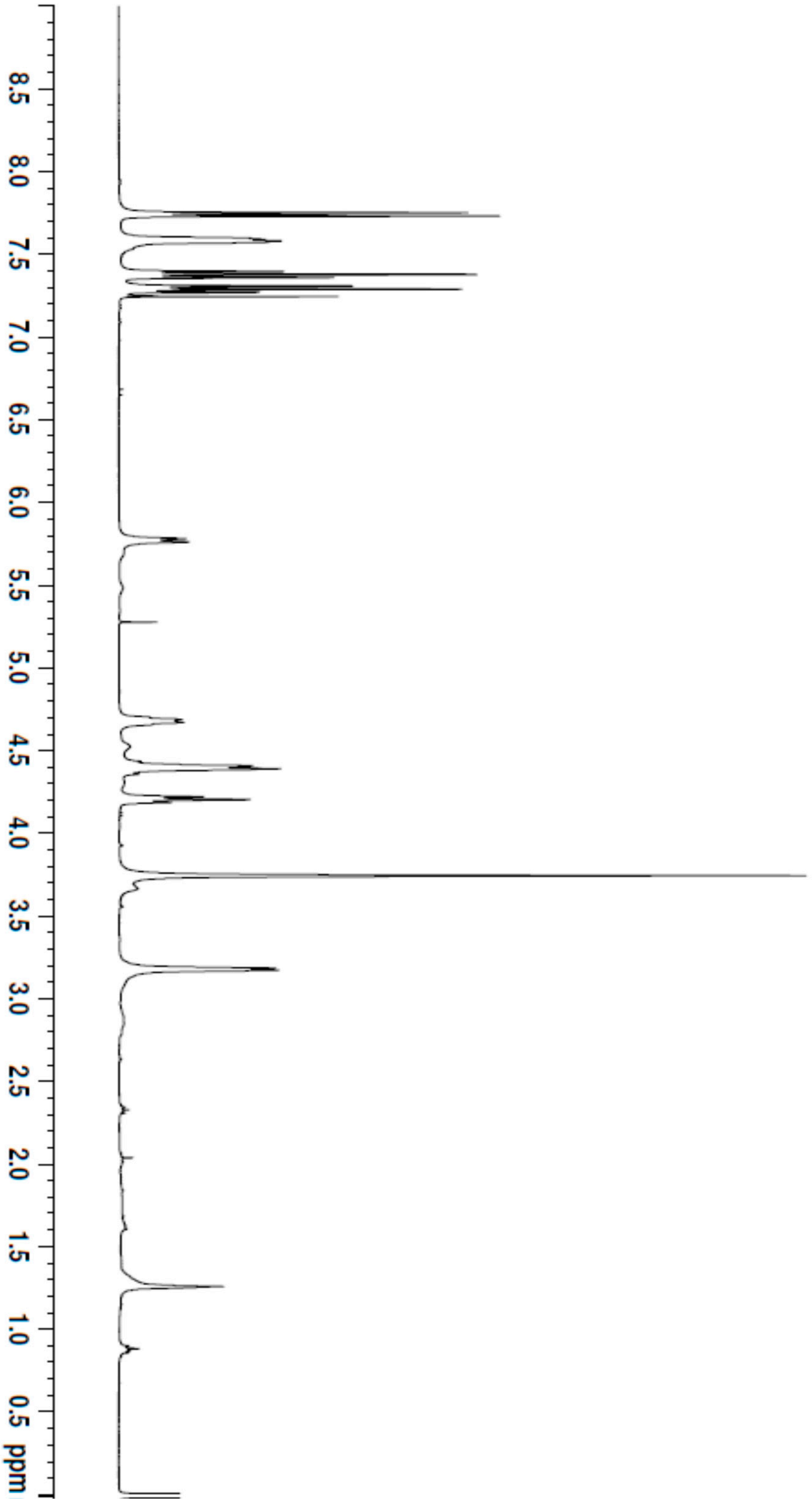

Figure S30. ^{1}H-NMR of cystine (400 MHz, $CDCl_3$).

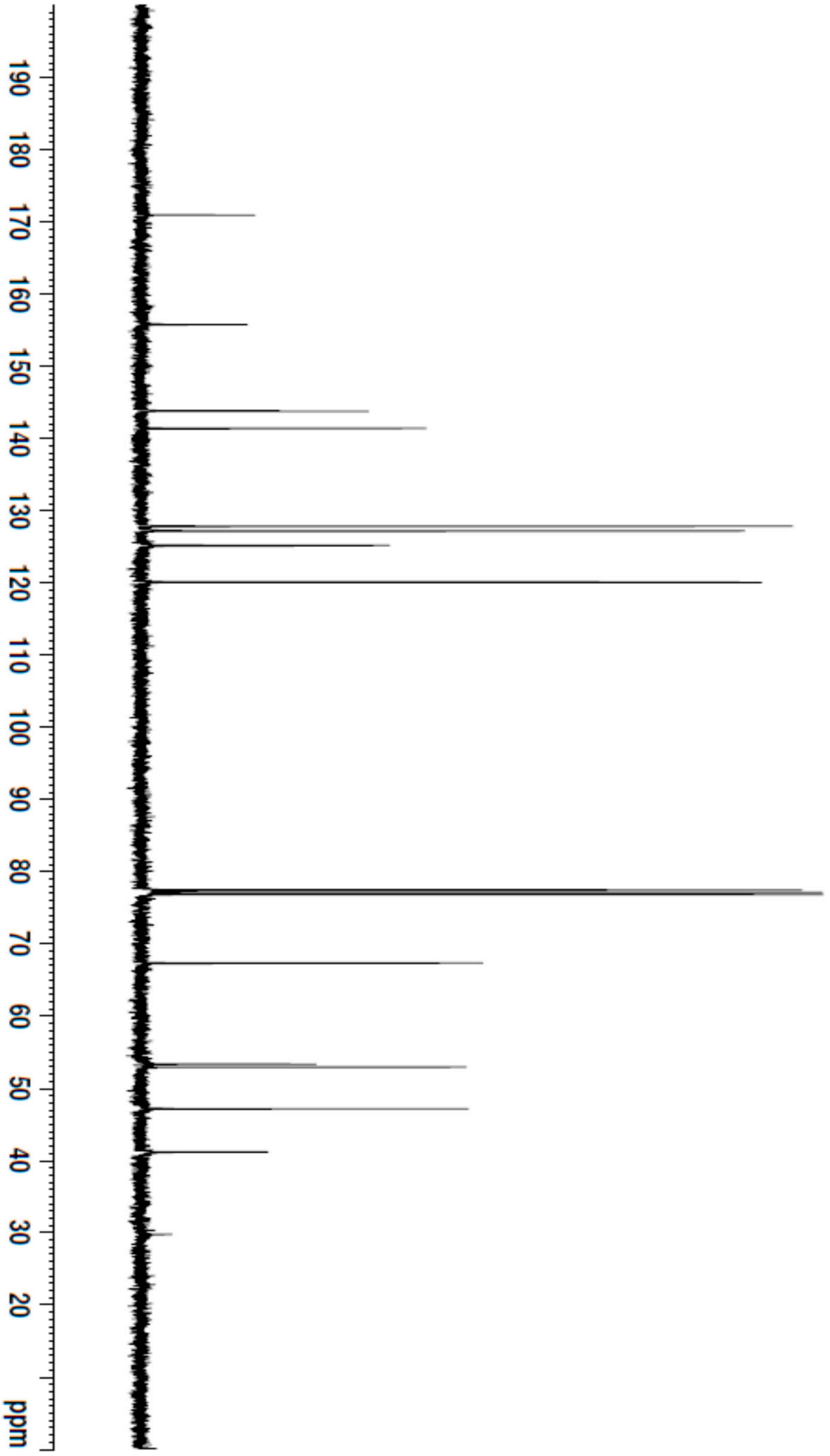

Figure S31. ^{13}C NMR of cystine (100 MHz, $CDCl_3$).

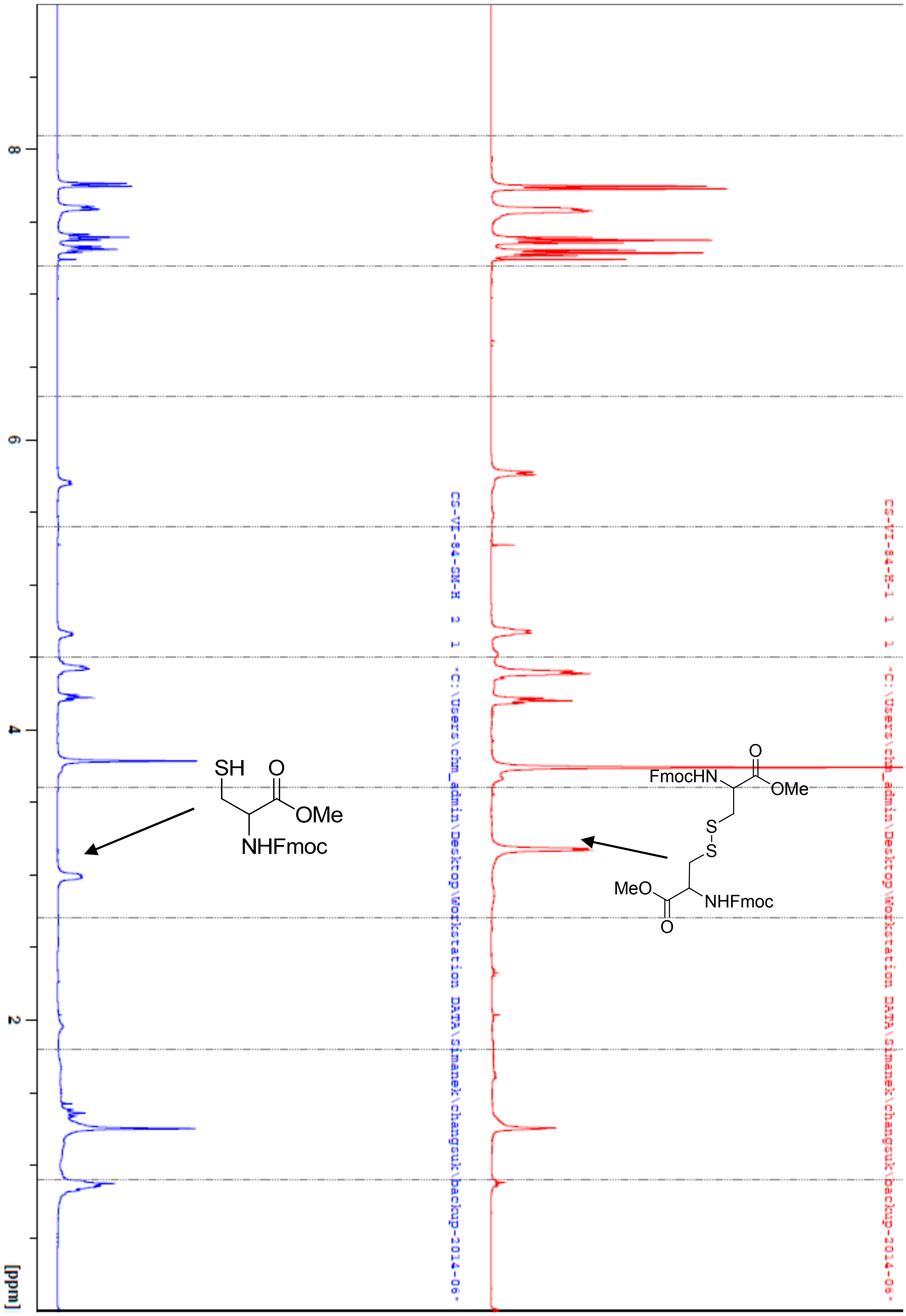

Figure S32. Comparison of **A** and cystine by ^{1}H-NMR (400 MHz, $CDCl_3$).

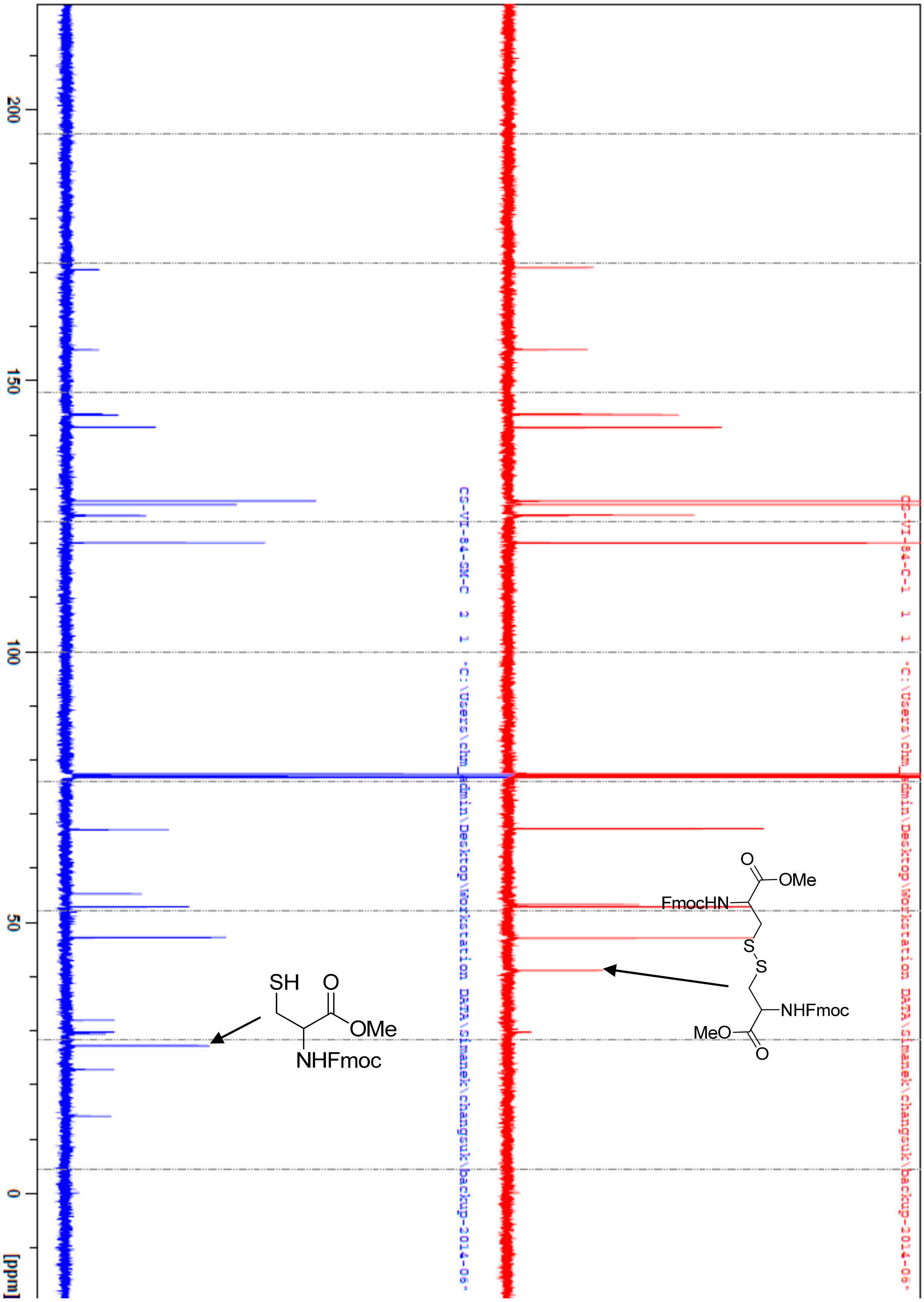

Figure S33. Comparison of **A** and cystine by ^{13}C-NMR

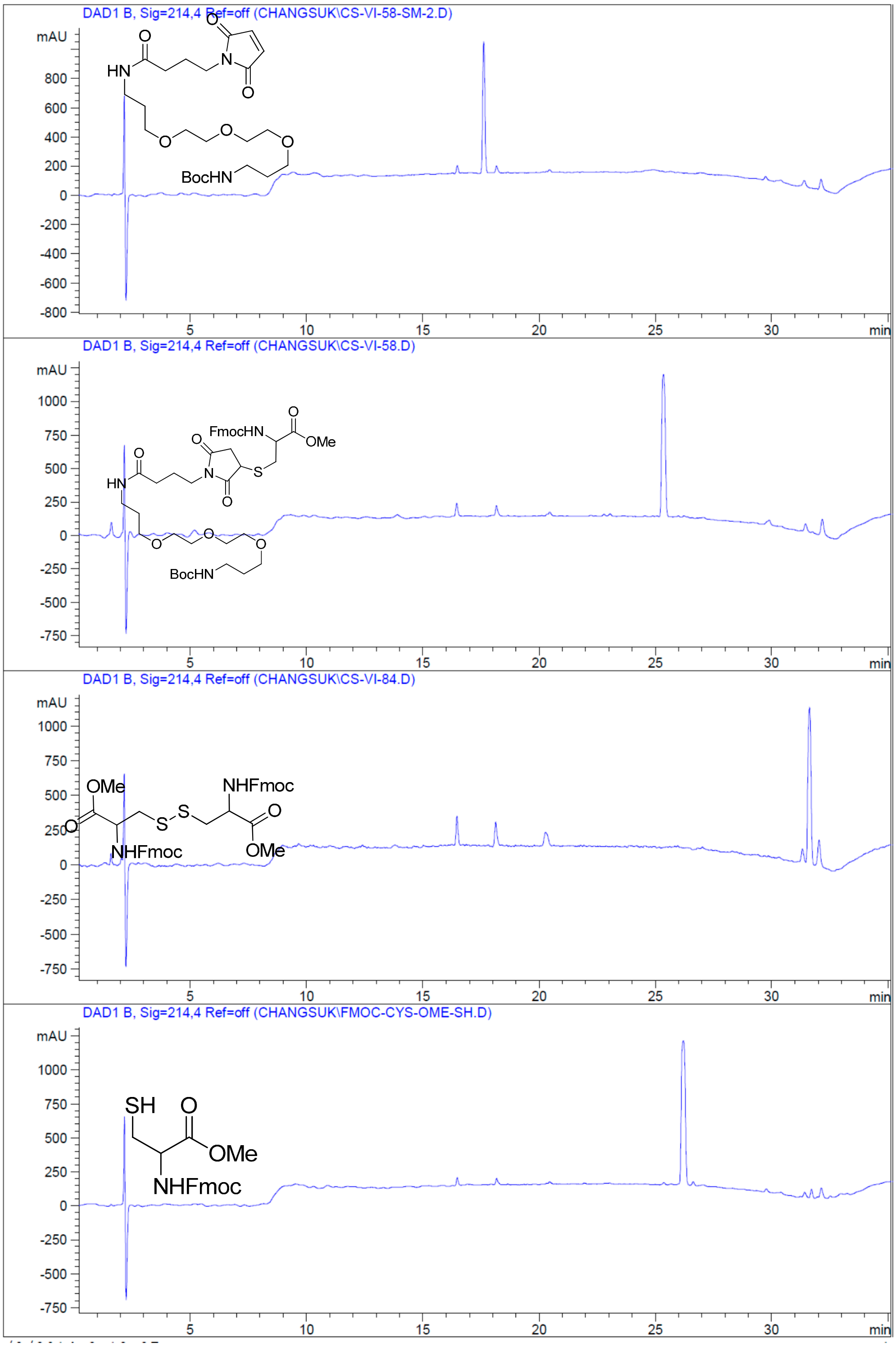

Figure S34. Comparison by HPLC of **7**, **7-A**, and cystine. The mobile phase consisted of water/acetonitrile (A/B, HPLC grade, 0.1% (*w*/*v*) trifluoroacetic acid) at a flow rate of 0.8 mL/min. The elution gradient was 10% MeCN for 5 min, ramp to 90% MeCN in 30 min, and ramp down to 10% MeCN in 15 min. The sample volume injected 5 μL at a concentration of 0.1 mg/mL (Acetonitrile) of each samples and eluted sample was detected at 214 nm.

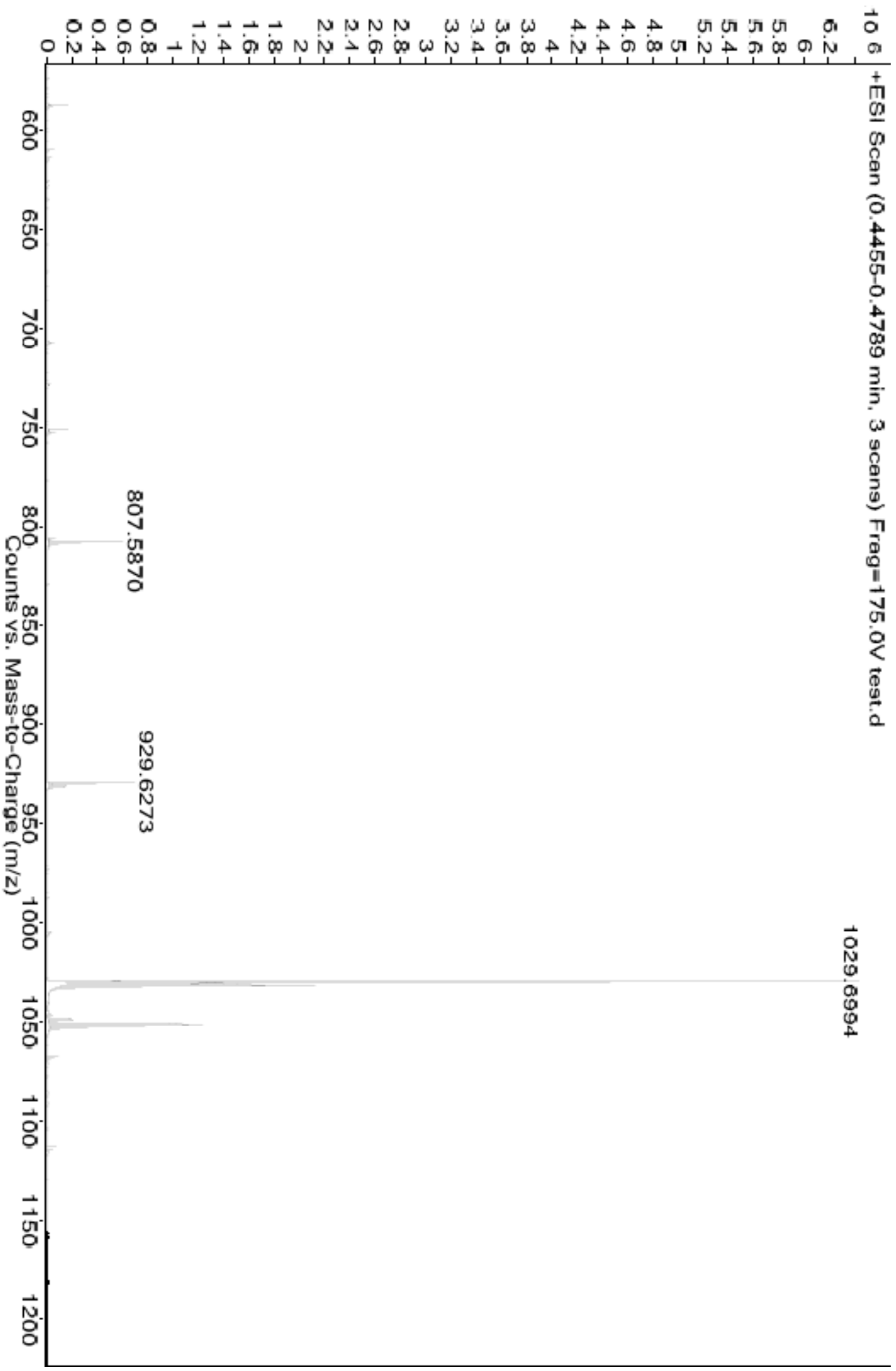

Figure S35. ESI-TOF MS of **7**-**B**. The trace shows loss of BOC. The line at *m*/*z* 807 is not identified.

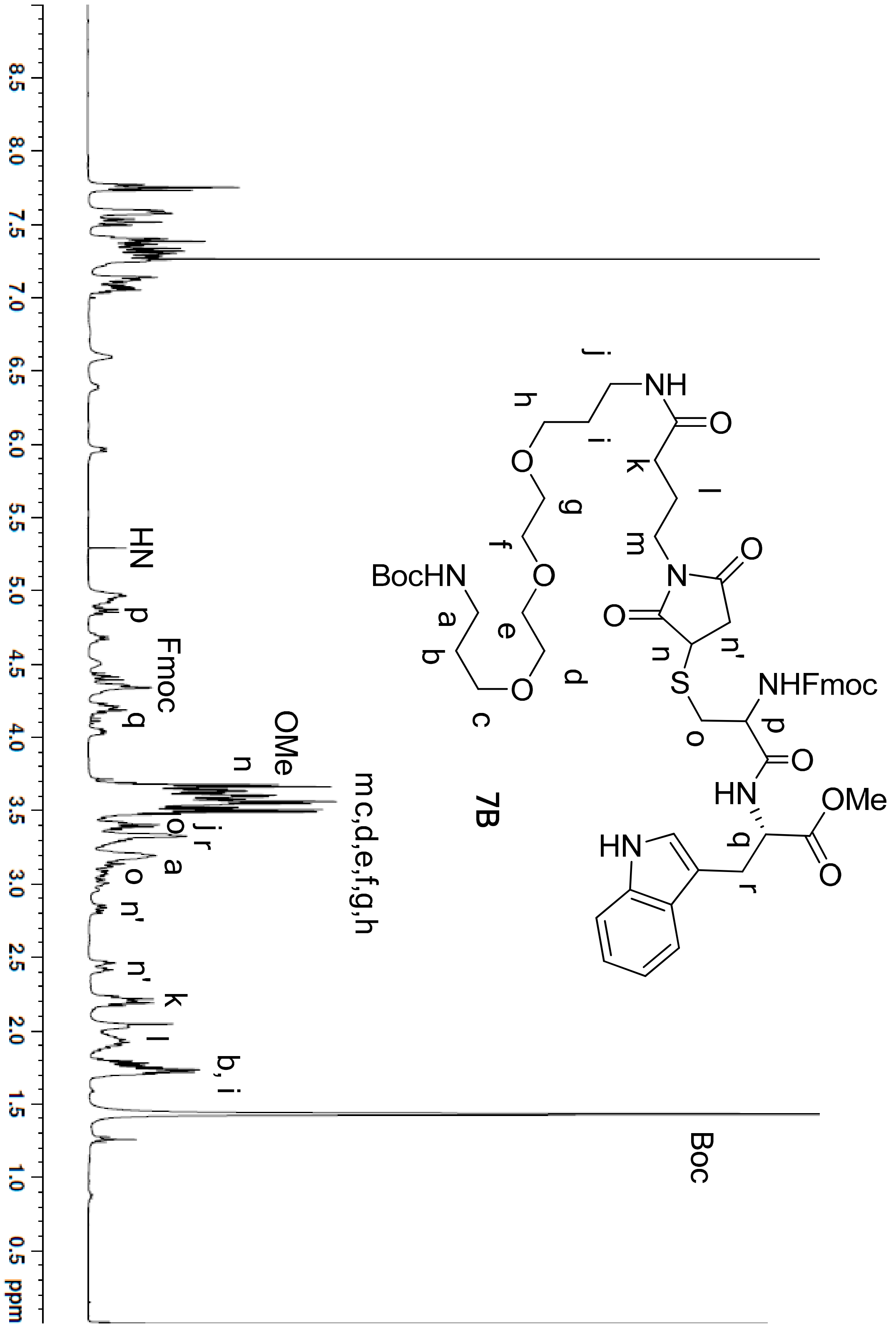

Figure S36. ^{1}H-NMR of **7-B** (400 MHz, $CDCl_3$).

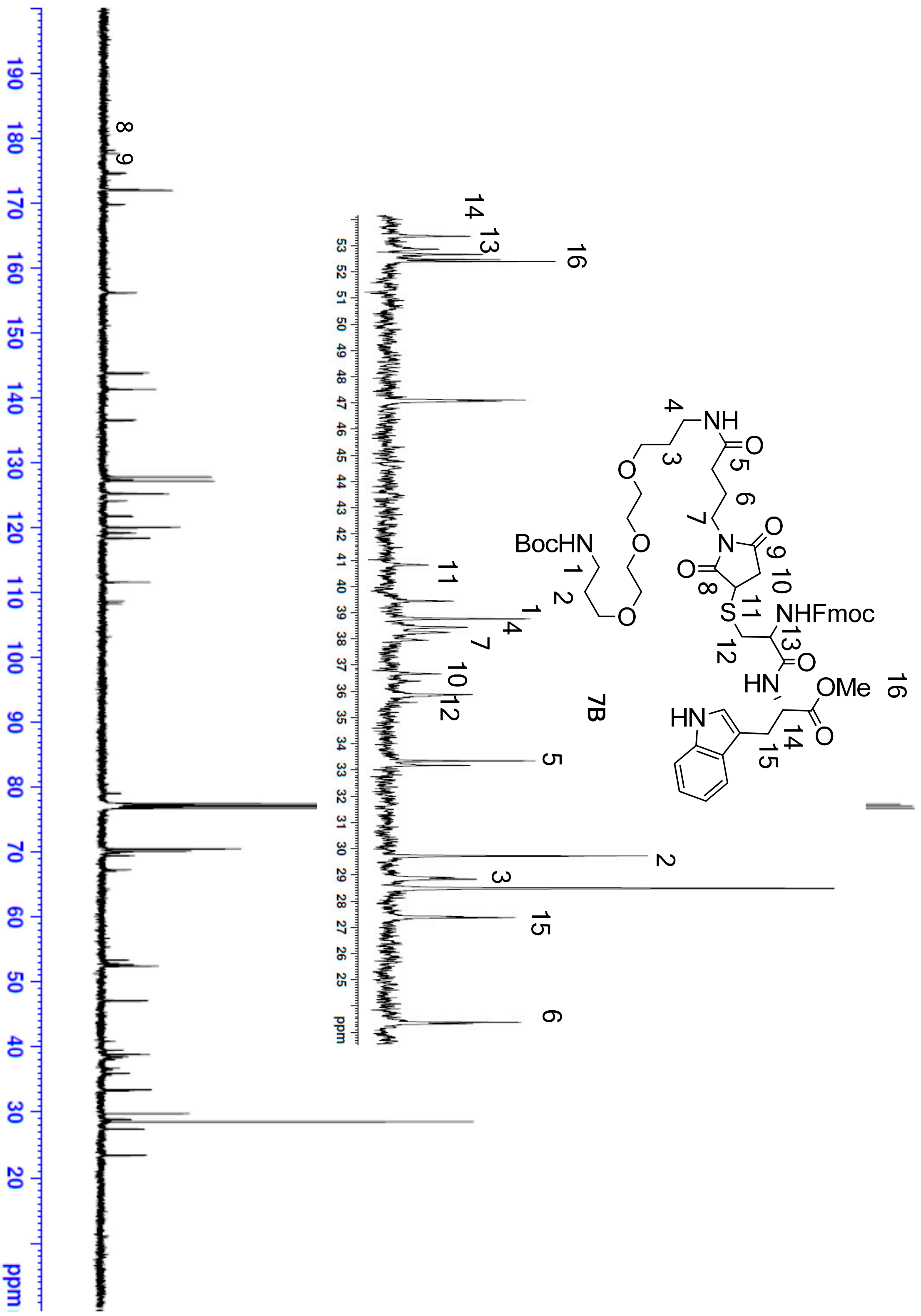

Figure S37. ^{13}C-NMR of **7**-**B** (100 MHz, $CDCl_3$).

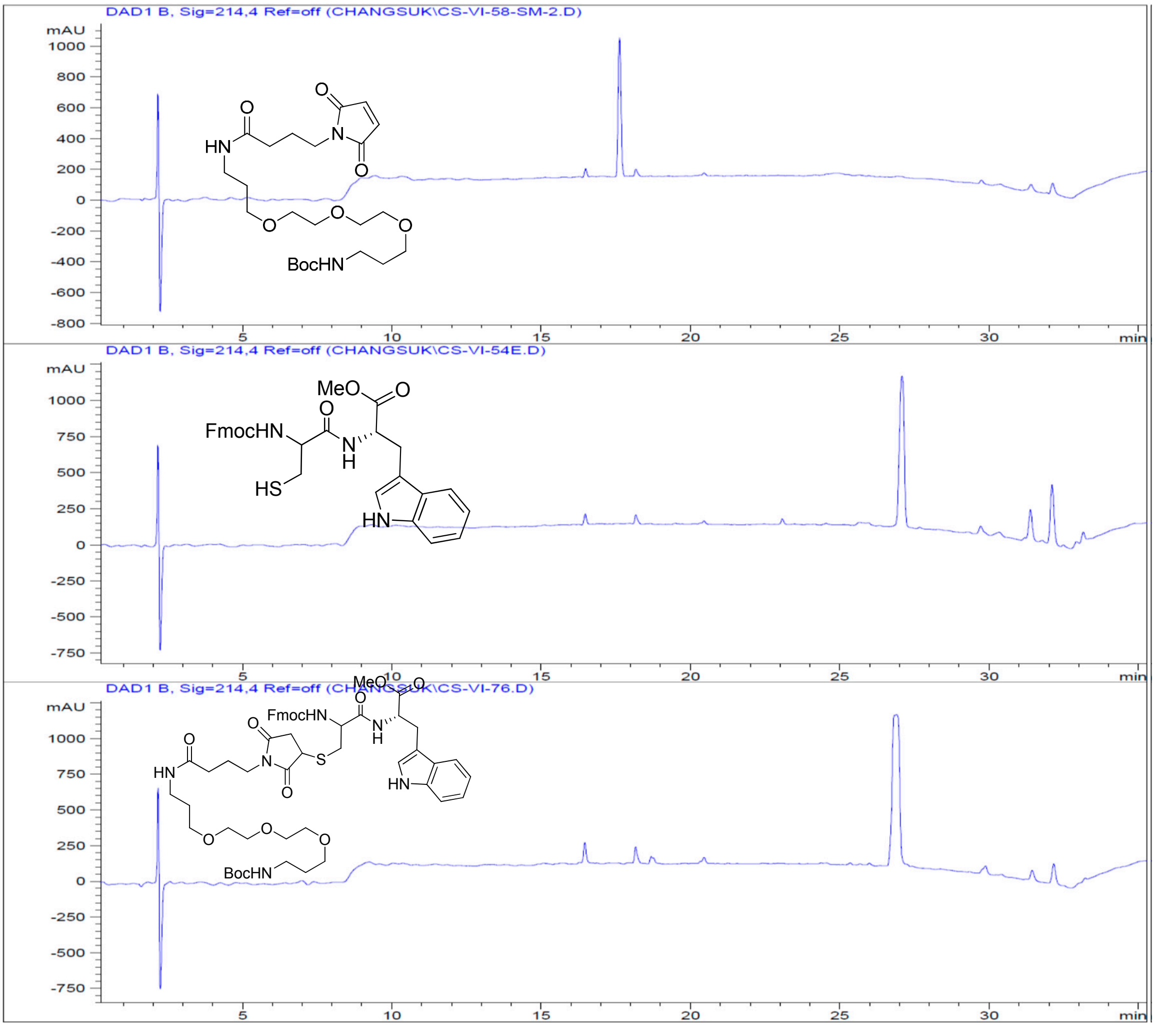

Figure S38. Comparison by HPLC of **7**, **7-B**, and cysteine. The mobile phase consisted of water/acetonitrile (A/B, HPLC grade, 0.1% (*w*/*v*) trifluoroacetic acid) at a flow rate of 0.8 mL/min. The elution gradient was 10% MeCN for 5 min, ramp to 90% MeCN in 30 min, and ramp down to 10% MeCN in 15 min. The sample volume injected 5 μL at a concentration of 0.1 mg/mL (Acetonitrile) of each samples and eluted sample was detected at 214 nm.

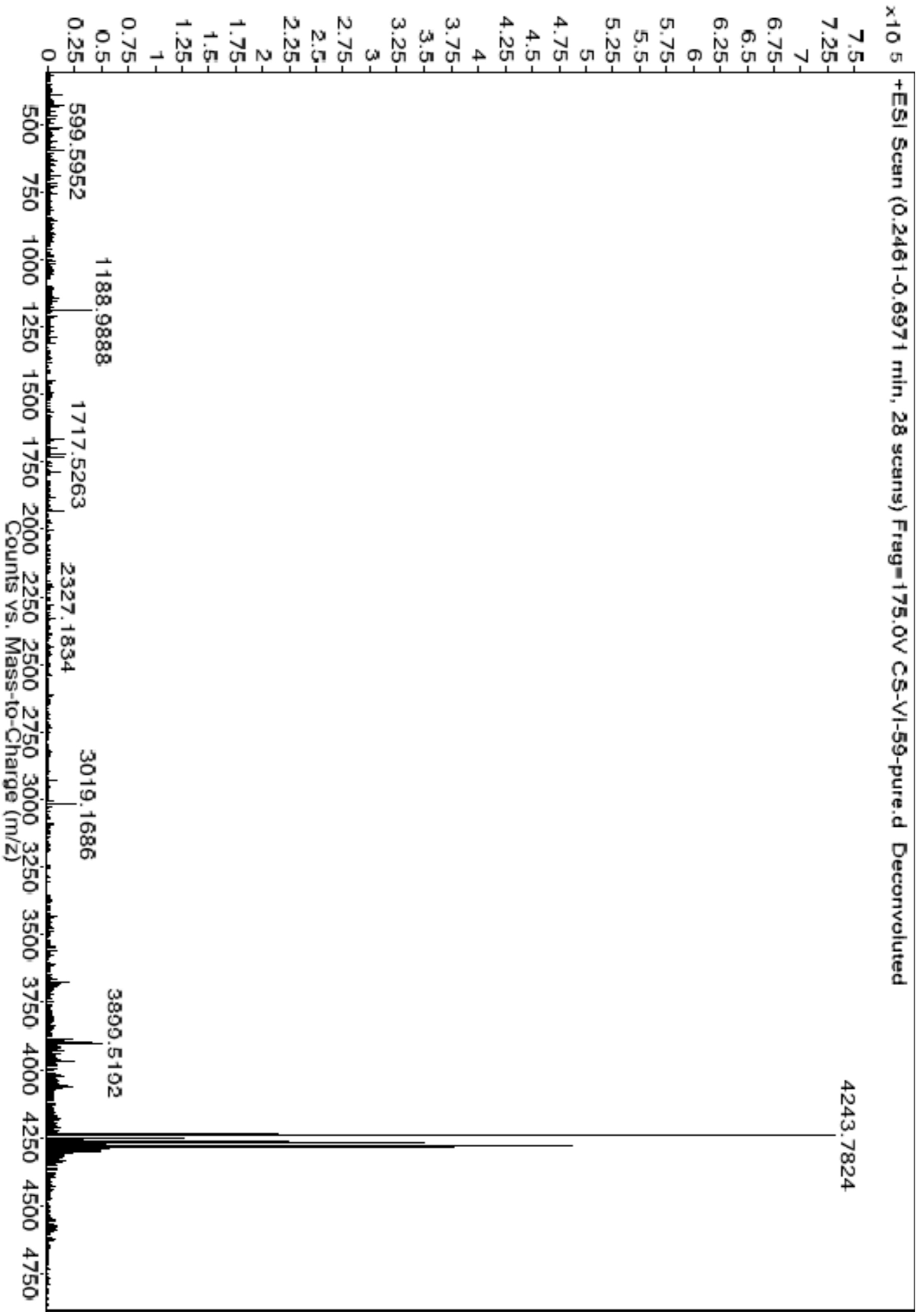

Figure S39. ESI-TOF MS of **1-A**$_4$. The line at m/z 3899 corresponds to **1-A**$_3$.

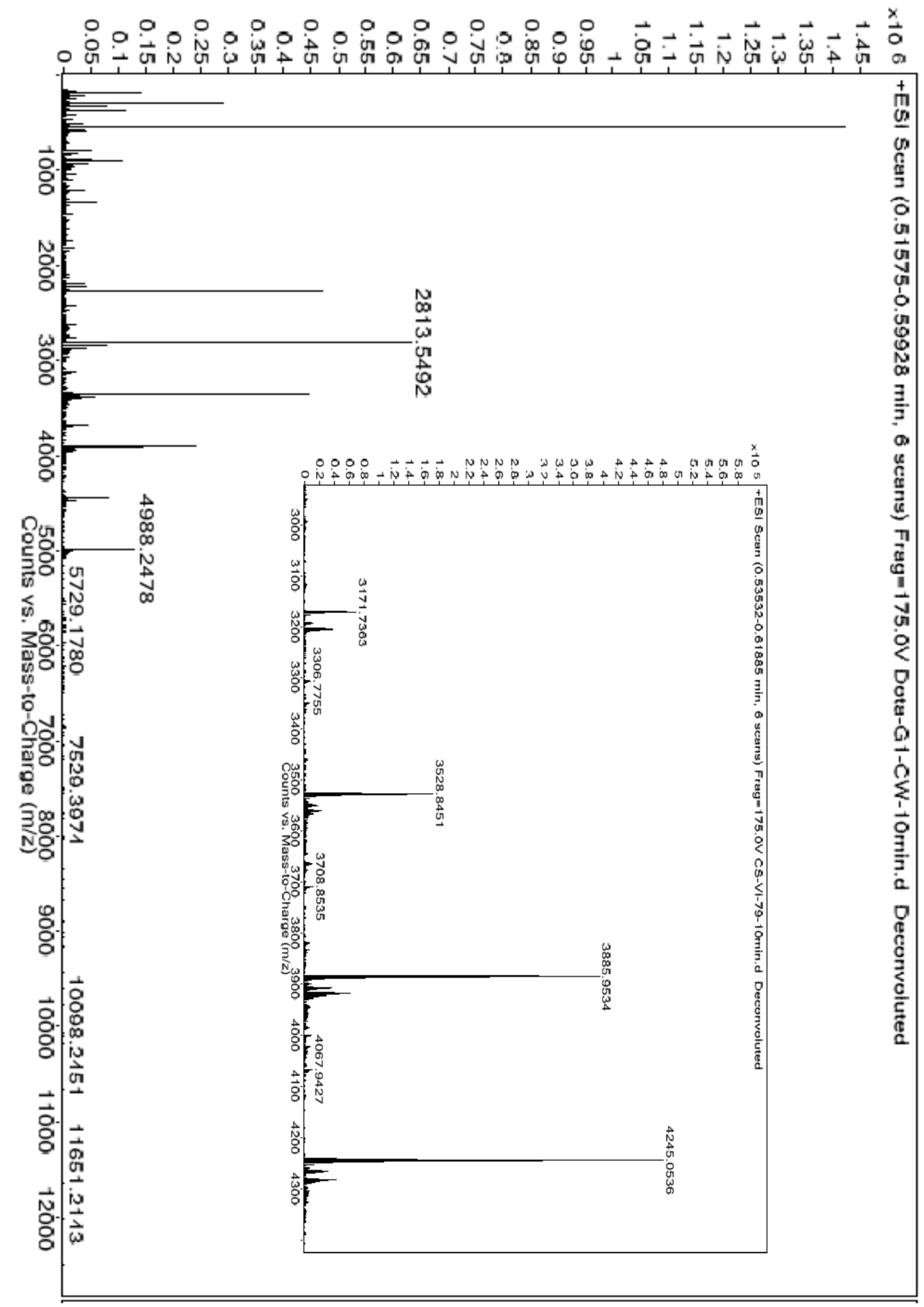

Figure S40. ESI-TOF MS of **1-A4** after 10 min. Lines corresponding to **1**, **1-A1, 1-A2, 1-A3,** and **1-A4** are clearly visible.

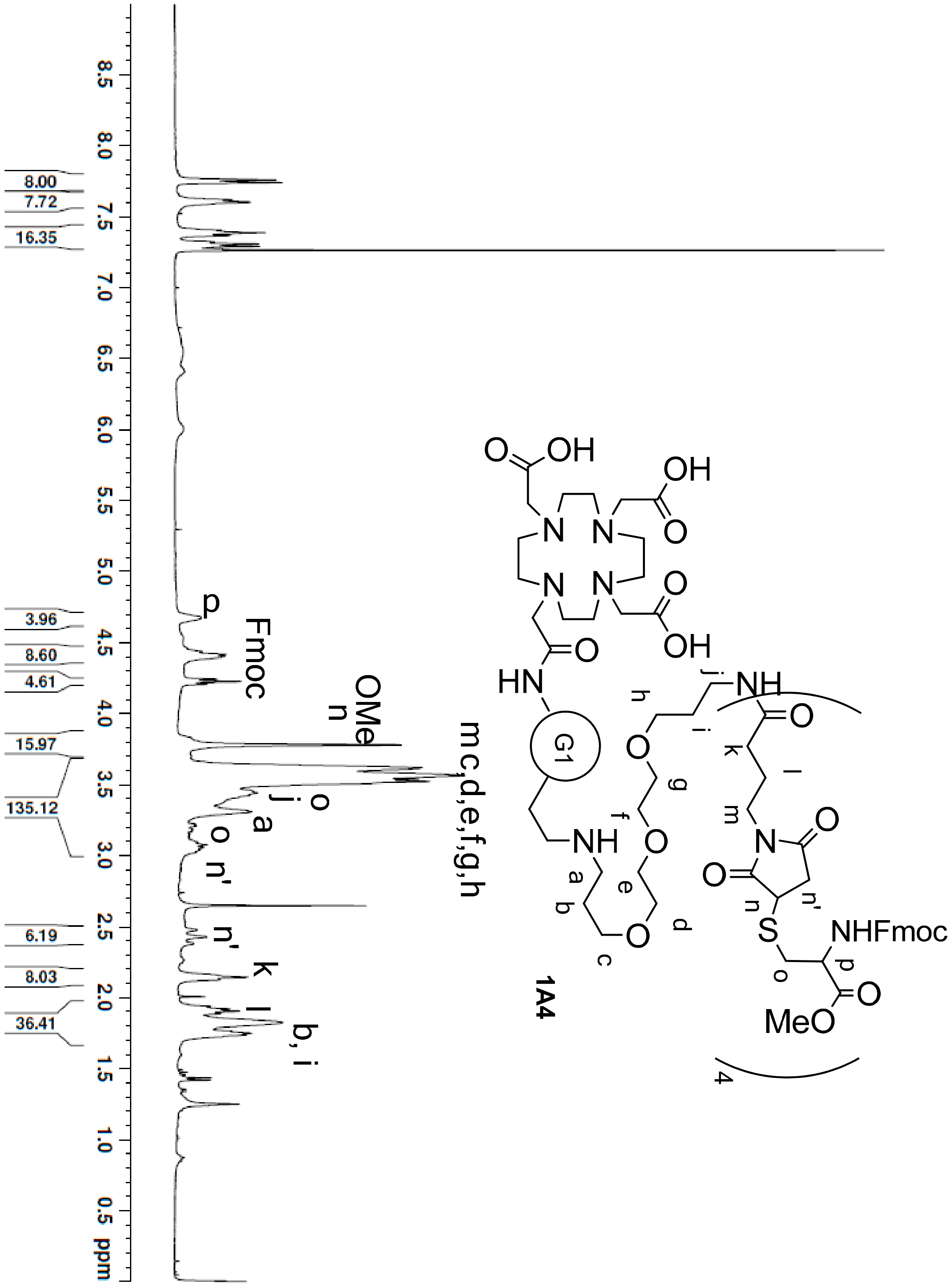

Figure S41. ^{1}H-NMR of **1-A**$_4$ (400 MHz, $CDCl_3$).

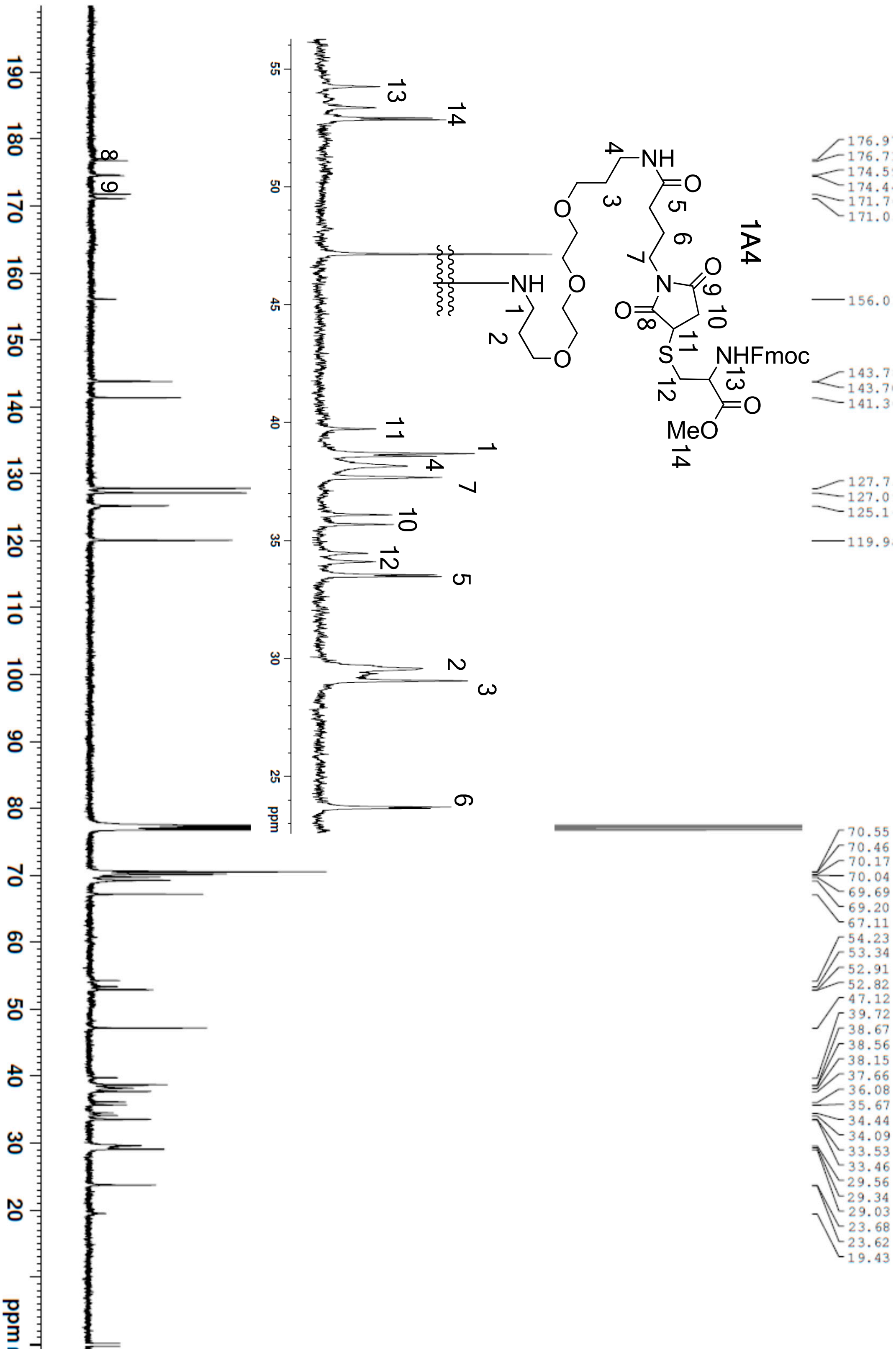

Figure S42. ^{13}C-NMR of **1-A$_4$** (100 MHz, $CDCl_3$).

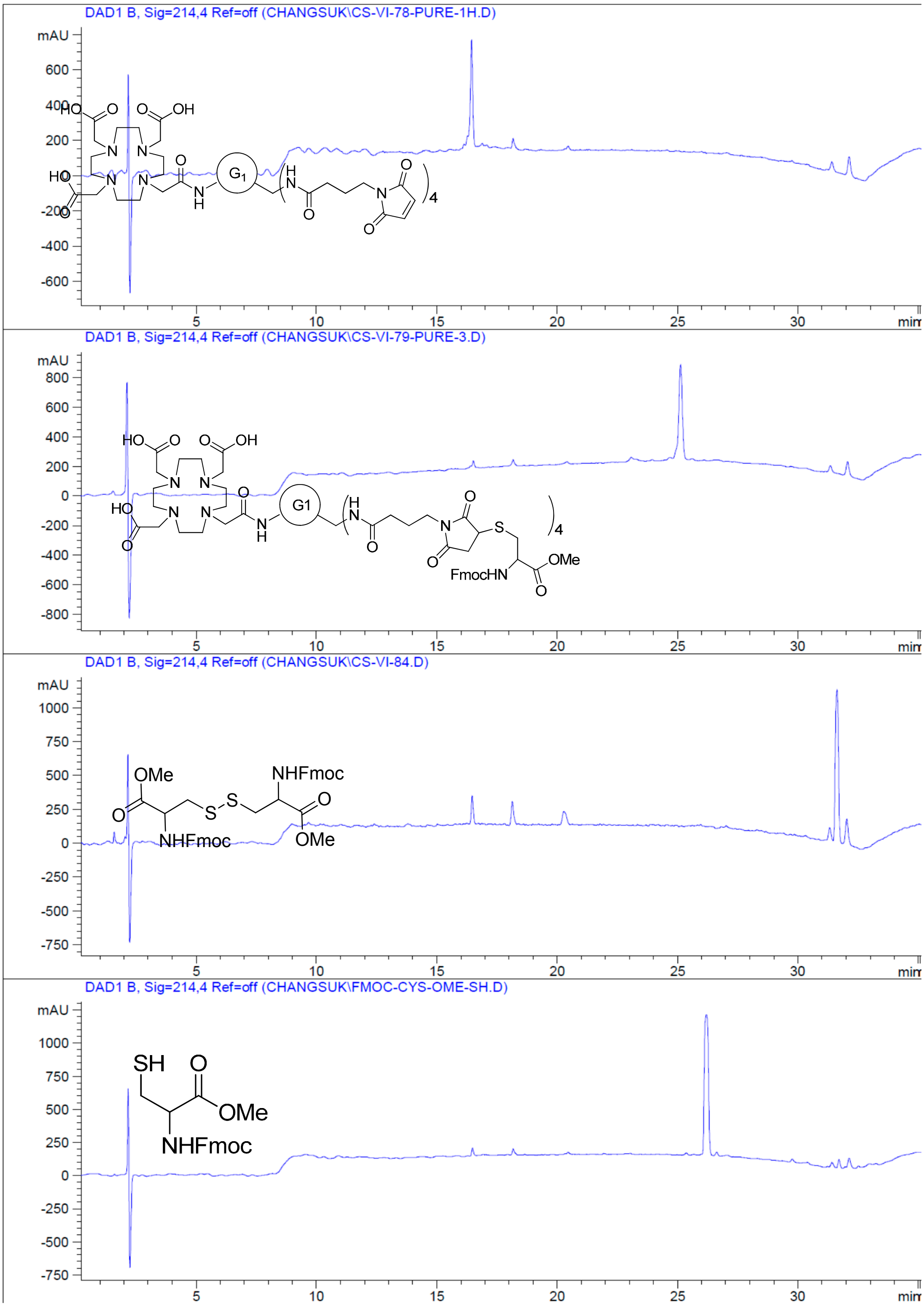

Figure S43. Comparison by HPLC of **1**, **1-A**$_4$, and cysteine. The mobile phase consisted of water/acetonitrile (A/B, HPLC grade, 0.1% (*w*/*v*) trifluoroacetic acid) at a flow rate of 0.8 mL/min. The elution gradient was 10% MeCN for 5 min, ramp to 90% MeCN in 30 min, and ramp down to 10% MeCN in 15 min. The sample volume injected 5 μL at a concentration of 0.1 mg/mL (Acetonitrile) of each samples and eluted sample was detected at 214 nm.

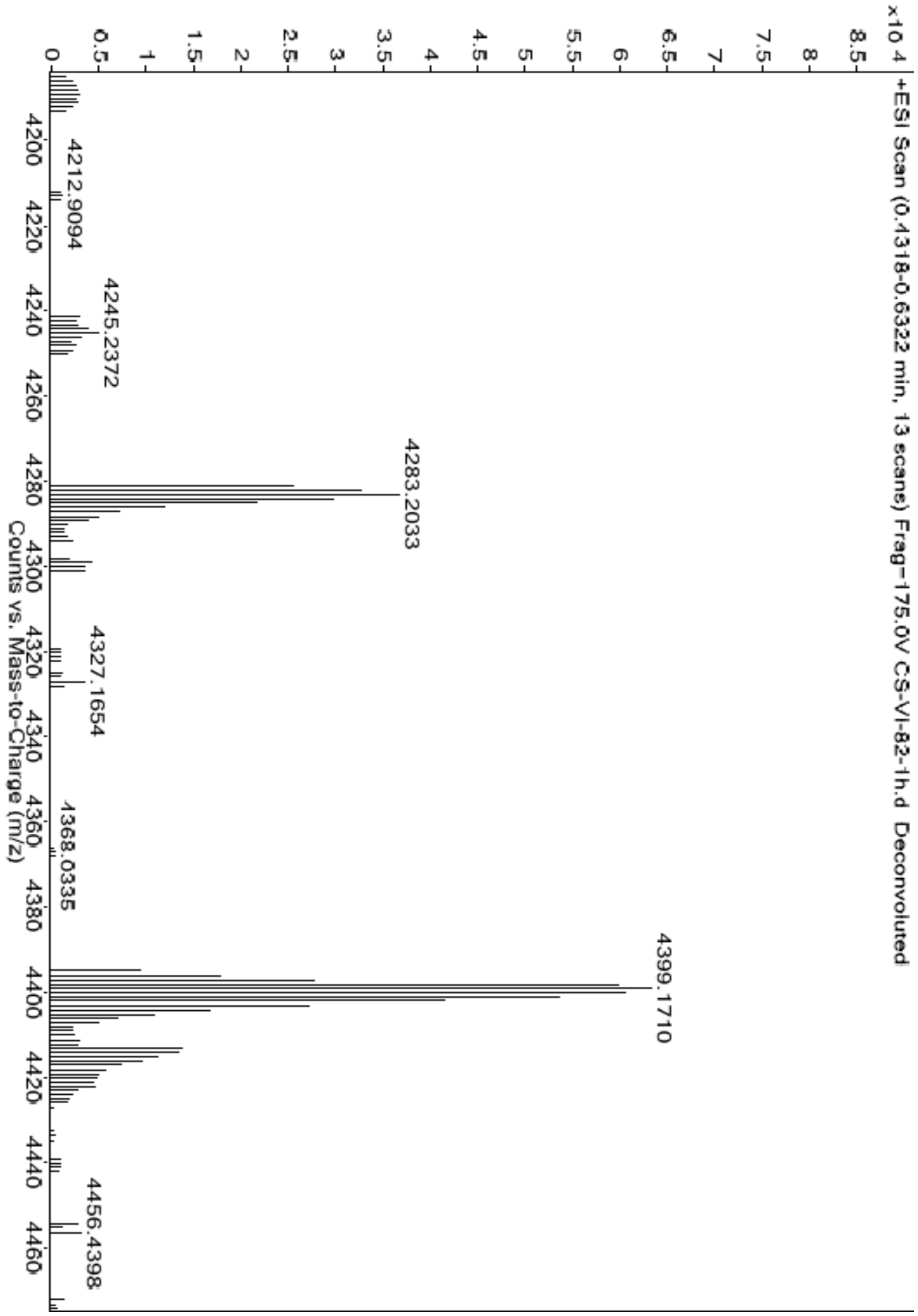

Figure S44. Gd-adduct of **1-A**$_4$ by ESI-TOF MS. Calcd. for $C_{203}H_{289}GdN_{35}O_{56}S_4$ 4398.8966, found 4399.1710 (M + Gd).$^+$ The line at *m*/*z* 4245 is **1-A**$_4$ while *m*/*z* 4283 is the K$^+$ adduct of **1-A**$_4$.

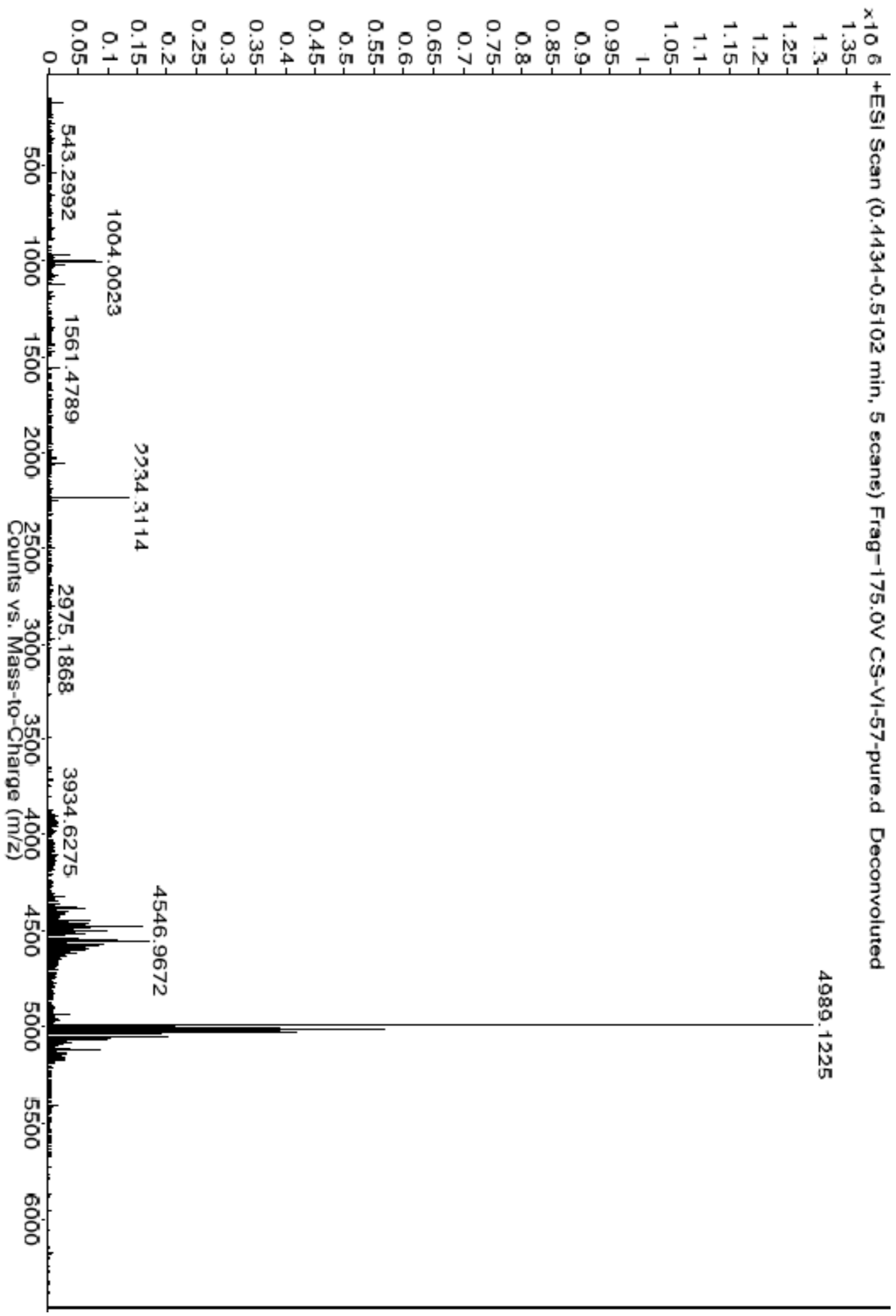

Figure S45. ESI-TOF MS of **1-B$_4$**. The lines identified as *m*/*z* 4546 and 3934 likely correspond to **1-B$_3$** and **1-B$_2$**, respectively.

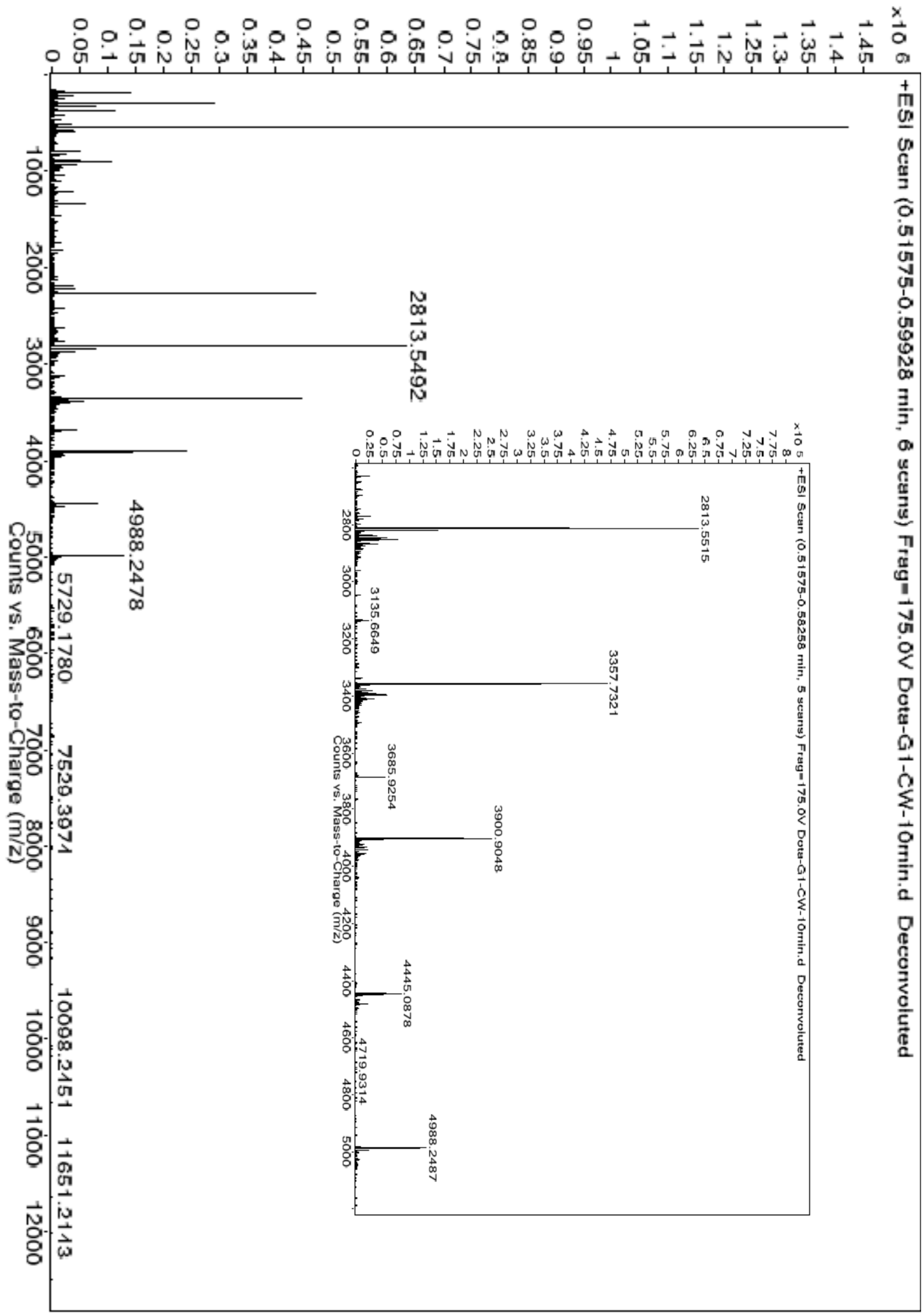

Figure S46. ESI-TOF MS of **1-B**$_4$ after 10 min. The lines identified as *m*/*z* 2813, 3357, 3900, and 4445 likely correspond to **1**, **1-B**$_1$, **1-B**$_2$ and **1-B**$_3$, respectively.

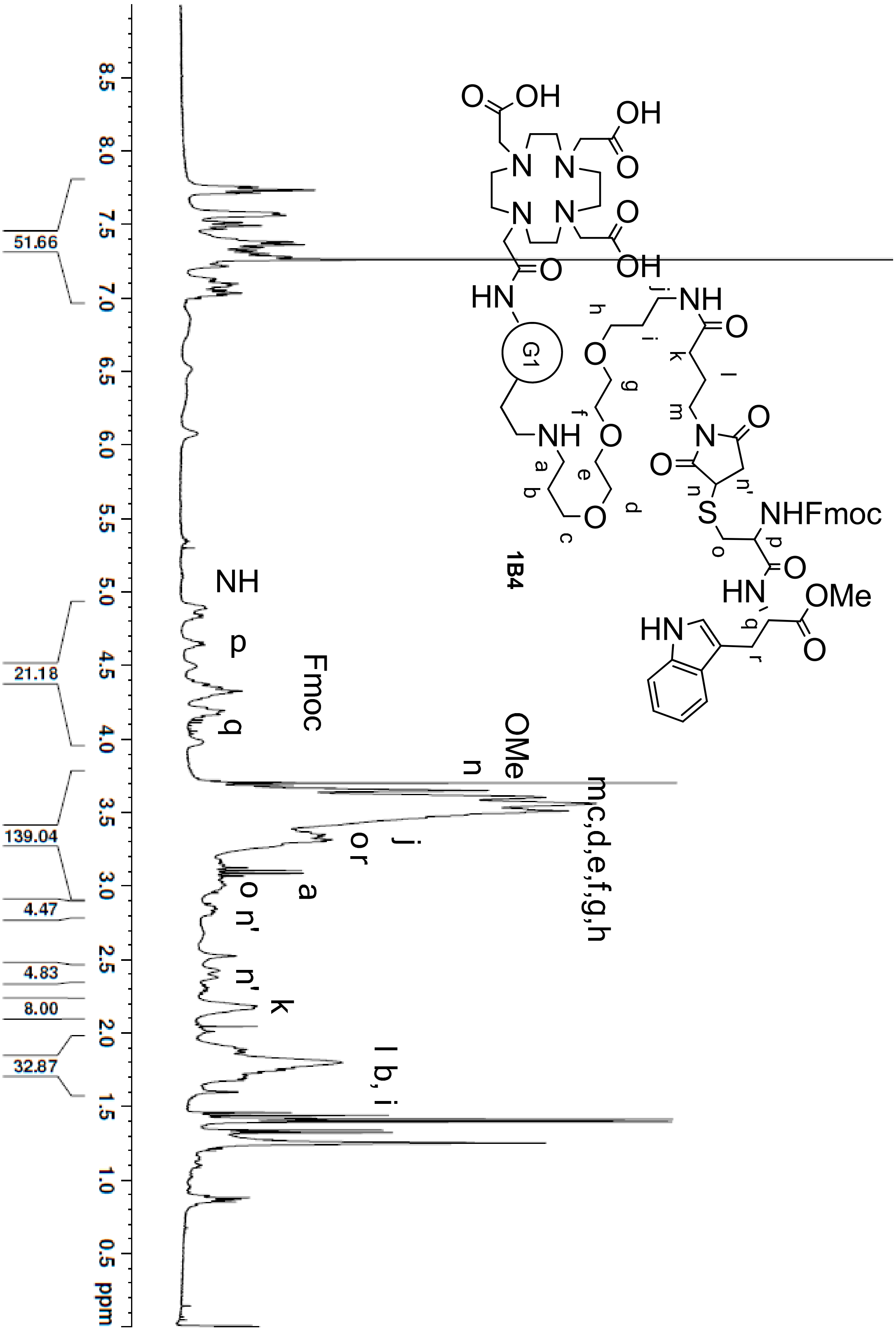

Figure S47. ^{1}H-NMR of **1-B$_4$** (400 MHz, $CDCl_3$).

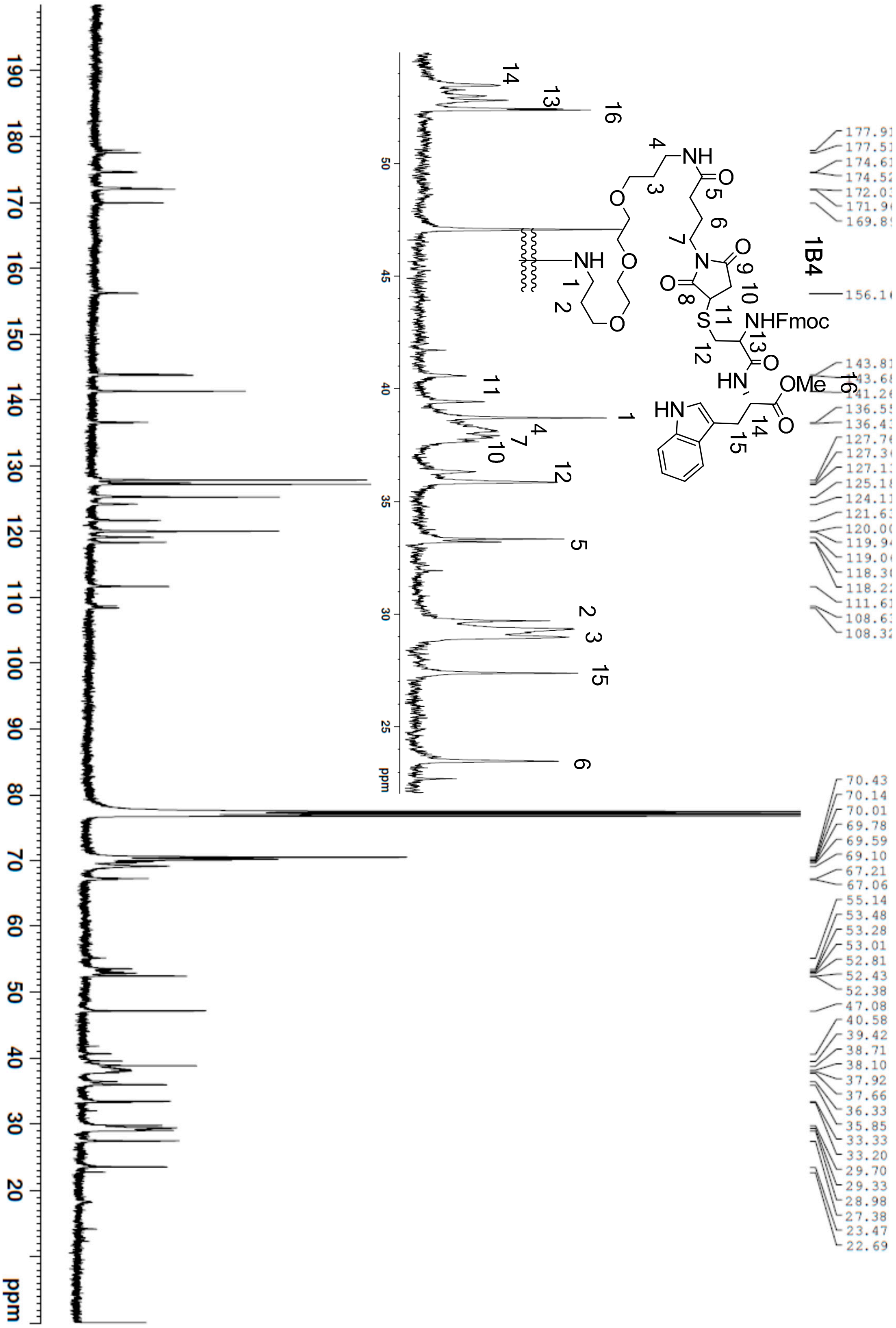

Figure S48. ^{13}C-NMR of **1-B$_4$** (100 MHz, $CDCl_3$).

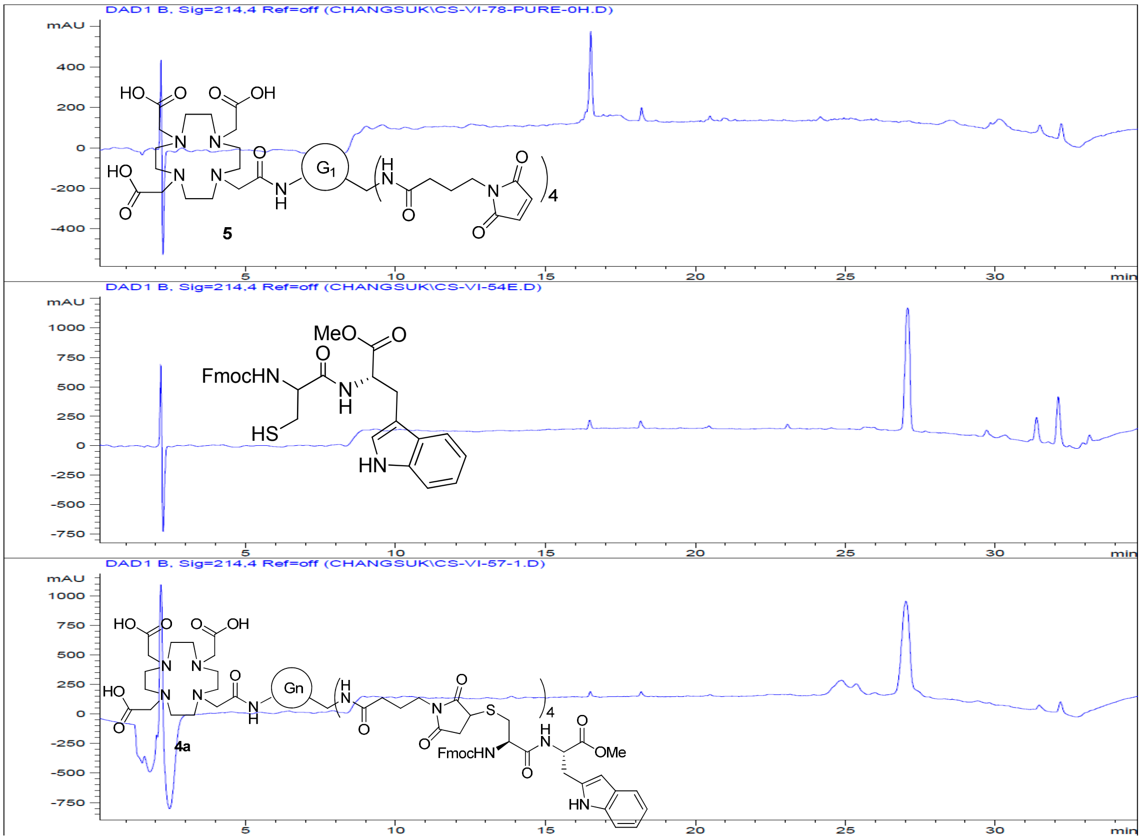

Figure S49. Comparison by HPLC of **1**, **B**, **1-B**4. The mobile phase consisted of water/acetonitrile (A/B, HPLC grade, 0.1% (*w*/*v*) trifluoroacetic acid) at a flow rate of 0.8 mL/min. The elution gradient was 10% MeCN for 5 min, ramp to 90% MeCN in 30 min, and ramp down to 10% MeCN in 15 min. The sample volume injected 5 μL at a concentration of 0.1 mg/mL (Acetonitrile) of each samples and eluted sample was detected at 214 nm.

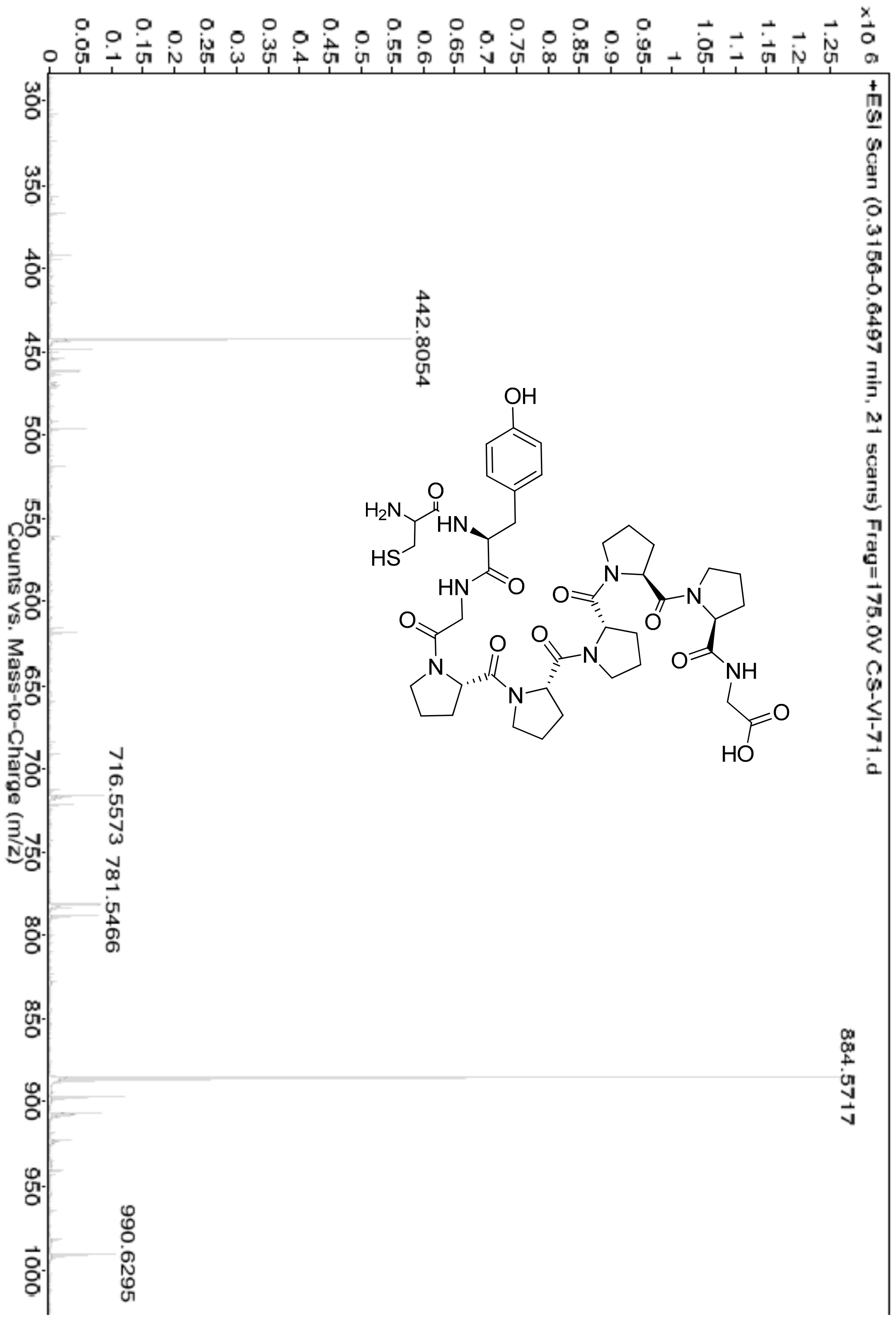

Figure S51. ESI-TOF MS of **1-C**$_4$**.**

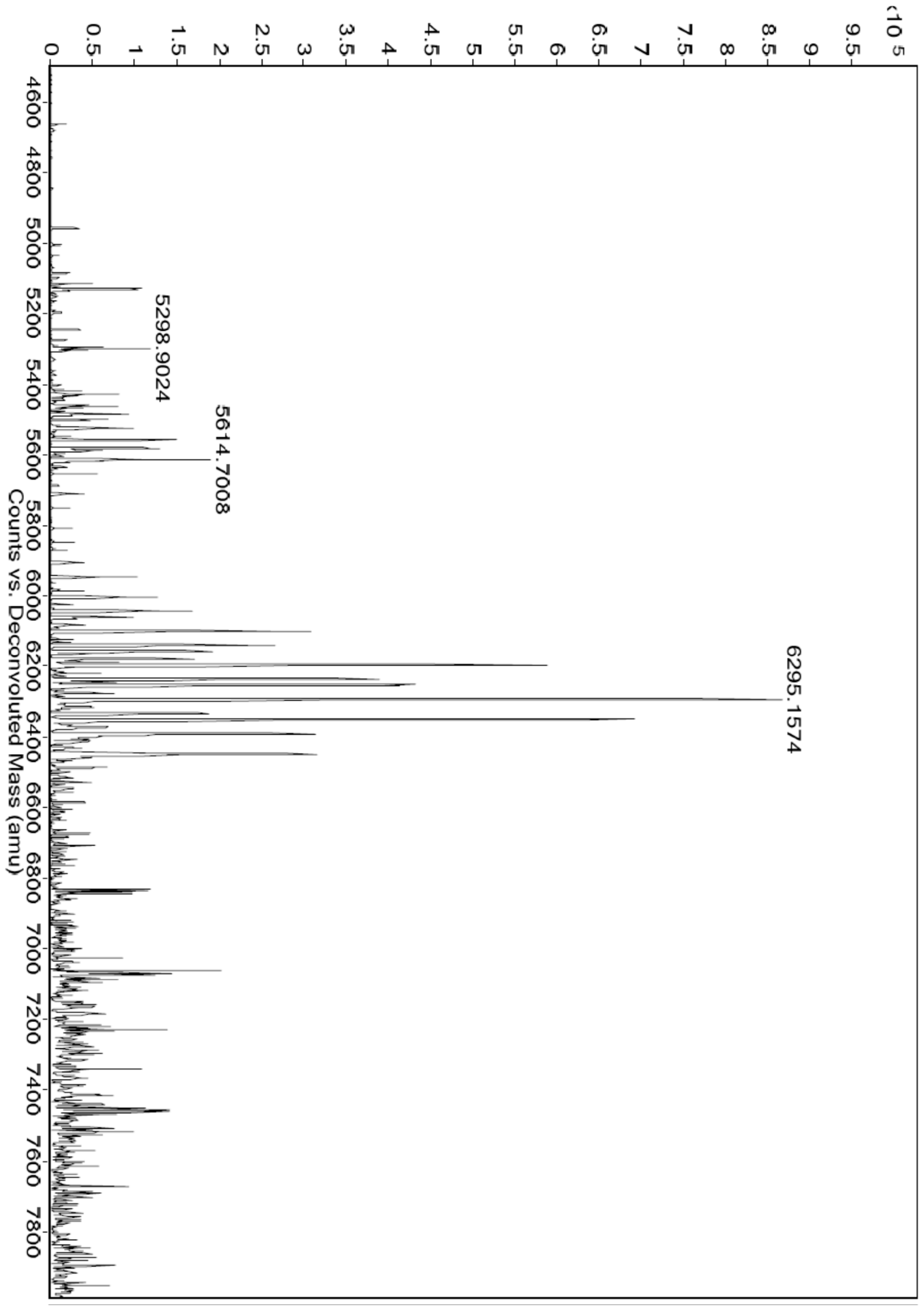

Figure S50. ESI-TOF MS of **C**. **C** is CYGPPPPPG. Calcd for $C_{41}H_{58}N_9O_{11}S$ 884.3977, found 884.5717 $[M + H]^+$ The line at *m*/*z* 442 is the doubly charged species. Lines at *m*/*z* 716 and 781 correspond to sequences missing amino acids.

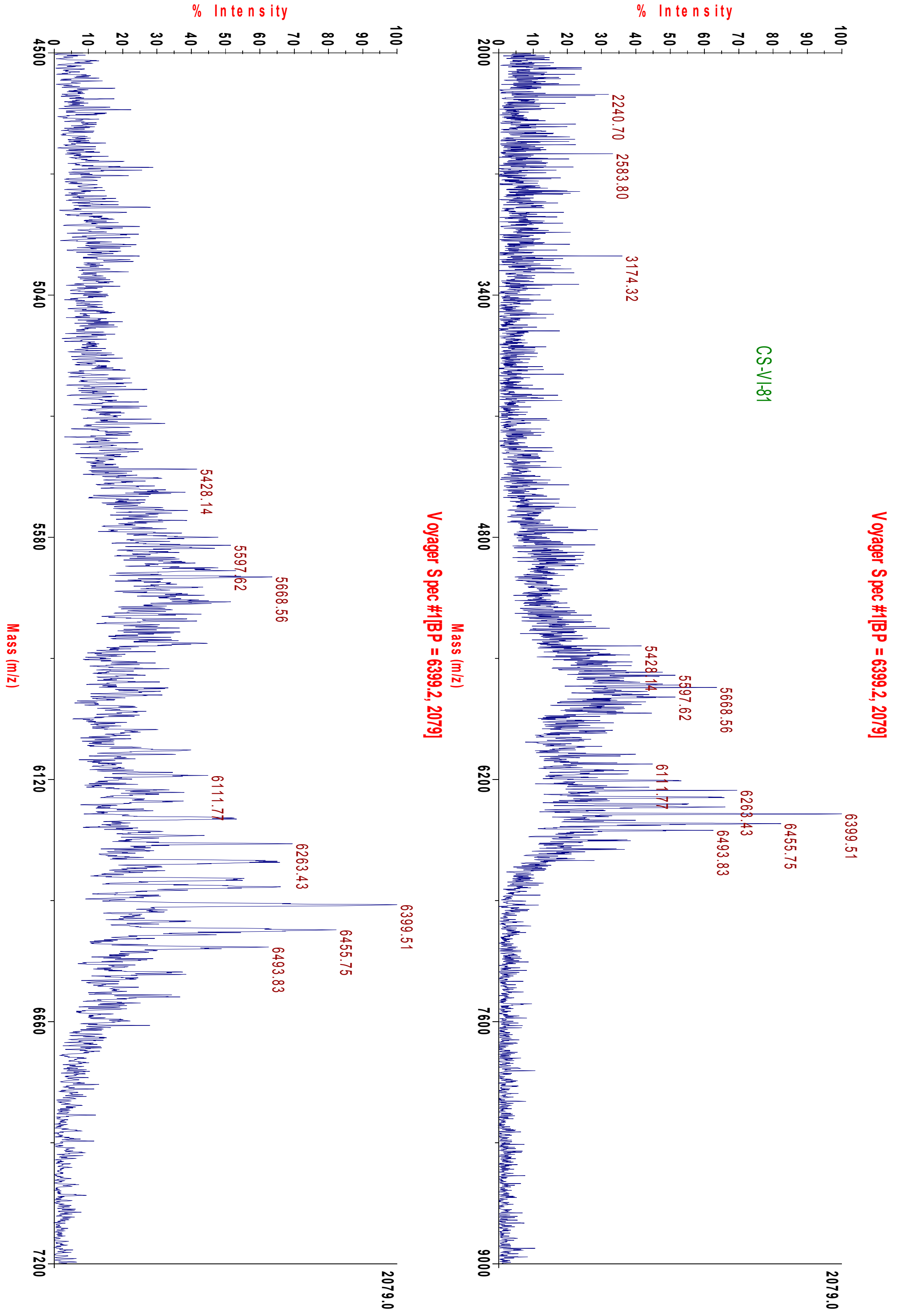

Figure S52. MALDI-TOF MS of **1-C**4.

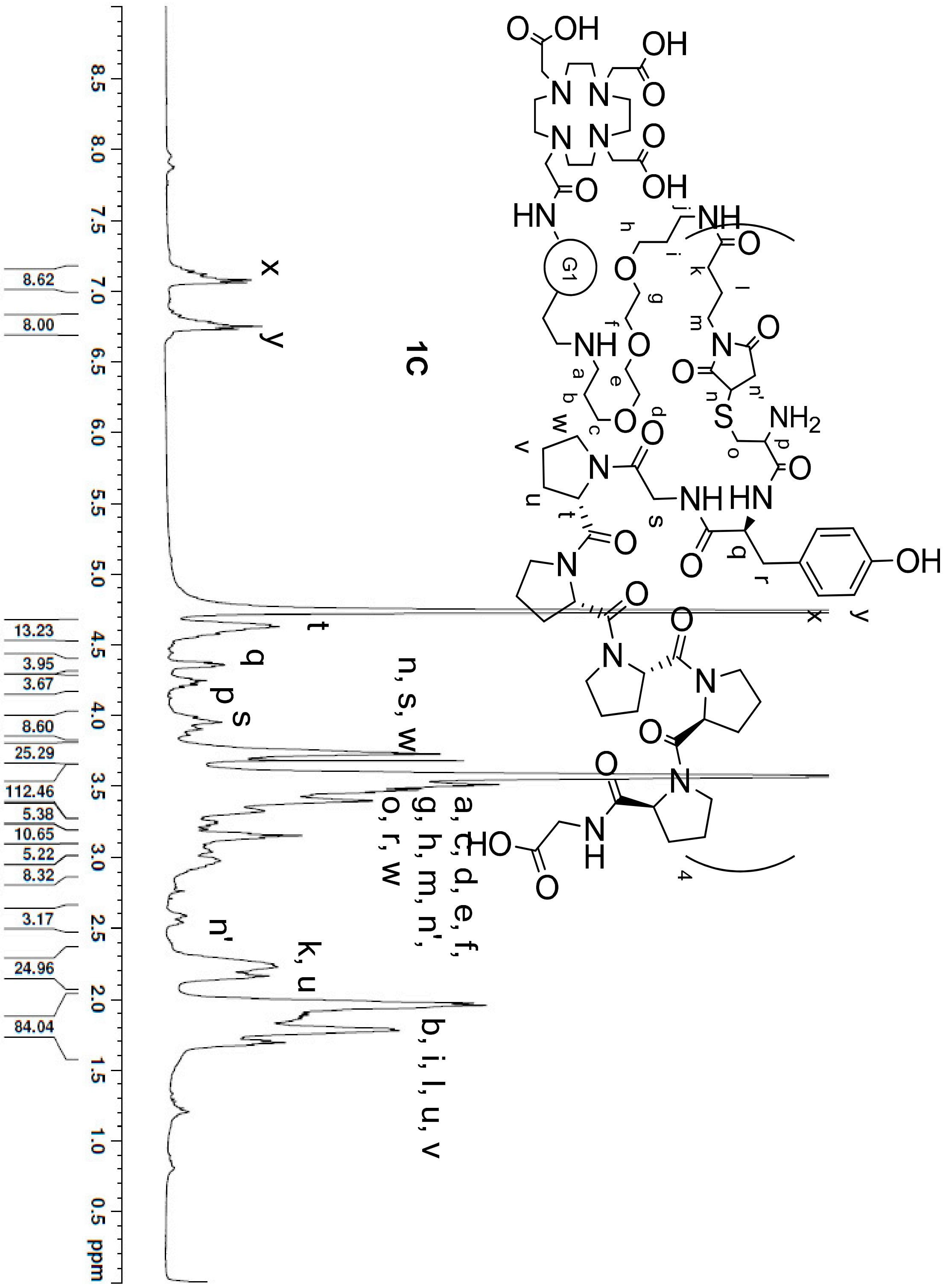

Figure S53. ^{1}H-NMR of **1-C**$_4$ (400 MHz, D_2O).

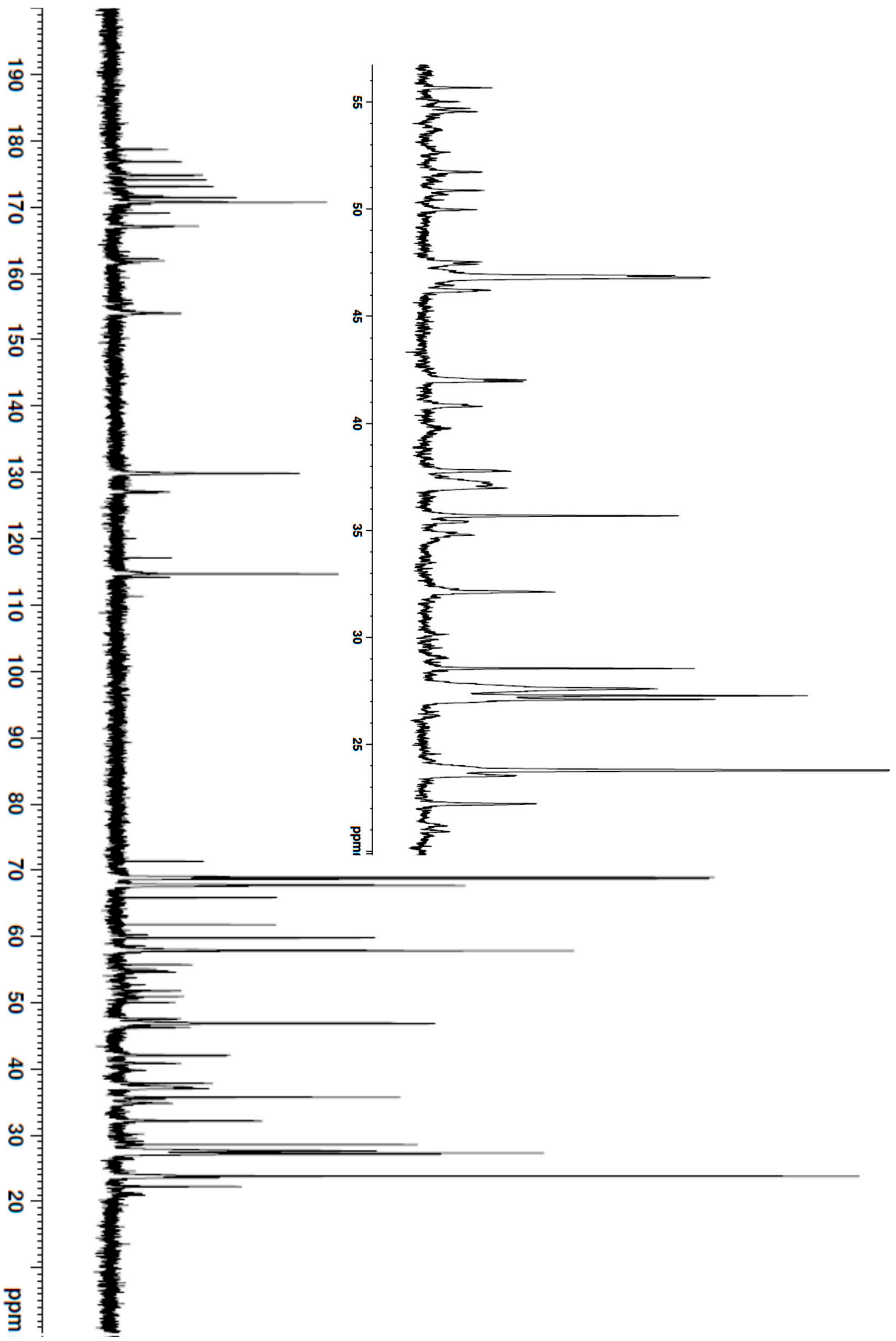

Figure S54. ^{13}C-NMR of **1-C$_4$** (100 MHz, D_2O).

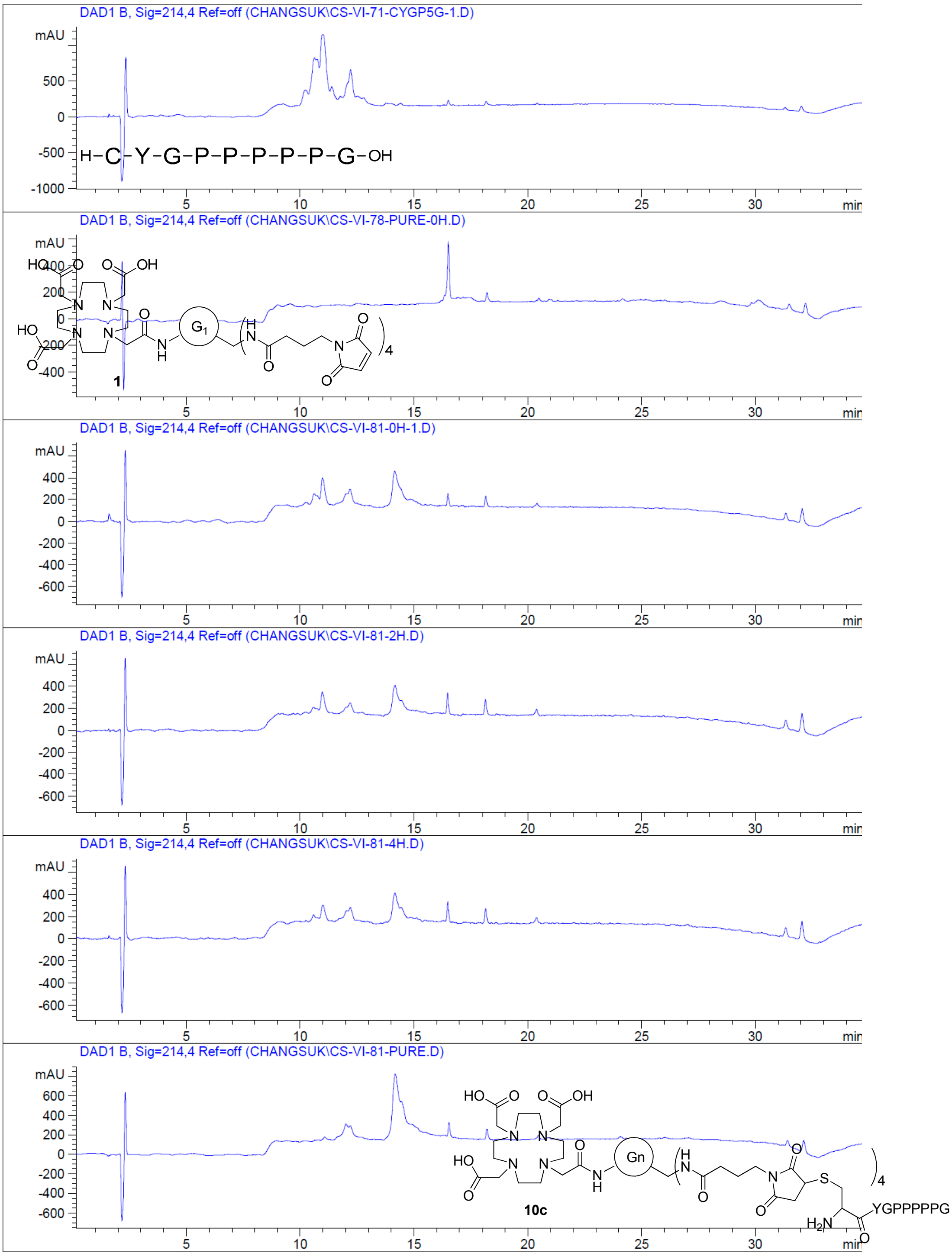

Figure S55. Comparison by HPLC of **1**, **C**, **1-C$_4$**. The mobile phase consisted of water/MeCN (A/B, HPLC grade, 0.1% (*w*/*v*) TFA) at a flow rate of 0.8 mL/min. The elution gradient was 10% MeCN for 5 min, ramp to 90% MeCN in 30 min, and ramp down to 10% MeCN in 15 min. The sample volume injected 5 μL at a concentration of 10 μL of reaction solution and mass up to 0.5 mL with HPLC-grade MeCN, and eluted sample was detected at 214 nm. Panels (from top to bottom) show the crude peptide **C**, **1**, the reaction at 1h, 2h, 4h, and product isolated by membrane filtration.

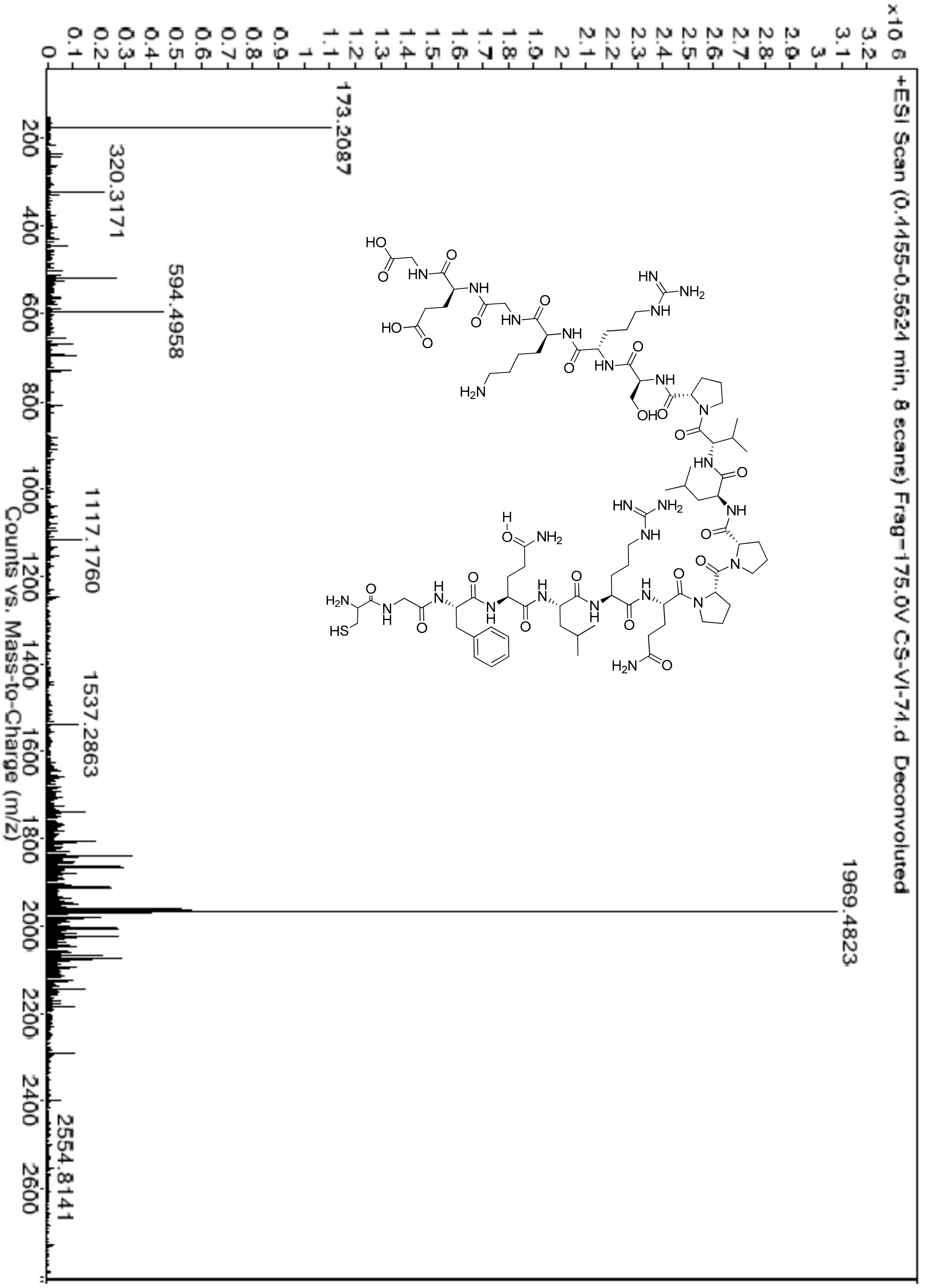

Figure S56. ESI-TOF MS of **D. D** is CGFQLRQPPLVPSRKGEG. Calcd. for $C_{86}H_{142}N_{27}O_{24}S$ 1969.0442, found 1969.4823 $[M + H]^+$.

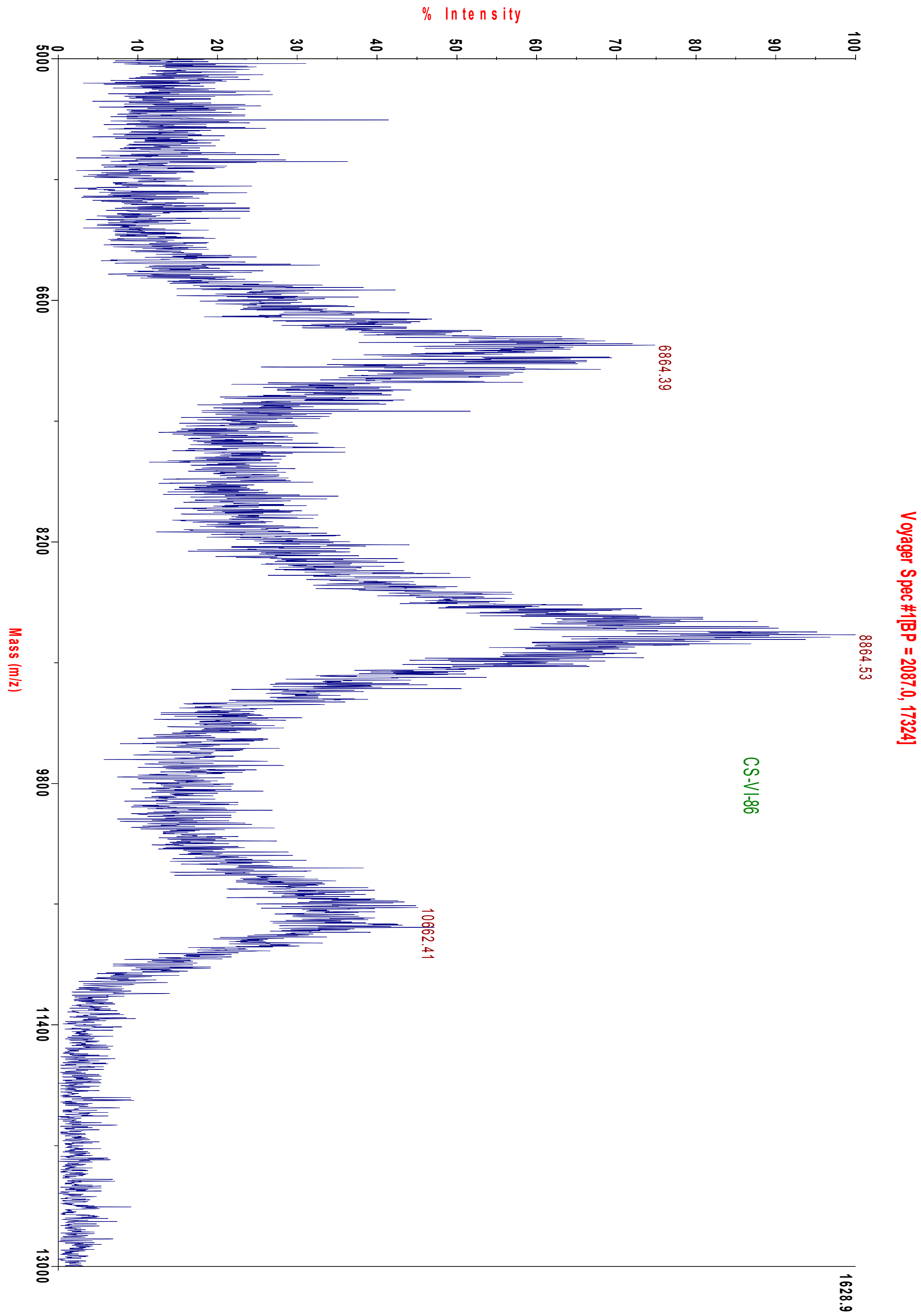

Figure S57. MALDI-TOF MS of **1-D**$_4$. Calcd for $C_{471}H_{777}N_{139}O_{136}S_4$ 10684, found 10662. The other broad peaks likely correspond to **1-D**$_2$ and **1-D**$_3$.

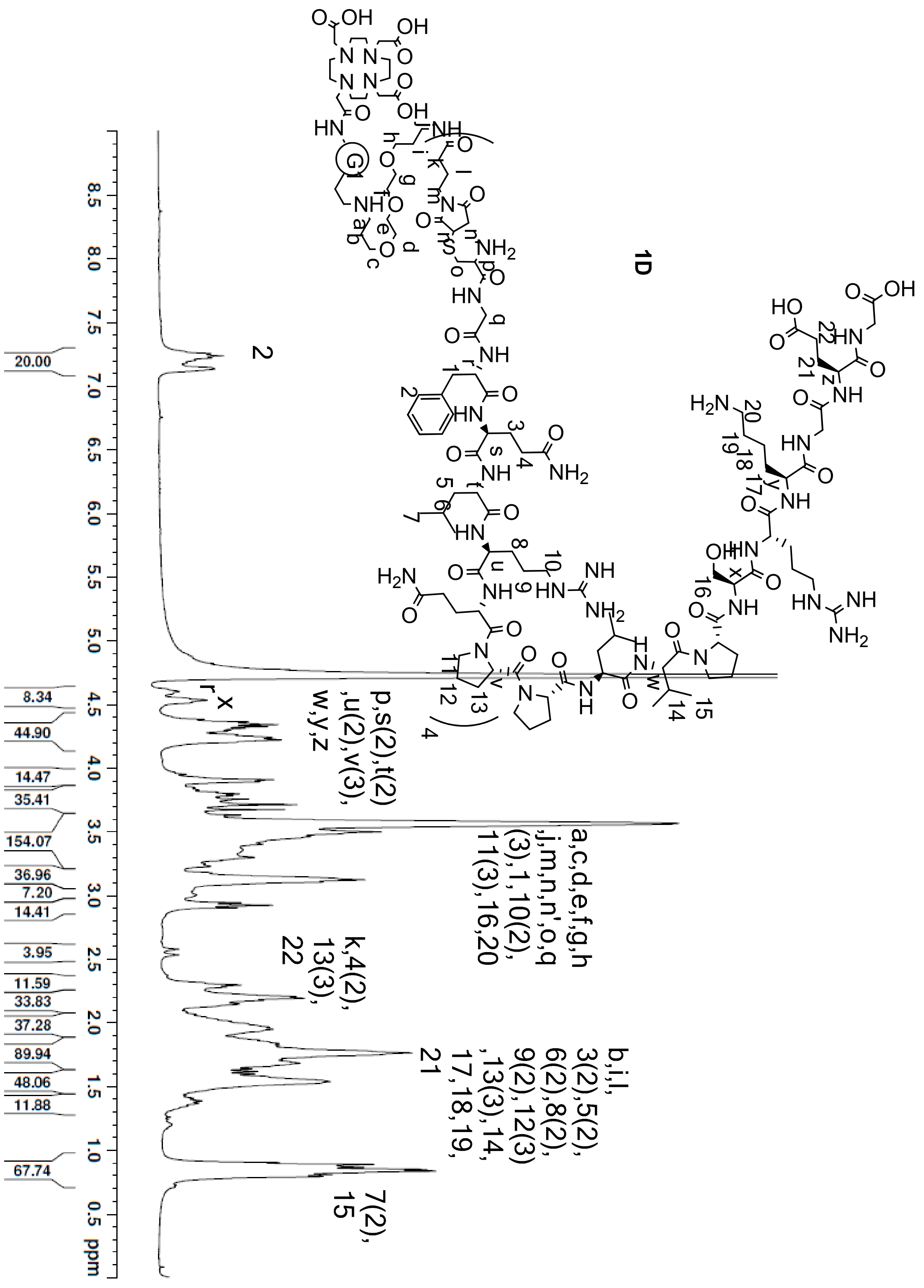

Figure S58. ^{1}H-NMR of **1-D**$_4$ (400 MHz, D_2O).

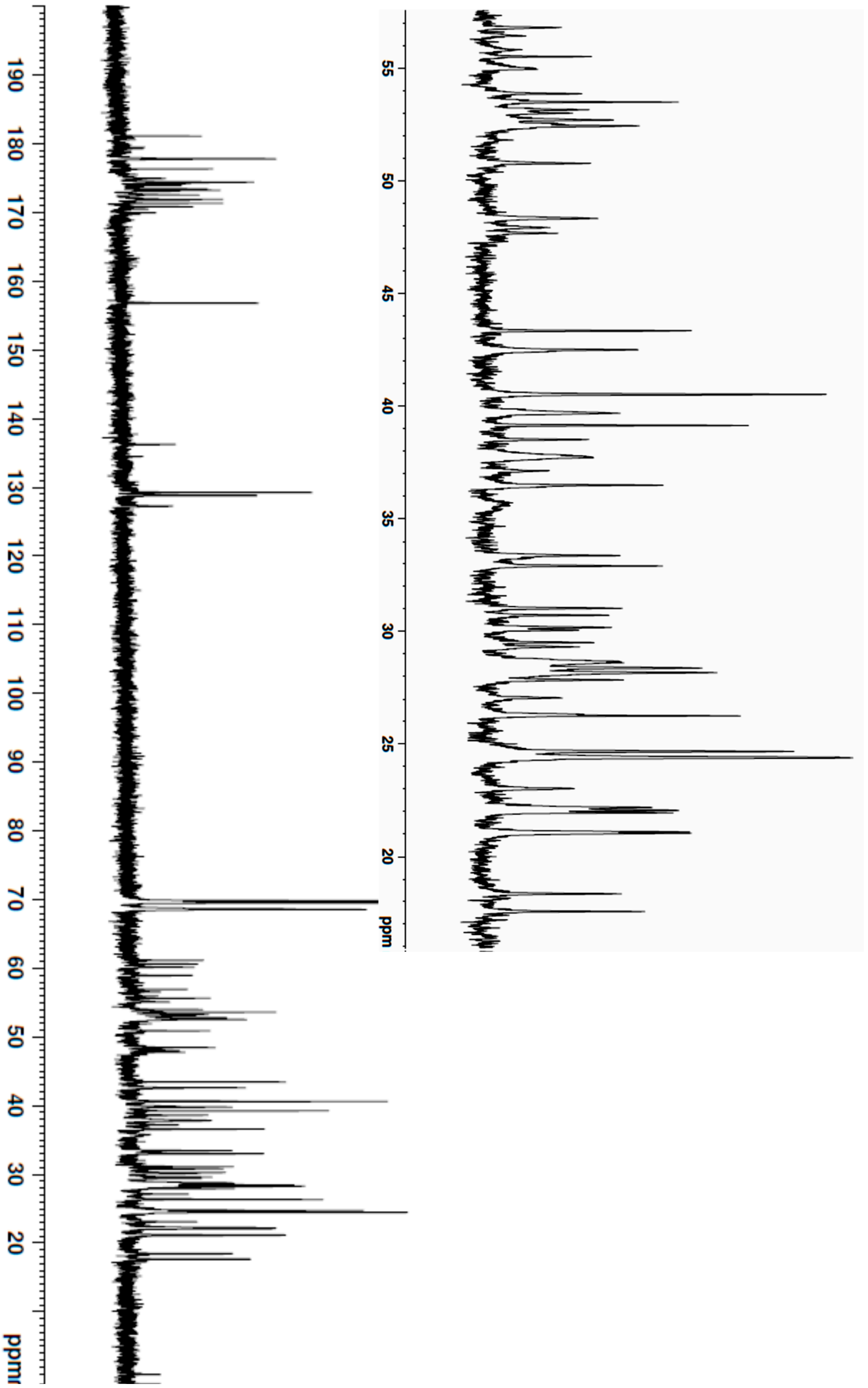

Figure S59. ^{13}C-NMR of **1-D₄** (100 MHz, D_2O).

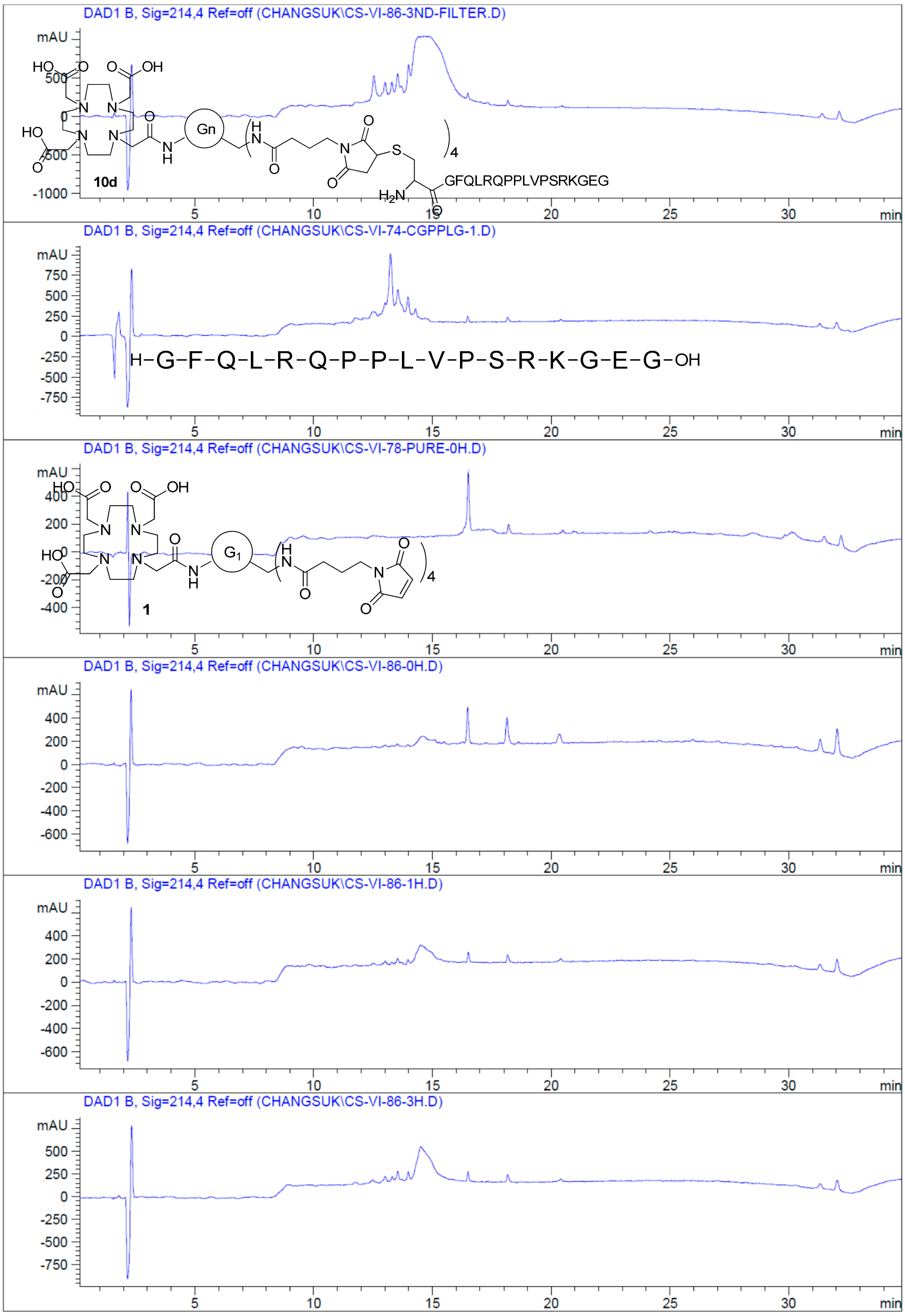

Figure S60. Comparison by HPLC of **1**, **D**, **1-D**$_4$. The mobile phase consisted of water/MeCN (A/B, HPLC grade, 0.1% (*w*/*v*) TFA) at a flow rate of 0.8 mL/min. The elution gradient was 10% MeCN for 5 min, ramp to 90% MeCN in 30 min, and ramp down to 10% MeCN in 15 min. The sample volume injected 5 μL at a concentration of 10 μL of reaction solution and mass up to 0.5 mL with HPLC-grade MeCN, and eluted sample was detected at 214 nm. Panels correspond to purified **1-D**$_4$**, D**, **1**, and the reaction at 0 h, 1 h, and 3 h.
